# Integrated multiomics analysis of hepatoblastoma unravels its heterogeneity and provides novel druggable targets

**DOI:** 10.1038/s41698-020-0125-y

**Published:** 2020-07-07

**Authors:** Masahiro Sekiguchi, Masafumi Seki, Tomoko Kawai, Kenichi Yoshida, Misa Yoshida, Tomoya Isobe, Noriko Hoshino, Ryota Shirai, Mio Tanaka, Ryota Souzaki, Kentaro Watanabe, Yuki Arakawa, Yasuhito Nannya, Hiromichi Suzuki, Yoichi Fujii, Keisuke Kataoka, Yuichi Shiraishi, Kenichi Chiba, Hiroko Tanaka, Teppei Shimamura, Yusuke Sato, Aiko Sato-Otsubo, Shunsuke Kimura, Yasuo Kubota, Mitsuteru Hiwatari, Katsuyoshi Koh, Yasuhide Hayashi, Yutaka Kanamori, Mureo Kasahara, Kenichi Kohashi, Motohiro Kato, Takako Yoshioka, Kimikazu Matsumoto, Akira Oka, Tomoaki Taguchi, Masashi Sanada, Yukichi Tanaka, Satoru Miyano, Kenichiro Hata, Seishi Ogawa, Junko Takita

**Affiliations:** 1 Department of Pediatrics, Graduate School of Medicine, The University of Tokyo, Tokyo, Japan; 2 Department of Maternal-Fetal Biology, National Research Institute for Child Health and Development, Tokyo, Japan; 3 Department of Pathology and Tumor Biology, Graduate School of Medicine, Kyoto University, Kyoto, Japan; 4 Department of Pediatric Surgery, The University of Tokyo Hospital, Tokyo, Japan; 5 Children’s Cancer Center, National Center for Child Health and Development, Tokyo, Japan; 6 Department of Pathology, Kanagawa Children’s Medical Center, Kanagawa, Japan; 7 Department of Pediatric Surgery, Faculty of Medical Sciences, Kyushu University, Fukuoka, Japan; 8 Department of Hematology/Oncology, Saitama Children’s Medical Center, Saitama, Japan; 9 Department of Urology, Graduate School of Medicine, The University of Tokyo, Tokyo, Japan; 10 Division of Molecular Oncology, National Cancer Center Research Institute, Tokyo, Japan; 11 Center for Cancer Genomics and Advanced Therapeutics, National Cancer Center Research Institute, Tokyo, Japan; 12 Laboratory of DNA Information Analysis, Human Genome Center, The Institute of Medical Science, The University of Tokyo, Tokyo, Japan; 13 Department of Systems Biology, Graduate School of Medicine, Nagoya University, Nagoya, Japan; 14 Department of Pediatrics, Graduate School of Biomedical Sciences, Hiroshima University, Hiroshima, Japan; 15 Jobu University, Gunma, Japan; 16 Division of Surgery, Department of Surgical Specialties, National Center for Child Health and Development, Tokyo, Japan; 17 Transplantation Center, National Center for Child Health and Development, Tokyo, Japan; 18 Department of Anatomic Pathology, Graduate School of Medical Sciences, Kyushu University, Fukuoka, Japan; 19 Department of Pathology, National Center for Child Health and Development, Tokyo, Japan; 20 Department of Advanced Diagnosis, Clinical Research Center, Nagoya Medical Center, Nagoya, Japan; 21 Institute for the Advanced Study of Human Biology (WPI-ASHBi), Kyoto, Japan; 22 Department of Medicine, Center for Hematology and Regenerative Medicine, Karolinska Institute, Stockholm, Sweden; 23 Department of Pediatrics, Graduate School of Medicine, Kyoto University, Kyoto, Japan

**Keywords:** Paediatric cancer, Liver cancer, Cancer genomics, Next-generation sequencing

## Abstract

Although hepatoblastoma is the most common pediatric liver cancer, its genetic heterogeneity and therapeutic targets are not well elucidated. Therefore, we conducted a multiomics analysis, including mutatome, DNA methylome, and transcriptome analyses, of 59 hepatoblastoma samples. Based on DNA methylation patterns, hepatoblastoma was classified into three clusters exhibiting remarkable correlation with clinical, histological, and genetic features. Cluster F was largely composed of cases with fetal histology and good outcomes, whereas clusters E1 and E2 corresponded primarily to embryonal/combined histology and poor outcomes. E1 and E2, albeit distinguishable by different patient age distributions, were genetically characterized by hypermethylation of the HNF4A/CEBPA-binding regions, fetal liver-like expression patterns, upregulation of the cell cycle pathway, and overexpression of *NQO1* and *ODC1*. Inhibition of *NQO1* and *ODC1* in hepatoblastoma cells induced chemosensitization and growth suppression, respectively. Our results provide a comprehensive description of the molecular basis of hepatoblastoma and rational therapeutic strategies for high-risk cases.

## Introduction

Hepatoblastoma is the most common pediatric liver cancer that mainly affects young children^1^. This disease is clinically heterogeneous, and although the treatment outcome of hepatoblastoma has improved with the overall survival reaching ~80%^2^, the prognosis of high-risk cases with unfavorable prognostic factors is still poor despite high-intensity therapy^3^. Known poor prognostic factors include larger tumor extension (the pretreatment extent of tumor [PRETEXT] stage IV), presence of metastasis, extremely high or low tumor marker level (serum alpha-fetoprotein level > 1,000,000 ng/mL or <100 ng/mL), and older age (>2 years)^3^. As intensification of the chemotherapy applied to such high-risk cases is reaching a limit, novel therapeutic approaches based on the understanding of the biological mechanisms are required to overcome high-risk hepatoblastoma.

The genetic hallmark of hepatoblastoma is aberrant activation of Wnt signaling pathway^4–6^, and several studies that addressed the genomic profile of hepatoblastoma revealed the high prevalence of Wnt-activating mutations shared by most hepatoblastoma cases^7–10^. However, genetic determinants of the clinical heterogeneity of this cancer are still unclear. The prognostic biomarkers suggested in the previous studies not only have limitations in the reproducibility of their correlation with treatment outcomes, but their biological implications are also not clearly understood^7,9,11^. For example, Cairo et al. described the molecular classification of hepatoblastoma into two subclasses, C1 and C2, using a 16-gene signature, with C2 being a group with poor prognosis in the cohort^7^. However, Sumazin et al. revealed that the classification was not prognostically predictive in another cohort^9^.

In addition, hepatoblastoma shows one of the lowest mutation burdens among all cancers^12^. Whole-exome sequencing of hepatoblastoma revealed an average of less than five mutations per tumor^8,9,13^. The very low frequency of mutations has hindered the discovery of possible therapeutic targets for hepatoblastoma.

To elucidate these issues, we performed a multiomics analysis, including mutatome, DNA methylome, and transcriptome analyses, of 59 hepatoblastoma samples to generate comprehensive genetic profiles, determine the genetic heterogeneity of this disease, and identify specific therapeutic targets (Supplementary Tables 1 and 2).

## Results

### Profiles of gene mutations and copy number (CN) alterations

**Fig. 1:**
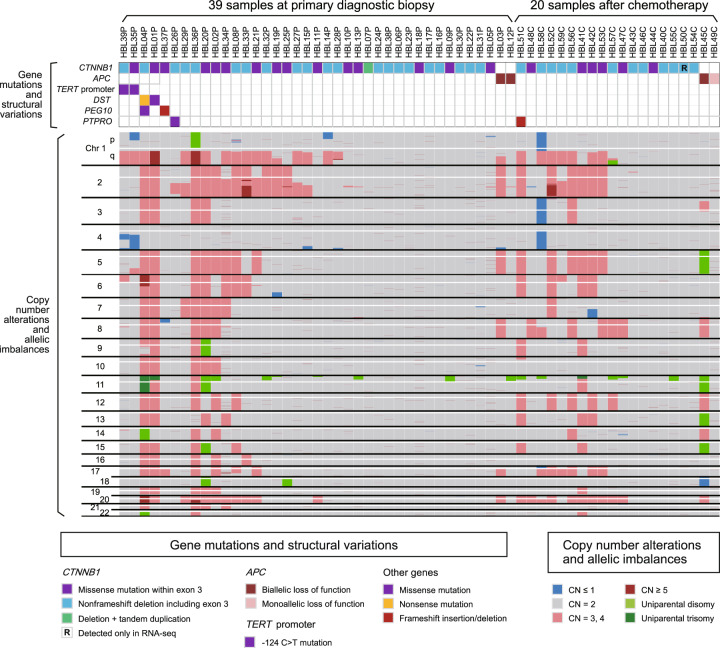
Landscape of genetic alterations in hepatoblastoma. Recurrent gene mutations, copy number (CN) alterations, and allelic imbalances are shown.

First, we performed mutation analysis by targeted capture sequencing (Target-seq) and single-nucleotide polymorphism (SNP) array-based CN analysis. We detected a total of 76 somatic alterations by Target-seq (Supplementary Tables 3 and 4 and Fig. 1). Among the driver mutations, *CTNNB1* (encoding beta-catenin) alterations were detected in 54 of the 59 samples (92%); all alterations were associated with exon 3 (Supplementary Figs. 1a and 2) and were reported to induce beta-catenin stabilization and hepatoblastoma tumorigenesis^6,14^. In addition, four samples harbored germline truncating mutations in *APC*, another well-known hepatoblastoma driver gene^15,16^, of which three were accompanied with additional somatic *APC* alterations (Supplementary Fig. 1b). In the remaining one sample where we could not detect *CTNNB1/APC* alterations in Target-seq (HBL50C), a 15-base-pair non-frameshift deletion within exon 3 of *CTNNB1* (c.83_94del) was identified in RNA sequencing (RNA-seq). In total, driver mutations in *CTNNB1/APC* were identified in all the 59 samples. In contrast, mutations in genes other than *CTNNB1/APC* were less frequent: *TERT* promoter, *DST*, *PEG10*, and *PTPRO* were mutated in only two samples each (3%) and the others were nonrecurrent (Supplementary Table 5).

Among the CN alterations and allelic imbalances, whole-arm CN gains were more frequent than losses (Supplementary Fig. 3). In addition, 18 of the 59 samples (31%) harbored uniparental disomy/trisomy on chromosome 11 (Supplementary Fig. 4).

These genetic profiles of hepatoblastoma were consistent with previous reports^7–9,17^, and there was no significant difference in the frequency of gene mutations and CN alterations between the biopsy and postchemotherapy samples. Although the importance of beta-catenin-stabilizing mutations was reconfirmed by the high mutation rate of *CTNNB1/APC*, the presence of the other genetic lesions was not sufficient to explain the clinical heterogeneity of hepatoblastoma.

### DNA methylation-based classification of hepatoblastoma

**Fig. 2:**
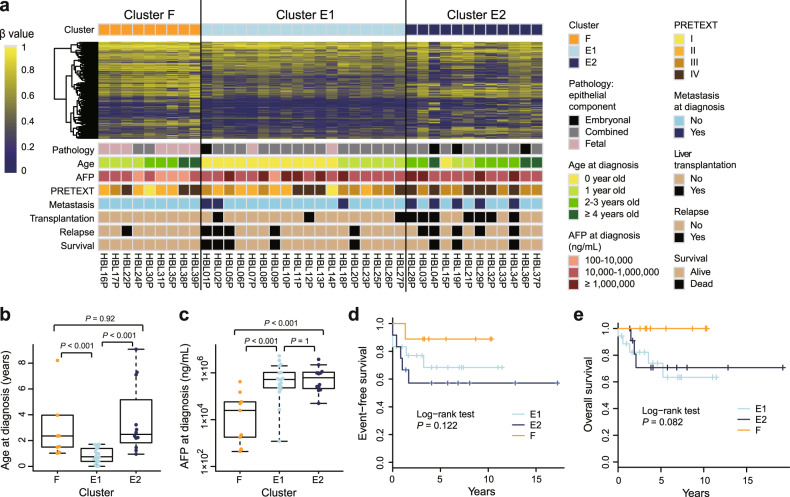
Three hepatoblastoma methylation clusters, F, E1, and E2, display distinct clinical features. **a** DNA methylation heatmap across 39 biopsy samples and clinical information on each case. The heatmap is constructed for the 3000 probes used in the first-step consensus clustering of the 39 samples (Supplementary Data S5). Comparison of age (**b**) and serum alpha-fetoprotein levels (**c**) at diagnosis among the methylation clusters using the Wilcoxon rank-sum test. For the box plots, the middle line is the median, the top and bottom of the box indicate the interquartile range, and the error bars are minimum and maximum values excluding outliers. Kaplan–Meier survival curves of three methylation clusters for event-free (**d**) and overall (**e**) survival. AFP alpha-fetoprotein, PRETEXT pretreatment extent of disease.

To further illustrate the molecular basis, we conducted a microarray-based DNA methylome analysis and performed consensus clustering of the methylation data. We failed to make a robust and meaningful clustering of 59 hepatoblastoma samples (Supplementary Fig. 5) due to a bias inherent in the global methylation status of postchemotherapy samples with reference to the biopsy samples. Therefore, we selected 39 biopsy samples for further clustering analysis. Consensus clustering classified these 39 samples into two stable clusters, F and E, corresponding primarily to cases with fetal and embryonal/combined histology, respectively (Supplementary Fig. 6a–c). Furthermore, the second-step consensus clustering divided cluster E into two subgroups, E1 and E2, which corresponded to younger and older cases, respectively, with ~2 years of age as the border (Supplementary Fig. 6d–f). These methylation clusters were correlated to the other clinical features as well (Fig. 2). Of note, clusters E1/E2 were characterized by higher alpha-fetoprotein levels at diagnosis, frequent presence of metastasis, and worse outcomes compared with cluster F. Liver transplantation was most frequent in cluster E2, followed by clusters E1 and F. These differences suggested distinct biological mechanisms underlying these three clusters. Although the mutation profiles were not significantly different among the clusters, the CN gains, especially of chromosomes 1q and 2, were observed most frequently in cluster E2 (Supplementary Fig. 7).

**Fig. 3:**
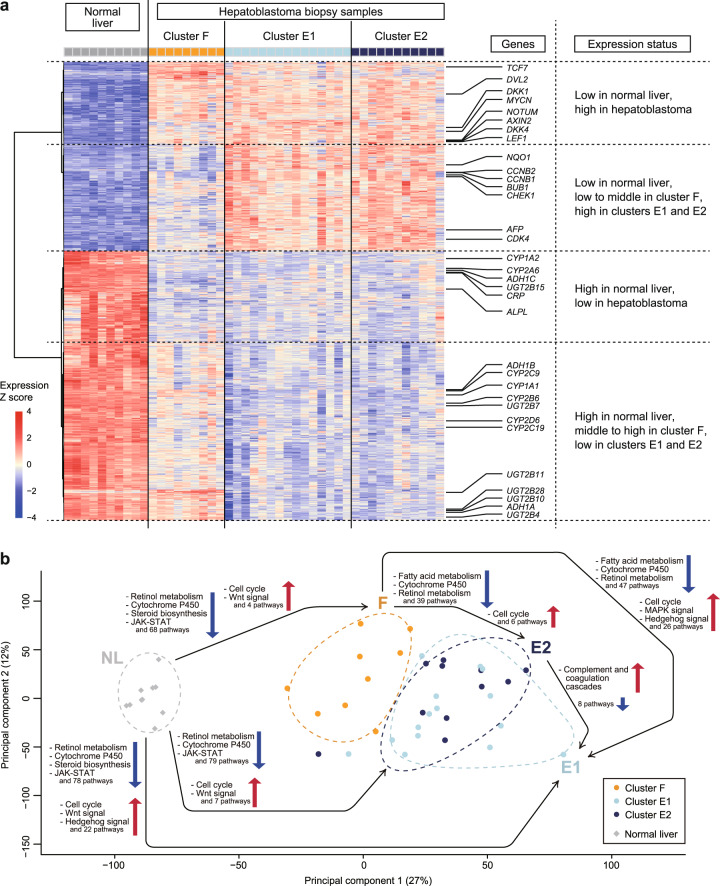
Gene expression analysis of hepatoblastoma and normal liver (NL) reveals similarity between the hepatoblastoma clusters E1 and E2 as well as a relative proximity of cluster F to the NL. **a** Heatmap of the expression data of 2000 differentially expressed genes among four clusters (NL and three hepatoblastoma clusters F, E1, and E2; Supplementary Data S8) across 35 hepatoblastoma biopsy samples and ten NL samples. The expression level is log-transformed and z-normalized to zero mean and unit standard deviation for each gene. **b** Principal component analysis plot for the expression data. Summarized results of the pathway analysis of differential expression among the hepatoblastoma and NL clusters are added on the plot; a black arrow directed from cluster X to Y and pathway A described nearby with an upward/downward arrow indicates significant upregulation/downregulation of pathway A in cluster Y compared with cluster X.

To further characterize the methylation clusters, we performed RNA-seq on hepatoblastoma samples together with normal liver (NL) samples as controls. We compared the expression profiles among the four clusters (the hepatoblastoma clusters F, E1, and E2 and NL; Fig. 3). The Wnt signaling and cell cycle pathways were commonly upregulated in the three hepatoblastoma clusters compared with the NL, whereas liver-associated pathways such as retinol metabolism and the cytochrome P450 pathway were commonly downregulated in the hepatoblastoma clusters (Supplementary Tables 6–11). On the other hand, comparison among the hepatoblastoma clusters indicated that clusters E1 and E2 had few differentially regulated pathways (Supplementary Tables 12 and 13), whereas cluster F was distinct from clusters E1 and E2. Specifically, the upregulation of the cell cycle pathway was more pronounced in clusters E1 and E2, while liver-associated pathways were relatively upregulated in cluster F (Supplementary Tables 14–17). These results suggested similarities between clusters E1 and E2 and a relative proximity of cluster F to the NL.

Considering the apparent contrast of cluster F with clusters E1/E2, we focused on the comparison of clusters F versus E1/E2 and analyzed the correlation between DNA methylation and expression. We performed region set enrichment analysis to test differentially methylated CpGs between cluster F and clusters E1/E2 for enrichment against sequence databases (Supplementary Tables 18 and 19 and Supplementary Data S1 and S2). The regions with the most significant overlap with the differentially hypermethylated CpGs in clusters E1/E2 were chromatin immunoprecipitation sequencing peaks in the NL cells determined by antibodies to HNF4A and CEBPA, which are essential transcription factors for hepatocyte differentiation^18^. In other words, a considerable part of the HNF4A/CEBPA-binding regions was differentially hypermethylated in clusters E1/E2. The overlapping CpG probes were mostly in gene bodies rather than promoters, and the differential methylation was strikingly similar to that found between normal adult and fetal livers: the same regions were clearly hypermethylated in the normal fetal liver compared with the normal adult liver (Supplementary Fig. 8). Furthermore, the expression profiles of clusters E1/E2 shared more similarities with fetal liver compared with cluster F whose expression pattern resembled that of the adult liver (Supplementary Fig. 9). These methylation and expression patterns are compatible because the high methylation level of the HNF4A/CEBPA-binding regions in clusters E1/E2 can prevent the binding of transcription factors, block differentiation, and render the expression pattern resemble that of the immature liver. Accordingly, these results suggest that the differential methylation of HNF4A/CEBPA-binding regions can be responsible for the diversity in tumor differentiation in hepatoblastoma.

### Novel therapeutic targets of high-risk hepatoblastoma

**Fig. 4:**
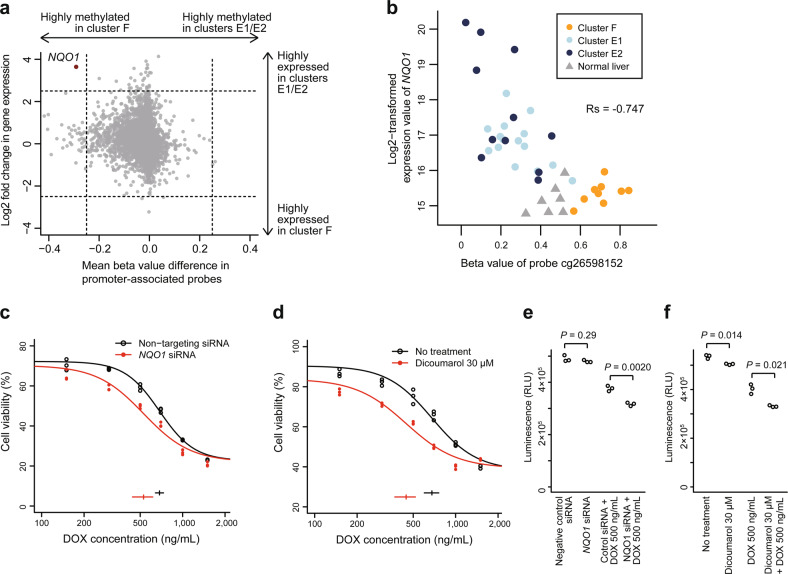
High expression of *NQO1* based on promoter hypomethylation is a characteristic of high-risk hepatoblastoma and a potential therapeutic target for chemoresistance. **a** Starburst plot showing the correlation of differences in promoter methylation and expression between the hepatoblastoma clusters F versus E1/E2. The only gene with absolute methylation difference ≥ 0.25 and absolute log2-fold expression change ≥ 2.5 is *NQO1*, indicated in red. **b** Correlation between the methylation of probe cg26598152 and *NQO1* expression. Rs represents Spearman’s correlation coefficient. **c**, **d** Dose–response curves of HepG2 cells exposed to various concentrations of doxorubicin (DOX) after *NQO1* inhibition (red) or negative control treatment (black). *NQO1* was inhibited by using siRNA (**c**) or dicoumarol (**d**). Horizontal bars and whiskers at the bottom indicate EC_50_ values with 95% confidence intervals. **e**, **f** Enhancement of DOX cytotoxicity by *NQO1* inhibition in HepG2 cells. *NQO1* was inhibited using siRNA (**e**) or dicoumarol (**f**). The luminescence intensities representing the cell viability are compared between the conditions with and without *NQO1* inhibition using the unpaired Student’s *t* test.

To further assess the effect of differential methylation on expression, we integrated the gene-level differential methylation and expression analyses between clusters F versus E1/E2 (Fig. 4a). The most differentially overexpressed gene with promoter hypomethylation in clusters E1/E2 was *NQO1*. The differential methylation status of the *NQO1* promoter between cluster F and clusters E1/E2 was very similar to that observed between normal adult and fetal livers (Supplementary Fig. 10a), which suggested that tumor differentiation highly affected *NQO1* promoter methylation in hepatoblastoma. Among the CpG probes associated with *NQO1*, cg26598152, the nearest probe to the *NQO1*-antioxidant response element (ARE), a *cis*-acting enhancer of *NQO1*^19^, was the probe whose methylation was the most negatively correlated with *NQO1* expression (Fig. 4b and Supplementary Fig. 10b–e). It suggests the possibility that *NQO1* expression in hepatoblastoma is highly regulated by the *NQO1*-ARE.

NQO1 is a well-known antioxidant/detoxifying enzyme and functions in reduction/detoxification of quinones^20^. High expression of *NQO1* is known to be a poor prognostic factor in several types of cancers, including hepatoblastoma^8,21^, and *NQO1* inhibition has been reported to sensitize multiple types of cancers to anticancer drugs^22,23^. Given that quinone-containing anthracyclines play an important role in hepatoblastoma treatment, we hypothesized that high *NQO1* expression in hepatoblastoma clusters E1/E2 contributed to chemoresistance and poor outcome. To assess the synergistic effect of *NQO1* inhibition and doxorubicin on *NQO1*-high hepatoblastoma cell lines, we performed a drug sensitivity assay, which revealed that both *NQO1* siRNA and dicoumarol, an NQO1 inhibitor, significantly lowered the EC_50_ values of doxorubicin (Fig. 4c, d and Supplementary Fig. 11a, b). The combination of *NQO1* inhibition and doxorubicin significantly reduced cell viability compared with doxorubicin alone (Fig. 4e, f and Supplementary Fig. 11c, d), indicating that increased *NQO1* expression was a key event in anthracycline resistance.

**Fig. 5:**
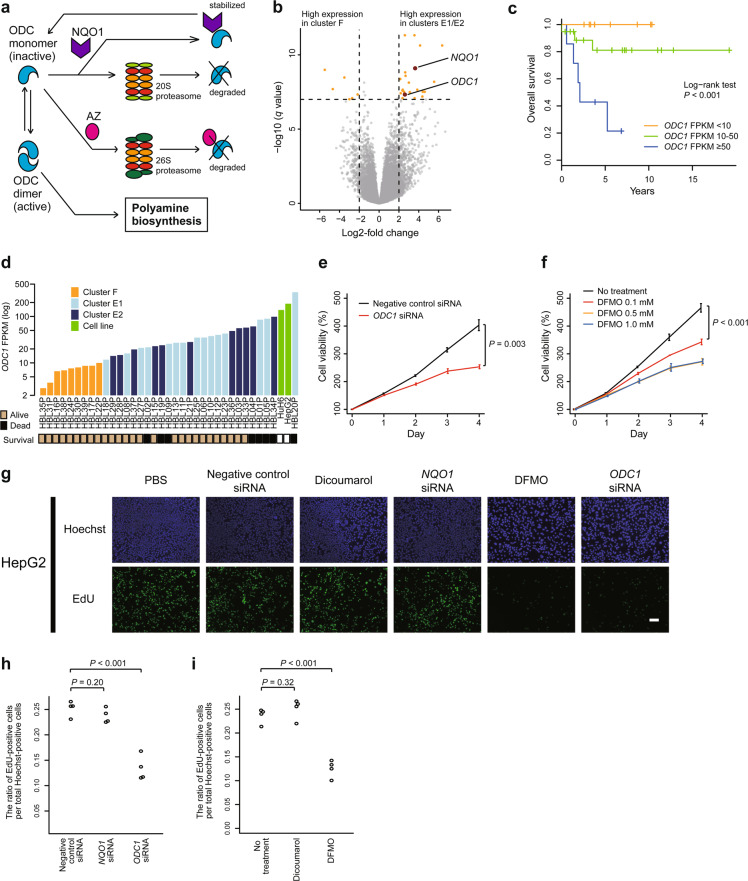
*ODC1* is differentially overexpressed in high-risk hepatoblastoma and a key molecule for rapid cell proliferation in hepatoblastoma. **a** Schematic presentation of ODC1 stabilization by NQO1. **b** Volcano plot displaying genes that are differentially expressed between the hepatoblastoma clusters F versus E1/E2. Each gene is plotted with log2-fold expression change on *x*-axis and negative log10 false discovery rate (FDR) *q* value on *y*-axis. Genes with absolute log2-fold change > 2 and an FDR *q* value of <1.0 × 10^−7^ are shown in orange. *NQO1* and *ODC1* are shown in red. **c** Kaplan–Meier survival curves for overall survival according to *ODC1* expression. **d** *ODC1* FPKM in hepatoblastoma samples and cell lines. **e**, **f** Cell proliferation assay to assess the effect of *ODC1* inhibition on HepG2 cells. *ODC1* was inhibited using siRNA (**e**) or difluoromethylornithine (DFMO; **f**). Error bars indicate SD of triplicate experiments. Cell viabilities on day 4 are compared between the conditions using the unpaired Student’s *t* test. **g**–**i** Ethynyl deoxyuridine (EdU) assay using HepG2 cells treated with PBS, dicoumarol, DFMO, and negative control/*NQO1*/*ODC1* siRNA. The ratio of EdU-positive cells per total Hoechst-positive cells are compared among the conditions using the unpaired Student’s *t* test. Scale bar represents 100 μm.

To further explore new therapeutic targets, we focused on a slight but significant growth arrest of hepatoblastoma cell lines after *NQO1* inhibition alone, which was observed in the abovementioned experiments (Fig. 4f and Supplementary Fig. 11c, d). A function of NQO1 other than antioxidant activity is stabilizing ODC1, a key enzyme for polyamine formation and cell proliferation (Fig. 5a)^24^. In fact, not only was the ODC1 protein level decreased in NQO1-low hepatoblastoma samples compared with NQO1-high samples, despite the comparable high ODC1 mRNA expression (Supplementary Fig. 12a–c), but we also observed a reduction in ODC1 protein levels in HepG2 cells after NQO1 inhibition (Supplementary Fig. 12d, e); these results indicate that NQO1 plays an important role in the stabilization of the ODC1 protein in hepatoblastoma cells. Thus, we considered that *NQO1* inhibition-associated growth arrest was due to ODC1 instability. Moreover, *ODC1* was among the most differentially upregulated genes in high-risk hepatoblastoma cases (Fig. 5b–d). Altogether, we hypothesized that *ODC1* was a key molecule for aggressive cell proliferation and a candidate therapeutic target in high-risk hepatoblastoma. Our cell proliferation assay to investigate whether *ODC1* inhibition suppressed cell growth in *ODC1*-high hepatoblastoma cell lines revealed that both *ODC1* siRNA and difluoromethylornithine (DFMO), an ODC1 inhibitor, significantly inhibited cell proliferation (Fig. 5e, f and Supplementary Fig. 13). We also performed apoptosis and cell cycle assays to determine the cause of the decrease in cell viability observed after ODC1 inhibition. We found that this inhibition in cell proliferation was associated with cell cycle arrest, rather than apoptosis (Fig. 5g–i and Supplementary Fig. 14).

### Genetic differences between clusters E1 and E2

Finally, we examined the differences in the DNA methylation and expression profiles between the genetically similar clusters E1 and E2. The most significant differential methylation between the two clusters was observed in the gene body of *STAP2* and the promoter-associated region of *C1orf51/CIART* (Supplementary Fig. 15a, b and Supplementary Data S3). However, these methylation differences did not alter the expression of *STAP2* or *CIART* (Supplementary Fig. 15c, d). Hence, the biological significance of the differential methylation was unclear.

Differential expression analysis revealed significantly higher expression of *HBG1*, *HBG2*, *TMCC2*, *CLMP*, *ALAS2*, and *HBM* in cluster E1 (Supplementary Fig. 16a). Differential expression of these genes except *CLMP* was due to the outliers in cluster E1 (HBL05P, HBL06P, and HBL09P), which showed extremely high expression of these genes (Supplementary Fig. 16b–g). Among these genes, *HBG1* and *HBG2* encode hemoglobin gamma chains, whereas *ALAS2* encodes the erythroid-specific delta-aminolevulinate synthase, all of which are associated with hematopoiesis in the fetal liver^25,26^. In addition, HBL05P, HBL06P, and HBL09P were some of the youngest cases in the study cohort who were diagnosed with hepatoblastoma within the first 5 months of life. Taken together, high expression of abovementioned genes was presumed to reflect the immaturity of the tumor in some cases in cluster E1. In fact, in the expression analysis shown in the section above (Supplementary Fig. 9g), HBL05P, HBL06P, and HBL09P exhibited the highest mean expression of the 250 genes that were differentially highly expressed in immature fetal liver at 10.5 weeks of gestation (Supplementary Fig. 16h). On the contrary, differential expression analysis also revealed significantly higher expression of *CCL25*, *DUSP2*, *KLRK1*, and *NQO1* in cluster E2 (Supplementary Fig. 16a). Among these genes, high expression of *NQO1* may contribute to stronger chemoresistance and higher need for liver transplantation in cluster E2 (Fig. 2a and Supplementary Fig. 16i). Meanwhile, differential high expression of the other genes in cluster E2 was due to one outlier sample (Supplementary Fig. 15j–l), therefore, the biological significance was unclear.

## Discussion

In this study, we present a genome-wide molecular portrait of hepatoblastoma characterized by uniformly upregulated Wnt signaling pathway and novel DNA methylation clusters which tightly correlate with genetic abnormalities, histological subtypes, and clinical behaviors. The landscape of gene mutations and CN alterations revealed by the Target-seq and SNP array analyses revealed a high prevalence of Wnt-activating mutations, whole-arm CN gains, and 11p uniparental disomy/trisomy, which is consistent with previous reports^7–9,17^. Additional recurrent gene mutations were observed in *DST*, *PEG10*, *PTPRO*, and the *TERT* promoter. Although some of these genes have been reported to be related to the Wnt signaling pathway^27–29^, we did not find a significant difference in Wnt activation levels between samples with and without those gene mutations (Supplementary Fig. 17). Thus, it remains unclear whether these mutations have synergistic effects on aberrant Wnt activation in hepatoblastoma.

**Fig. 6:**
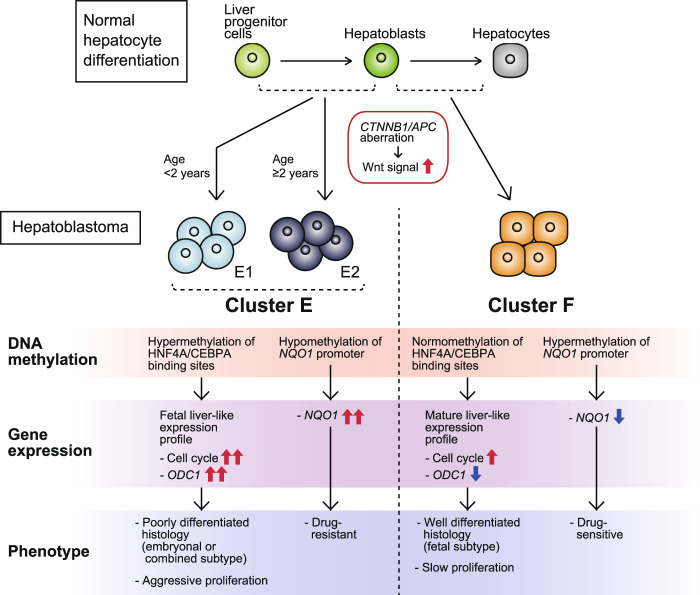
The molecular model of hepatoblastoma tumorigenesis and genetic/clinical heterogeneity. All hepatoblastoma cells are commonly derived from immature hepatocytes with aberrant activation of the Wnt signaling pathway, whereas heterogeneity among cases arises from the diversity of the differentiation stage of the origins. Clusters E1/E2 are derived from liver progenitor cells at an earlier differentiation stage and consequently harbor hypermethylation of HNF4A/CEBPA-binding regions that leads to expression profiles mimicking fetal liver, which explain the poorly differentiated pathology and aggressive cell proliferation. In addition, clusters E1/E2 highly express *NQO1* due to promoter hypomethylation, which induces chemoresistance. Cluster F arises from hepatoblasts at a relatively mature stage, harbors genetic features that are opposite of those observed in clusters E1/E2, and represents good prognosis.

To elucidate the heterogeneity of hepatoblastoma, which was not fully explained by the genomic landscape described above, we analyzed DNA methylome and transcriptome data and successfully unraveled the genetic heterogeneity of this disease by identifying the novel methylation clusters F, E1, and E2. The current results propose a model of hepatoblastoma tumorigenesis and heterogeneity (Fig. 6). In this model, poor prognostic clusters E1/E2 originate from liver progenitor cells at a more immature stage, which consequently harbor hypermethylation of the HNF4A/CEBPA-binding regions and gene expression profiles that resemble those of fetal liver as a result. Through upregulation of the cell cycle pathway and overexpression of *NQO1* and *ODC1*, they exhibit an aggressive and chemoresistant tumor phenotype as well as a poorly differentiated histology. Conversely, Cluster F arises from hepatoblasts at a relatively mature stage, harboring genetic and clinical features that are opposite to those of clusters E1/E2.

Several past studies have reported that tumor differentiation and pathology have a great impact not only on the biology of hepatoblastoma, but also on its clinical features^7,10,11,30^. In this study, we explained a large proportion of the genetic heterogeneity of hepatoblastoma by comparing the methylation clusters F and E1/E2. However, this comparison largely reflects the contrast between fetal and non-fetal histologies, and fairly overlaps with the C1/C2 classification previously described by Cairo et al. (Supplementary Fig. 18a, b). In this sense, we cannot claim that the methylation clusters have a large additive value regarding prognostic prediction. Rather, one of the advantages of the current work is that the cell origin of hepatoblastoma is placed on a firmer basis by analyzing comprehensive methylome data.

In addition, the separation of cluster E into two subgroups E1 and E2 by age of diagnosis is presumably meaningful, given the importance of age as a prognostic factor in hepatoblastoma^3^. This classification may provide a clue about the molecular mechanism of aggressive hepatoblastoma that develops in older patients. In this study, however, we are yet to point out the clear genetic difference between clusters E1 and E2, except that more frequent CN gains of chromosomes 1q and 2 and higher expression of *NQO1* are observed in cluster E2, whereas some cases in cluster E1 exhibited very immature expression profiles. Although the methylation cluster E fairly overlaps with the C2 group described by Cairo et al.^7^, the subclassification of cluster E into E1 and E2 was very different from that of C2, which was proposed by Hooks et al. and uses a four-gene signature to provide two subgroups, C2A and C2B^11^ (Supplementary Fig. 18c). Thus, the genetic differences between the cases in high and low age groups are still unclear and should be further explored in future studies.

Another valuable finding of the current study is the identification of novel therapeutic targets, *NQO1* and *ODC1*, in high-risk hepatoblastoma. Although high expression of *NQO1* has been reported as a poor prognostic factor in hepatoblastoma in a previous study^8^, the mechanism of high *NQO1* expression has not been clarified except for activating mutation of *NEF2L2*, a transcription factor upstream of *NQO1*. The current study pointed out the possibility that methylation of *NQO1*-ARE highly regulated *NQO1* expression. In addition, we confirmed the druggability of *NQO1* and *ODC1* by in vitro experiments using multiple inhibition methods and multiple cell lines. Of note, DFMO (*ODC1* inhibitor) was effective in as low concentration as 0.1–0.5 mM, which was lower than used in previous experiments performed on neuroblastoma cell lines^31^. Given that DFMO has been clinically adopted as a therapeutic choice in refractory neuroblastoma^32^, DFMO can also be an option in the treatment of *ODC1*-high human hepatoblastoma. Of course, forced expression assays or in vivo experiments are necessary to further evaluate the roles of *NQO1* and *ODC1* in cell proliferation and chemoresistance.

In summary, the current results propose a DNA methylation-based classification that explains the genetic and clinical diversity of hepatoblastoma and shed light on the high expression of *NQO1* and *ODC1*, potential druggable targets, in high-risk hepatoblastoma.

## Methods

### Patients and samples

A total of 59 fresh-frozen tumor samples, 7 NL samples, and 15 normal blood samples were collected from 60 hepatoblastoma patients, after written informed consent was obtained according to protocols approved by the Human Genome, Gene Analysis Research Ethics Committee of the University of Tokyo and other participating institutions. The 59 tumor samples comprised 39 samples collected at primary diagnostic biopsy (HBL01P–HBL39P) and 20 samples collected at postchemotherapy resection (HBL40C–HBL59C). All hepatoblastoma samples were pathologically reviewed by three expert pathologists to confirm the diagnosis and presence of tumor. Genomic DNA and total RNA were isolated from all the collected samples and the hepatoblastoma cell lines HepG2 and HuH6 for massive parallel sequencing and microarray analysis. In addition, adult human liver genomic DNA and total RNA obtained from the commercial sources BioChain and ZYAGEN were also analyzed. The list of the samples that underwent comprehensive genetic analysis is provided in Supplementary Table 1. Information on the clinical characteristics of the 59 hepatoblastoma cases was collected from the medical records and is shown in Supplementary Table 2. Regarding treatment, therapeutic strategies were not completely uniform but were comparable among all cases: the patients were stratified based on the clinical information including pathology, PRETEXT, metastasis, and serum alpha-fetoprotein levels and were treated with surgical resection and/or adjuvant chemotherapy. The chemotherapy regimens were based on the JPLT^33,34^ or SIOPEL^2,35–37^ protocols that employed cisplatin and anthracyclines.

### Statistics

Statistical analyses were performed using the R software version 3.5.1 (https://www.R-project.org/).

### Targeted capture sequencing

DNA isolated from 59 hepatoblastoma tumor samples and two hepatoblastoma cell lines were analyzed by Target-seq. Sequencing libraries were constructed using a SureSelect XT custom kit (Agilent Technologies) according to the manufacturer’s protocol. Massive parallel sequencing of the library was performed using the HiSeq 2000/2500 platform (Illumina) with a 126-bp paired-end read protocol according to the manufacturer’s instructions. The custom gene panel was designed for mutation profiling of pediatric cancers, including (i) all coding exons of 366 cancer-associated genes (Supplementary Table 20), (ii) untranslated regions and introns of 16 genes (*CD274, CTNNB1, ERG, ETV1, ETV4, EWSR1, FEV, FLI1, FOXO1, FUS, INO80D, NCOA1, NCOA2, NOTCH1, PAX3*, and *PAX7*) for detecting breakpoints of structural variations, (iii) 110,000 bases surrounding *TERT* for detecting *TERT* rearrangement and promoter/enhancer mutation, (iv) promoter and enhancer regions of *FGFR3* and *MYC*, and (v) 11 microRNA genes (*MIR100, MIRLET7A1, MIRLET7A2, MIRLET7A3, MIRLET7B, MIRLET7C, MIRLET7D, MIRLET7E, MIRLET7F1, MIRLET7F2, MIRLET7G*). Sequence alignment and detection of gene mutations and structural variations were performed using our in-house pipeline, Genomon v.2.5.3 (https://github.com/Genomon-Project/).

### Mutation and structural variation analysis

Gene mutations called by the Genomon pipeline were first filtered to exclude sequencing/mapping errors and mutations of unknown significance, using the following parameters: (i) mapping quality score ≥ 20, (ii) base quality score ≥ 15, (iii) nonsilent exonic/splice-site mutations, (iv) strand ratio not equal to 0/1, (v) read depth ≥ 100, (vi) number of both reference and variant read pairs ≥ 5, (vii) variant allele frequency (VAF) ≥ 0.05, and (viii) EBCall^38^ *P* value < 10^−20^. Next, candidate mutations were further filtered to exclude mutations likely to be germline SNPs using the following procedures: (i) removal of variants listed in SNP databases, (ii) exclusion of mutations with a VAF ≥ 0.35 in copy-neutral regions without loss of heterozygosity, (iii) exclusion of mutations with a VAF ≥ 0.25 in CN-gained regions without loss of heterozygosity, and (iv) reinclusion of mutations with ten or more mentions of solid tumors in the Catalogue of Somatic Mutations In Cancer Database version 68 on WGS data and version 70. In addition, mapping errors were removed by visual inspection on the Integrative Genomics Viewer browser. Validity of the filtering process in distinguishing somatic and germline mutations was confirmed by Sanger sequencing in 17 cases where matched germline and tumor DNA samples were both available (HBL01, HBL02, HBL03, HBL04, HBL08, HBL12, HBL16, HBL24, HBL25, HBL30, HBL31, HBL33, HBL35, HBL37. HBL41, HBL43, and HBL45) and determining if each mutation was germline or somatic. In total, 19 of the 21 mutations filtered as “somatic” were truly somatic, and all 173 mutations filtered as “germline” were truly germline. On the ground of the high positive and negative predictive values (91% and 100%, respectively), we applied the filtering method to the other cases and fixed the list of somatic exonic/splice-site mutations. In addition, germline truncating mutations of *APC* were picked up from the list of germline mutations, considering its importance in hepatoblastoma tumorigenesis^15,16^. Finally, a distinctive filtering method was adopted for detecting *TERT* promoter mutations because the filtering procedure shown above missed all *TERT* promoter mutations due to low read depth of ~10 around the promoter region. The filtering conditions were as follows: chromosomal position within chr5:1295105–1295353 and an EBCall *P* value < 10^−4^. In addition detected *TERT* promoter mutations were combined with the mutation list above to create the final list of gene mutations (Supplementary Table 3).

Structural variations called by the Genomon pipeline were filtered with the following parameters: (i) number of reference read pairs ≥ 300, (ii) number of valiant read pairs ≥ 20, and (iii) maximum overhang ≥ 150 bps for both sides of the breakpoint. Finally, the following structural variations were removed to create the final list: (i) structural variations whose breakpoints were mapped on mitochondrial/linear DNA and (ii) deletions and tandem duplications within an intron appearing not to affect coding exons (Supplementary Table 4).

### SNP array analysis

A total of 59 hepatoblastoma samples and two hepatoblastoma cell lines were analyzed by SNP array using the Human Mapping 250K Nsp Array for Cytogenetics (Affymetrix) according to the manufacturer’s protocol. The array data were analyzed for CN alterations and allelic imbalances using the CNAG software version 3.5.1^39,40^.

### DNA methylation array analysis

Genomic DNA extracted from 59 hepatoblastoma samples, nine NL samples, and two hepatoblastoma cell lines were treated with bisulfite and analyzed by DNA methylation array using Infinium MethylationEPIC BeadChip (Illumina) according to the manufacturer’s protocol. Quality control, signal correction, calculation of methylation beta value, data normalization, and differential methylation analysis were performed using Bioconductor package ChAMP version 2.10.1^41^. The differentially methylated CpG probes were ranked by adjusted *P* values that were calculated by fitting linear models, and top-ranked probes are shown in tables (Supplementary Data S1–S3).

### Consensus clustering of methylation data

To unravel the heterogeneity of hepatoblastoma, consensus clustering of methylation data was performed using Bioconductor package ConsensusClusterPlus version 1.44.0^42^. First, consensus clustering of 59 hepatoblastoma samples was performed (Supplementary Fig. 4). To select the most variably methylated CpG probes among the samples, standard deviations (SD) of the methylation beta values of the promoter-associated CpG probes (annotated as “Promoter_Associated” or “Promoter_Associated_Cell_type_specific” in the manifest file supplied by the manufacturer and designed in “Island,” “N_Shore,” or “S_Shore” regions on autosomes) were calculated. Top 100, 1000, 2000, 3000, and 10,000 probes ranked by SD were selected (Supplementary Data S4), and consensus clustering of the 59 samples within the space of the selected probes with Euclidean or Pearson correlation metrics was performed with 1000 iterations (Supplementary Fig. 5a–j). From the cumulative distribution function plots, the most robust clustering was obtained using the top 2000 probes and Pearson correlation metrics (Supplementary Fig. 5h, k). According to the clustering, a methylation heatmap was constructed for the 2000 probes across the 59 samples, with addition of the clinical information (Supplementary Fig. 5l).

Next, consensus clustering of 39 hepatoblastoma biopsy samples was performed. The most variably methylated CpG probes were selected using the approach for the analysis of the 59 samples described above. For the first-step consensus clustering of 39 biopsy samples, top 3000 probes ranked by SD were selected (Supplementary Data S5). Consensus clustering of the 39 samples within the space of the 3000 probes with Euclidean metrics and 1000 iterations generated two robust clusters termed F and E (Supplementary Fig. 6a–c). For the second-step consensus clustering of 30 cluster E samples, top 1000 CpG probes ranked by SD were selected (Supplementary Data S6). Consensus clustering of the 30 samples within the space of the 1000 probes with Pearson correlation metrics and 1000 iterations generated two robust clusters termed E1 and E2 (Supplementary Fig. 6d–f).

### Enrichment analysis of differentially methylated regions

Region set enrichment analysis of differentially methylated CpGs between hepatoblastoma clusters were performed using Bioconductor package LOLA version 1.6.0^43^. The top 2000 differentially methylated CpG probes between cluster F and clusters E1/E2 (Supplementary Data S1 and S2) were tested for enrichment against the LOLA core sequence database (Supplementary Data S7). The top 50 regions ranked by the false discovery rate *q* value are listed in tables (Supplementary Tables 18 and 19).

### RNA sequencing

Among 59 hepatoblastoma samples, RNA with adequate quality for RNA-seq based on an RNA integrity number equivalent score of ≥5.0 determined by a 4200 TapeStation system (Agilent Technologies) was available for 50 samples. In addition to the 50 hepatoblastoma samples (35 biopsy samples and 15 postchemotherapy samples), ten NL samples and two HBL cell lines were assessed by RNA-seq (Supplementary Table 1). Sequencing libraries were constructed using a NEBNext Ultra RNA Library Prep Kit for Illumina (New England Biolabs) according to the manufacturer’s protocol. Massive parallel sequencing of the library was performed using the HiSeq 2000/2500 platform with a 100-bp/126-bp paired-end read protocol according to the manufacturer’s instructions. Sequence alignment and read counting were performed using the Genomon pipeline.

### Gene expression analysis

Normalization of the read counts of RNA-seq data and differential expression analysis were performed using Bioconductor package DESeq2 version 1.20.0^44^. Differentially expressed genes among four clusters (NL and the three hepatoblastoma clusters F, E1, and E2) were ranked by adjusted *P* values that were determined by the likelihood ratio test for significance of the change in deviance between a full and reduced model. Top 2000 differentially expressed genes are shown in Supplementary Data S8. Pathway analysis of differential expression among the four clusters was performed by using generally applicable gene set enrichment (GAGE) method implemented in Bioconductor package gage version 2.30.0^45^. Differentially regulated KEGG pathways^46^ with a false discovery rate *q* value of <0.001 are listed in tables (Supplementary Tables 6–17). Principal component analysis was performed for the most variably expressed 10,000 genes ranked by median absolute deviation of the log-transformed expression values among the 35 hepatoblastoma and ten NL samples (Fig. 3b).

### DNA methylation analysis of normal adult and fetal livers

DNA methylation data of the normal adult and fetal livers generated on Infinium HumanMethylation450K BeadChip (Illumina) were obtained from the Gene Expression Omnibus (GEO) under the accession number GSE61278^47^ and processed using ChAMP.

### Expression analysis of normal adult and fetal livers

RNA-seq data of the normal adult and fetal livers were downloaded from the GEO under the accession number GSE96981^48^ and processed using the Genomon pipeline and DESeq2. Differential expression analysis among the three groups (adult liver, fetal liver at 17.5 weeks of gestation, and fetal liver at 10.5 weeks of gestation) was performed by the likelihood ratio test implemented in DESeq2 (Supplementary Data S9).

### Integration of DNA methylation and expression analyses

DNA methylation of the promoter and expression of each gene were compared between hepatoblastoma clusters F and clusters E1/E2. First, the difference in promoter methylation for each gene was calculated as follows. (i) CpG probes that were associated with the gene and annotated as “Promoter_Associated” or “Promoter_Associated_Cell_type_specific” in the manifest file were selected. (ii) Mean methylation beta values of all the selected probes were calculated for clusters F and E1/E2. (iii) The difference between the two values was adopted. Then, log2-fold change in the expression of each gene was calculated using DESeq2. Each gene was plotted with promoter methylation difference on *x*-axis and expression change on *y*-axis (Fig. 4a).

### Correction of *NQO1* expression by the polymorphism C609T

Based on previous studies reporting that the *NQO1* C609T polymorphism (rs1800566) highly affected its enzymatic activity and that the T/T genotype harbored only 2–4% NQO1 activity compared with the wild-type C/C genotype^49,50^, corrected *NQO1* expression value (FPKM, fragments per kilobase of transcript per million mapped reads) in each sample was calculated as follows:

1 $${\mathrm{Corrected}}\,NQO1\,{\mathrm{FPKM}} = {\mathrm{raw}}\,NQO1\,{\mathrm{FPKM}} \,\times \frac{{{\mathrm{Nc}} + 0.03 \times {\mathrm{Nt}}}}{{{\mathrm{Nc}} + {\mathrm{Nt}}}},$$

where Nc and Nt represented read counts with C and T alleles at SNP rs1800566 in the RNA-seq data, respectively.

### Survival analysis

Overall survival was measured from the date of diagnosis to the date of death from any cause or last follow-up, whereas event-free survival was measured from the date of diagnosis until the date of the first event (relapse, failure to achieve remission, second malignancy, or death from any cause) or last follow-up. Failure to achieve remission was evaluated as an event on day 0. The Kaplan–Meier method was used to generate survival curves for each subgroup, and the log-rank test was used to test differences between the curves.

### Cell lines

The hepatoblastoma cell lines HepG2 (RCB1886) and HuH6 (RCB1367) were obtained from RIKEN BRC Cell Bank (Tsukuba, Japan). Both cell lines were cultured in high-glucose Dulbecco’s Modified Eagle Medium (Sigma Aldrich) supplemented with 10% fetal bovine serum (Gibco, Invitrogen) at 37 °C in a humidified incubator with 5% CO_2_.

### Chemosensitivity assay after *NQO1* knockdown by siRNA

Approximately 5000 HepG2 or HuH6 cells were plated in each well of 96-well plates. After 24 h of incubation, *NQO1* siRNA (s4089/s4091) or negative control siRNA (#4390843), both from Applied Biosystems, was transfected using the Lipofectamine RNAiMAX reagent (Life Technologies) with minor modifications from the manufacturer’s protocol: the amount of siRNA and Lipofectamine reagent added in each well was reduced by 30% from the recommended values to avoid cytotoxicity. The efficacy of the modified method was comparable with that of the original method, which was confirmed by RT-PCR. After siRNA transfection, the cells were incubated for 24 h, and doxorubicin (Cayman Chemical) was added in various concentrations (0–5000 ng/mL). After an additional 48 h of incubation, cell viability was measured by an ATP assay using the CellTiter-Glo reagent (Promega) following the manufacturer’s instructions. The experiment was performed in triplicate and repeated three times with equivalent results.

### Chemosensitivity assay after NQO1 inhibition by dicoumarol

Approximately 5000 HepG2 or HuH6 cells were plated in each well of 96-well plates. After 24 h of incubation, the NQO1 inhibitor dicoumarol (Tokyo Kasei Kogyo) or its solvent as a negative control was added. The concentration of dicoumarol was 30 μM. After 24 h of incubation, doxorubicin was added in various concentrations (0–5000 ng/mL). After an additional 48 h of incubation, cell viability was measured by an ATP assay using the CellTiter-Glo reagent. The experiment was performed in triplicate and repeated three times with equivalent results.

### NQO1 and ODC1 immunohistochemistry assay

NQO1 and ODC1 immunostaining was performed on formalin-fixed paraffin-embedded tumor tissue sections using antibodies directed against NQO1 (11451-1-AP; Proteintech) and ODC1 (17003-1-AP; Proteintech) at the concentrations of 1/150 and 1/200, respectively.

### Western blot analysis

HepG2 cells were plated on 6-cm dishes at a density of ~500,000 cells/dish. The cells were transfected with *NQO1* or negative control siRNA at 24 h after plating. After an additional 36 h of incubation, the cells were collected and lysed. Another set of cells plated in parallel were treated with dicoumarol or its solvent as a negative control at 48 h after plating and lysed after an additional 12 h of incubation. Whole-cell lysates were analyzed by western blotting using antibodies against alpha-tubulin (ab7291; Abcam), NQO1 (NB200-209; Novus Biologicals), and ODC1 (GTX101521; GeneTex) at the concentrations of 1/10,000, 1/1666, and 1/1000, respectively. Normalized ODC1 band intensity was calculated by dividing the ODC1 band volume in each condition by the corresponding band volume of alpha-tubulin. The experiment was performed in triplicate and repeated twice with equivalent results.

### Cell proliferation assay after *ODC1* knockdown by siRNA

Approximately 5000 HepG2 or HuH6 cells were plated in each well of 96-well plates. After 24 h of incubation, *ODC1* siRNA (s9821) or negative control siRNA (#4390843) from Applied Biosystems was transfected using the Lipofectamine RNAiMAX reagent with minor modifications from the manufacturer’s protocol, wherein the amount of siRNA and Lipofectamine reagent added in each well was reduced by 30% from the recommended volume to avoid cytotoxicity. The efficacy of the modified method was comparable with that of the original method, which was confirmed by RT-PCR. Cell viability was measured by the ATP assay using the CellTiter-Glo reagent at 0, 24, 48, 72, and 96 h of incubation after the transfection. The experiment was performed in triplicate and repeated three times with equivalent results.

### Cell proliferation assay after ODC1 inhibition by DFMO

Approximately 5000 HepG2 or HuH6 cells were plated in each well of 96-well plates. After 24 h of incubation, the ODC1 inhibitor DFMO (Tokyo Kasei Kogyo) or its solvent as a negative control was added. The DMFO concentrations were 0.1, 0.5, and 1.0 mM. Cell viability was measured by the ATP assay using CellTiter-Glo reagent at 0, 24, 48, 72, and 96 h of incubation after the DFMO treatment. The experiment was performed in triplicate and repeated three times with equivalent results.

### Apoptosis assay

Approximately 5000 HepG2 or HuH6 cells were plated in each well of 96-well plates. After 24 h of incubation, the cells were treated with PBS as a negative control, 1000 ng/mL of doxorubicin as a positive control, 30 μM dicoumarol, and 1.0 mM DFMO. Apoptosis signals were measured at 0–48 h after the treatment using the RealTime-Glo Annexin V Apoptosis Assay Reagent (Promega), according to the manufacturer’s instructions. The experiment was performed in hexaplicate and repeated twice with equivalent results.

### Cell cycle assay

Approximately 5000 HepG2 or HuH6 cells were plated in each well of 96-well plates. After 24 h of incubation, the cells were treated with PBS, 30 μM dicoumarol, 5.0 mM DFMO, and a negative control/*NQO1*/*ODC1* siRNA. After 96 h of treatment, the cells were exposed to 10 μM ethynyl deoxyuridine (EdU) for 2 h and stained with 488-azide (for the detection of EdU) and Hoechst-33342 using a Click-iT EdU Alexa Fluor 488 imaging kit (Thermo Fisher Scientific), according to the manufacturer’s instructions. EdU-positive and total cell counts were obtained using the ImageJ software version 1.52a (https://imagej.nih.gov/ij/). The experiment was performed in quadruplicate and repeated twice with equivalent results.

### Reporting summary

Further information on research design is available in the Nature Research Reporting Summary linked to this article.

## Supplementary information

Supplementary Data

Supplementary Information

Reporting Summary

## Data Availability

Target-seq, RNA-seq, SNP array, and DNA methylation array data obtained in the current study were deposited in the Japanese Genotype-phenotype Archive^51^ under the accession number JGAS00000000188.
