# Supplementary material for: Integrated multiomics analysis of hepatoblastoma unravels its heterogeneity and provides novel druggable targets
Source: NPJ Precis Oncol. 2020 Jul 7;4:20. doi: 10.1038/s41698-020-0125-y (PMC7341754; doi:10.1038/s41698-020-0125-y)
Supplement: Supplementary file 2 — Supplementary Information [file 41698_2020_125_MOESM2_ESM.pdf]

Supplementary Information for

Integrated multiomics analysis of hepatoblastoma unravels its heterogeneity and provides novel druggable targets

Masahiro Sekiguchi[1], Masafumi Seki[1], Tomoko Kawai[2], Kenichi Yoshida[3], Misa Yoshida[1], Tomoya Isobe[1], Noriko Hoshino[4], Ryota Shirai[5], Mio Tanaka[6], Ryota Souzaki[7], Kentaro Watanabe[1], Yuki Arakawa[8], Yasuhito Nannya[3], Hiromichi Suzuki[3], Yoichi Fujii[9], Keisuke Kataoka[10], Yuichi Shiraishi[11], Kenichi Chiba[11], Hiroko Tanaka[12], Teppei Shimamura[13], Yusuke Sato[9], Aiko Sato-Otsubo[1], Shunsuke Kimura[1,14], Yasuo Kubota[1], Mitsuteru Hiwatari[1], Katsuyoshi Koh[8], Yasuhide Hayashi[15], Yutaka Kanamori[16], Mureo Kasahara[17], Kenichi Kohashi[18], Motohiro Kato[5], Takako Yoshioka[19], Kimikazu Matsumoto[5], Akira Oka[1], Tomoaki Taguchi[7], Masashi Sanada[20], Yukichi Tanaka[6], Satoru Miyano[11], Kenichiro Hata[2], Seishi Ogawa[3], and Junko Takita[1,21]*

[1]Department of Pediatrics, Graduate School of Medicine, The University of Tokyo, Tokyo, Japan. [2]Department of Maternal-Fetal Biology, National Research Institute for Child Health and Development, Tokyo, Japan. [3]Department of Pathology and Tumor Biology, Graduate School of Medicine, Kyoto University, Kyoto, Japan. [4]Department of Pediatric Surgery, The University of Tokyo Hospital, Tokyo, Japan. [5]Children's Cancer Center, National Center for Child Health and Development, Tokyo, Japan. [6]Department of Pathology, Kanagawa Children's Medical Center, Kanagawa, Japan. [7]Department of Pediatric Surgery, Faculty of Medical Sciences, Kyushu University, Fukuoka, Japan. [8]Department of Hematology/Oncology, Saitama Children's Medical Center, Saitama, Japan. [9]Department of Urology, Graduate School of Medicine, The University of Tokyo, Tokyo, Japan. [10]Division of Molecular Oncology, National Cancer Center Research Institute, Tokyo, japan. [11]Center for Cancer Genomics and Advanced Therapeutics, National Cancer Center Research Institute, Tokyo, Japan. [12]Laboratory of DNA Information Analysis, Human Genome Center, The Institute of Medical Science, The University of Tokyo, Tokyo, Japan. [13]Department of Systems Biology, Graduate School of Medicine, Nagoya University, Nagoya, Japan. [14]Department of Pediatrics, Hiroshima University Graduate School of Biomedical Sciences, Hiroshima, Japan. [15]Jobu University, Gunma, japan. [16]Division of Surgery, Department of Surgical Specialties, National Center for Child Health and Development, Tokyo, Japan. [17]Transplantation Center, National Center for Child Health and Development, Tokyo, Japan. [18]Department of Anatomic Pathology, Graduate School of Medical Sciences, Kyushu University, Fukuoka, Japan. [19]Department of Pathology, National Center for Child Health and Development, Tokyo, japan. [20]Department of Advanced Diagnosis, Clinical Research Center, Nagoya Medical Center, Nagoya, Japan. [21]Department of Pediatrics, Graduate School of Medicine, Kyoto University, Kyoto, Japan.

This file includes:

Supplementary Figures 1–18
Supplementary Tables 1–20

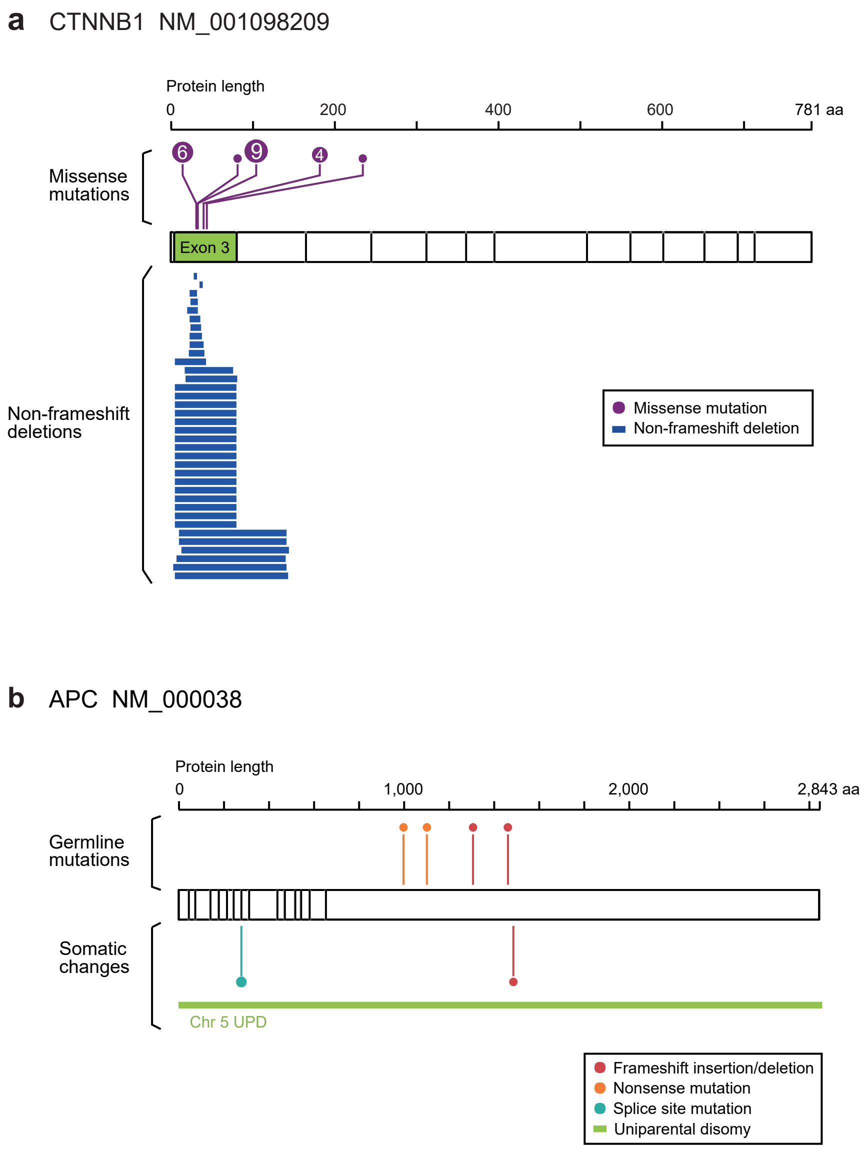

Supplementary Figure 1.

Distribution of driver gene mutations and structural variations.

The locations and types of mutations and structural variations in *CTNNB1* (**a**) and *APC* (**b**) are shown. Numbers depicted in circles representing missense mutations indicate the number of cases harboring mutations in these positions. aa, amino acid.

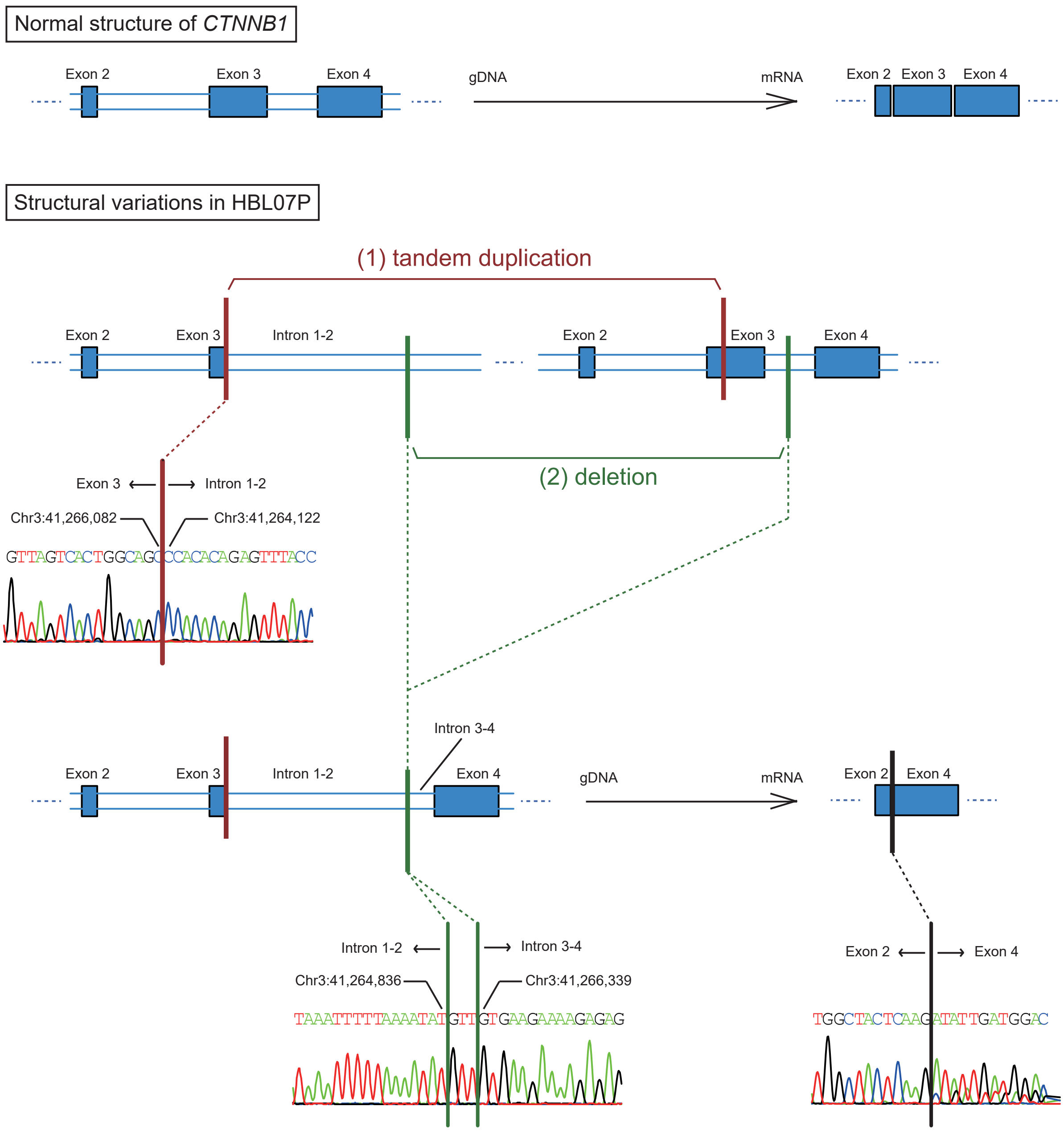

Supplementary Figure 2.

Unique combination of tandem duplication and long deletion in *CTNNB1* in case HBL07P.

Case HBL07P harbors tandem duplication from chr3:41,264,122 (within intron 1-2 of CTNNB1) to chr3:41,266,082 (within exon 3) followed by deletion from chr3:41,264,837 (within intron 1-2) to chr3:41,266,339 (within intron 3-4), which results in skipping of exon 3 in the mRNA. Sanger sequencing histograms of the regions surrounding the breakpoints are also shown.

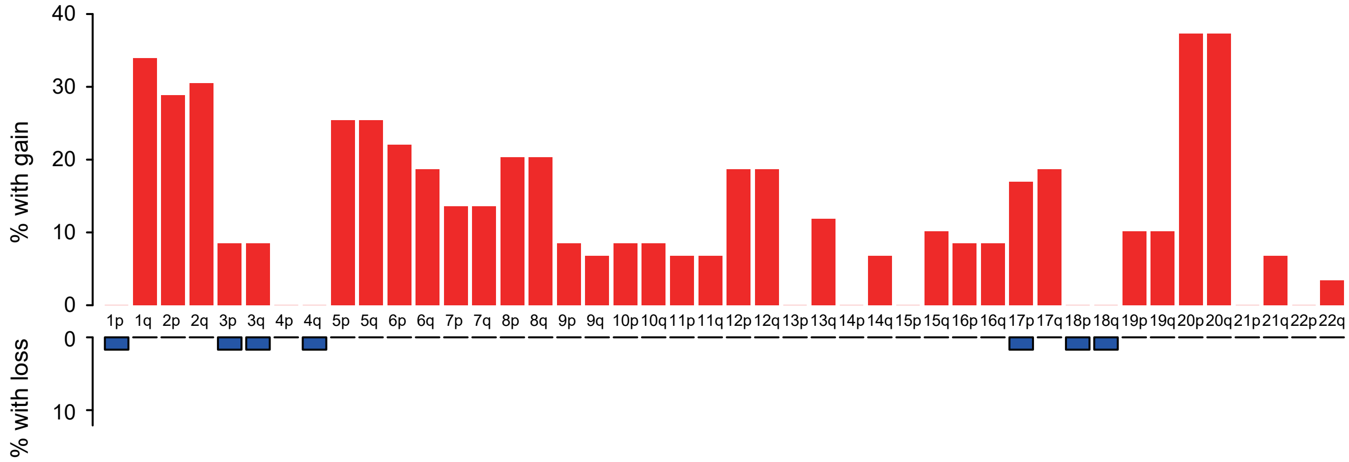

Supplementary Figure 3.

Spectrum of whole-arm copy number changes across the genome in hepatoblastoma.

Frequencies of whole-arm gains and losses in each chromosomal arm among 59 hepatoblastoma samples are displayed. Whole-arm gain/loss is defined as a copy number gain/loss of >95% of the bases of each chromosomal arm.

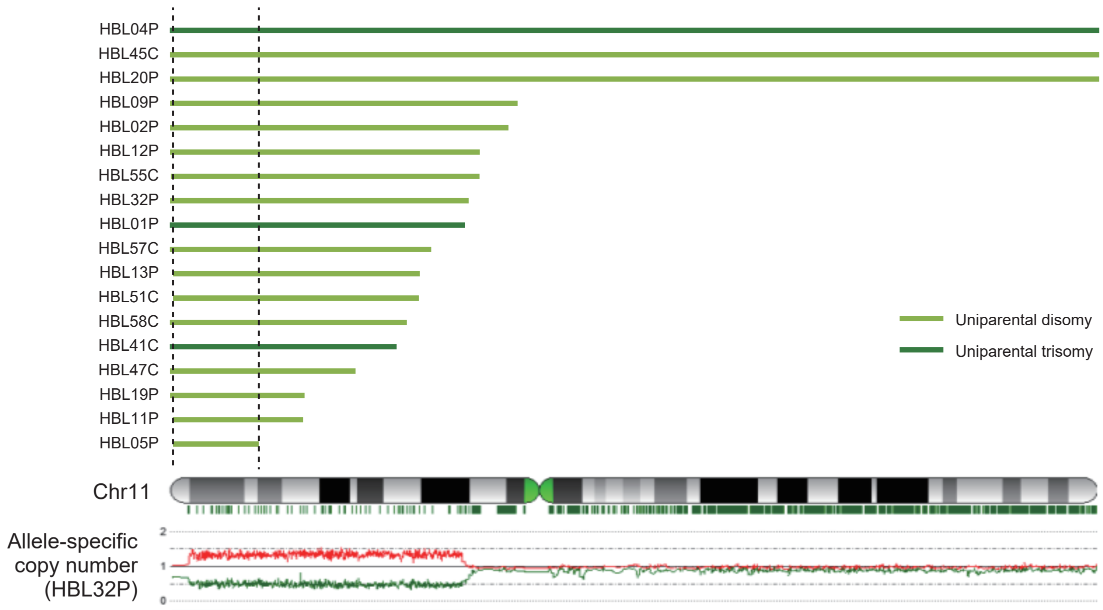

Supplementary Figure 4.

Hepatoblastoma samples harboring uniparental disomy/trisomy in chromosome 11.

Each green bar represents the affected region in each case and the region enclosed by dotted lines indicates the common region (chr11:672,443-13,122,171). Allele-specific red/green signals in case HBL32P are shown at the bottom as an example.

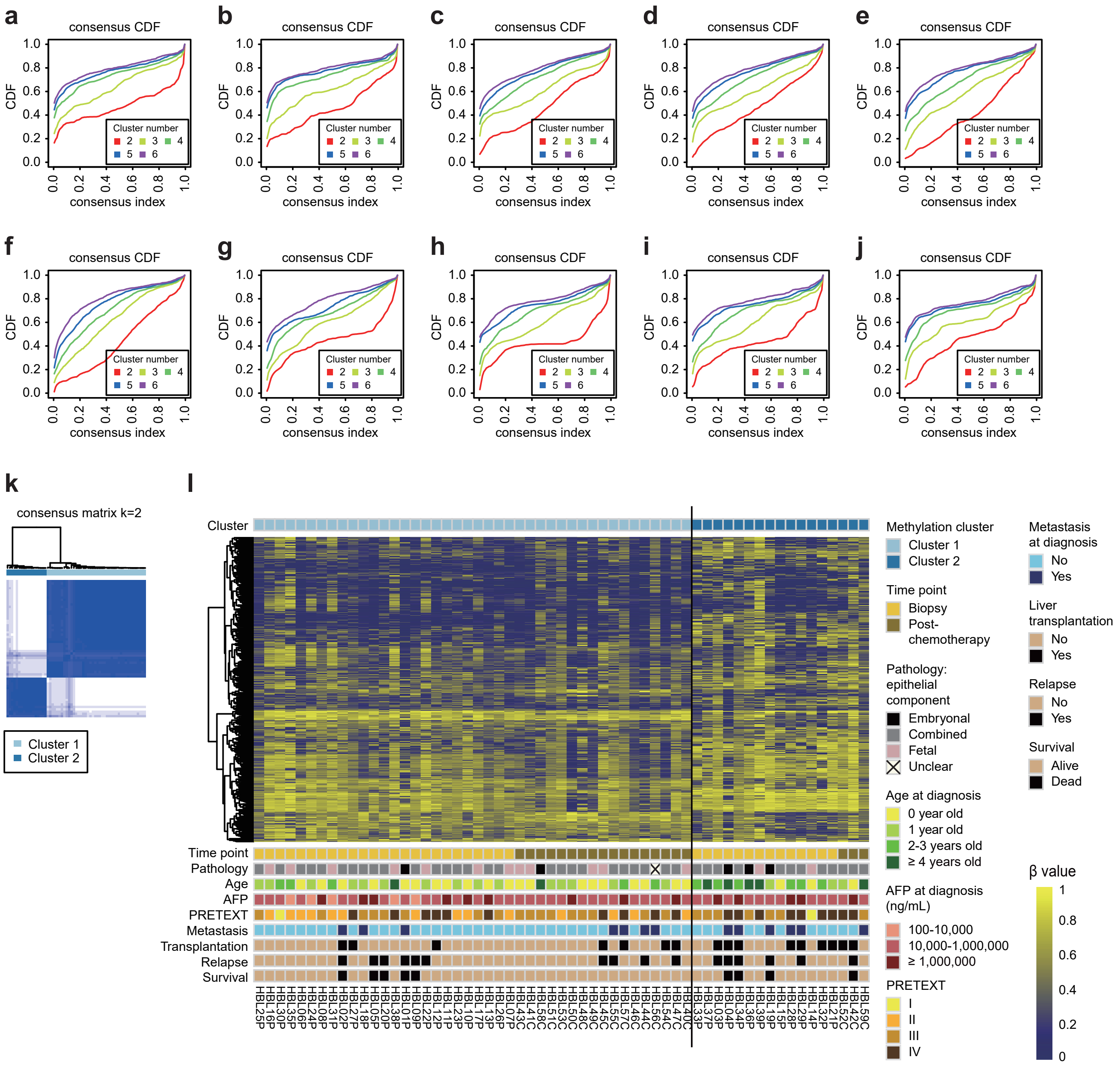

Supplementary Figure 5.

Consensus clustering of the DNA methylation data of 59 hepatoblastoma samples.

a–**j,** cumulative distribution function (CDF) plots corresponding to consensus clustering of top 100 (**a**,**f**), 1,000 (**b**,**g**), 2,000 (**c**,**h**), 3,000 (**d**,**i**) and 10,000 (**e**,**j**) CpG probes ranked by the SD of methylation beta values within Euclidean (**a**–**e**) and Pearson correlation metrics (**f**–**j**). The plots show that the most robust clustering is obtained with the top 2,000 probes and Pearson correlation metrics (**h**), which generates two stable clusters. **k**, consensus matrix corresponding to the consensus clustering using the top 2,000 probes and Pearson correlation metrics to generate two clusters. **l**, DNA methylation heatmap of the top 2,000 probes ranked by the SD across 59 hepatoblastoma samples and clinical information of each case. The most stable two clusters determined by the consensus clustering do not appear meaningful based on the lack of a clear difference in the clinical features between the clusters. AFP, alpha-fetoprotein; PRETEXT, pretreatment extent of disease.

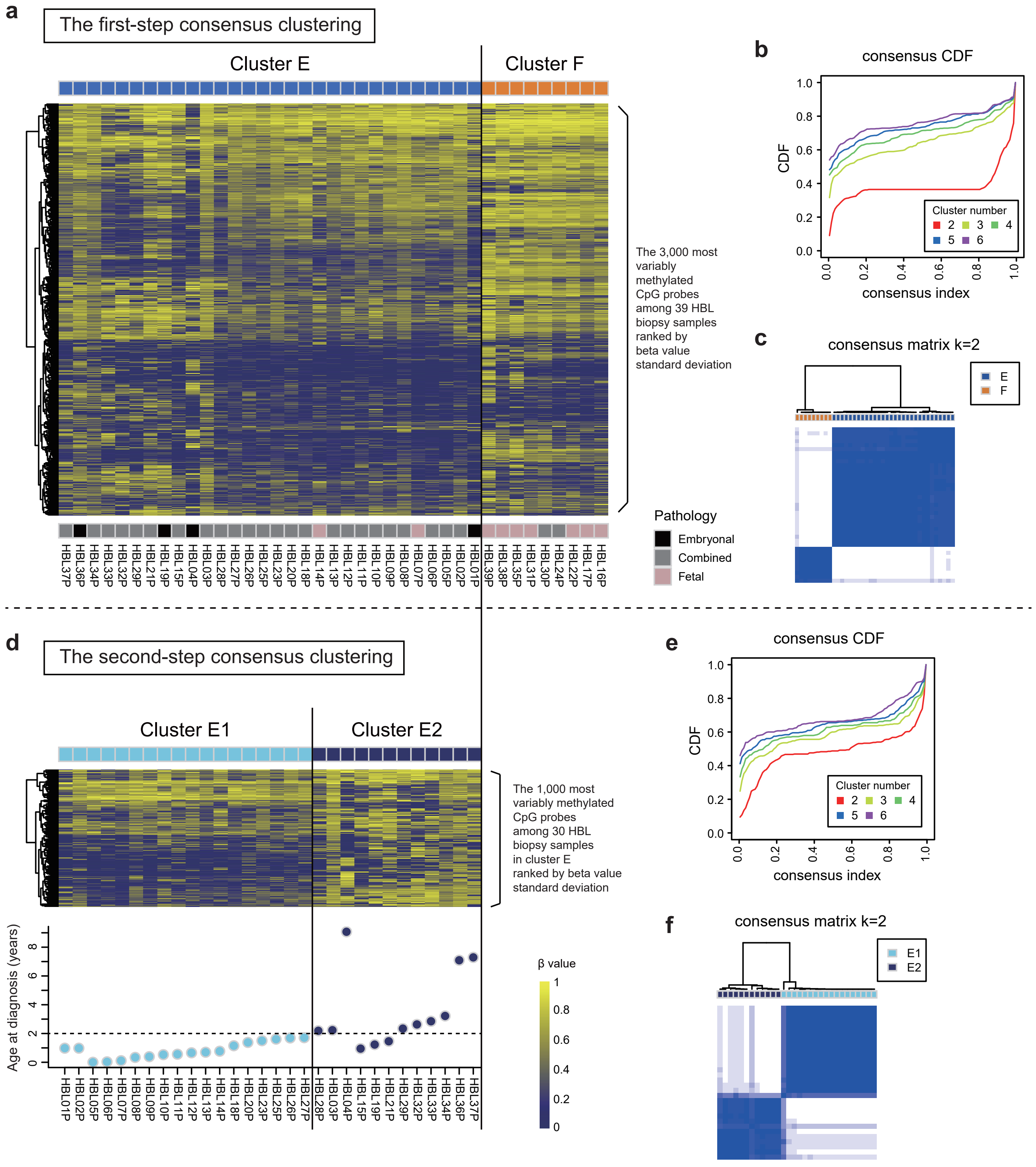

Supplementary Figure 6.

Consensus clustering of the DNA methylation data of hepatoblastoma biopsy samples.

Methylation heatmaps (**a**,**d**), cumulative distribution function (CDF) plots (**b**,**e**), and consensus matrices (**c**,**f**) are shown for the first-step consensus clustering of the 39 biopsy samples into two stable clusters, F and E (**a**–**c**), and the second-step consensus clustering of the 30 cluster E samples into two subgroups, E1 and E2 (**d**–**f**).

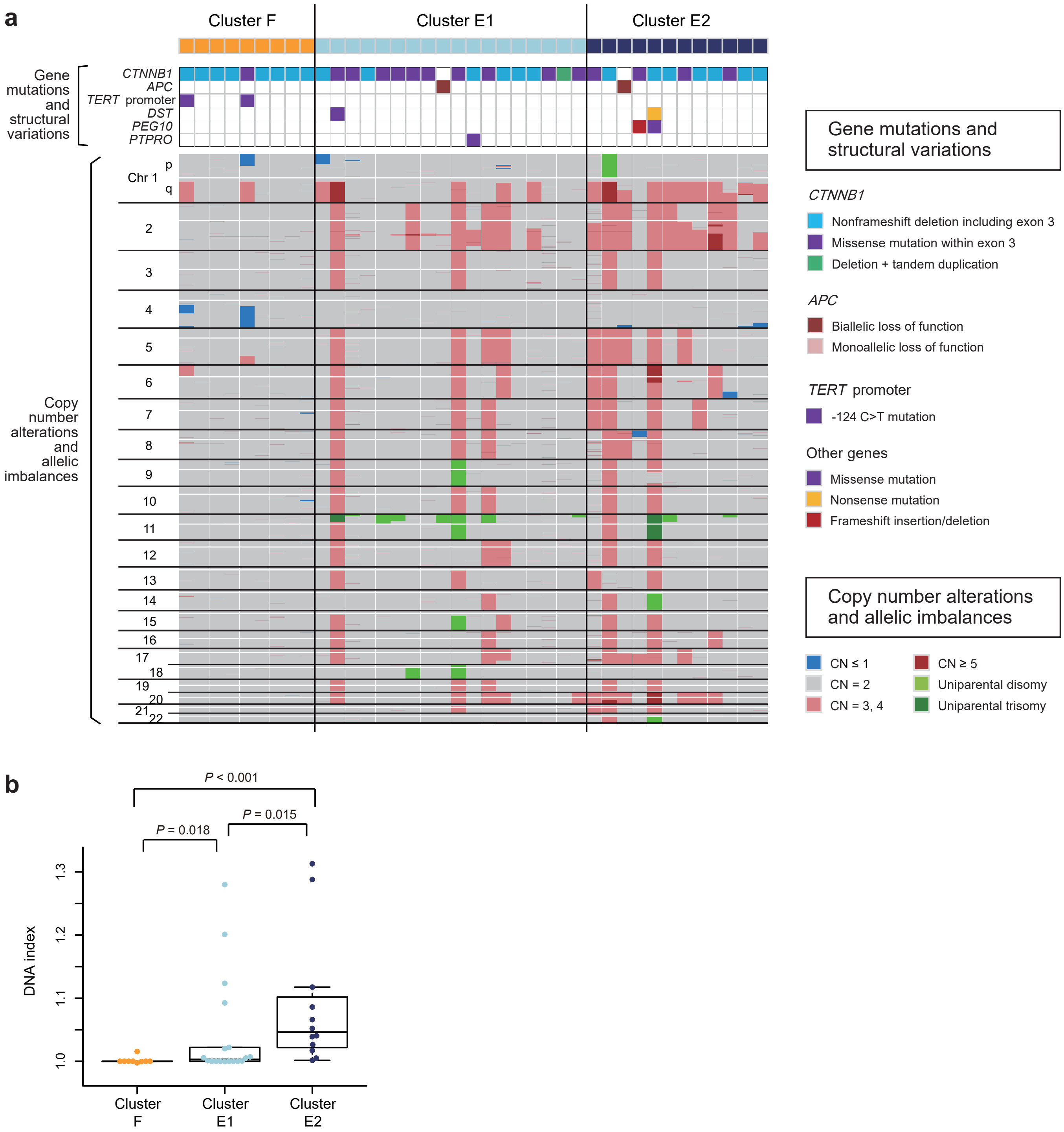

Supplementary Figure 7.

Profiles of mutations, copy number (CN) alterations, and allelic imbalances according to the methylation clusters.

a, landscape of mutations, CN alterations, and allelic imbalances according to the methylation clusters. **b**, comparison of DNA indices among the hepatoblastoma clusters using the Wilcoxon rank-sum test. DNA index was calculated as the average copy number across all chromosomes except X and Y divided by two. For the box plots, the middle line is the median, the top and bottom of the box indicate the interquartile range, and the error bars are minimum and maximum values excluding outliers.

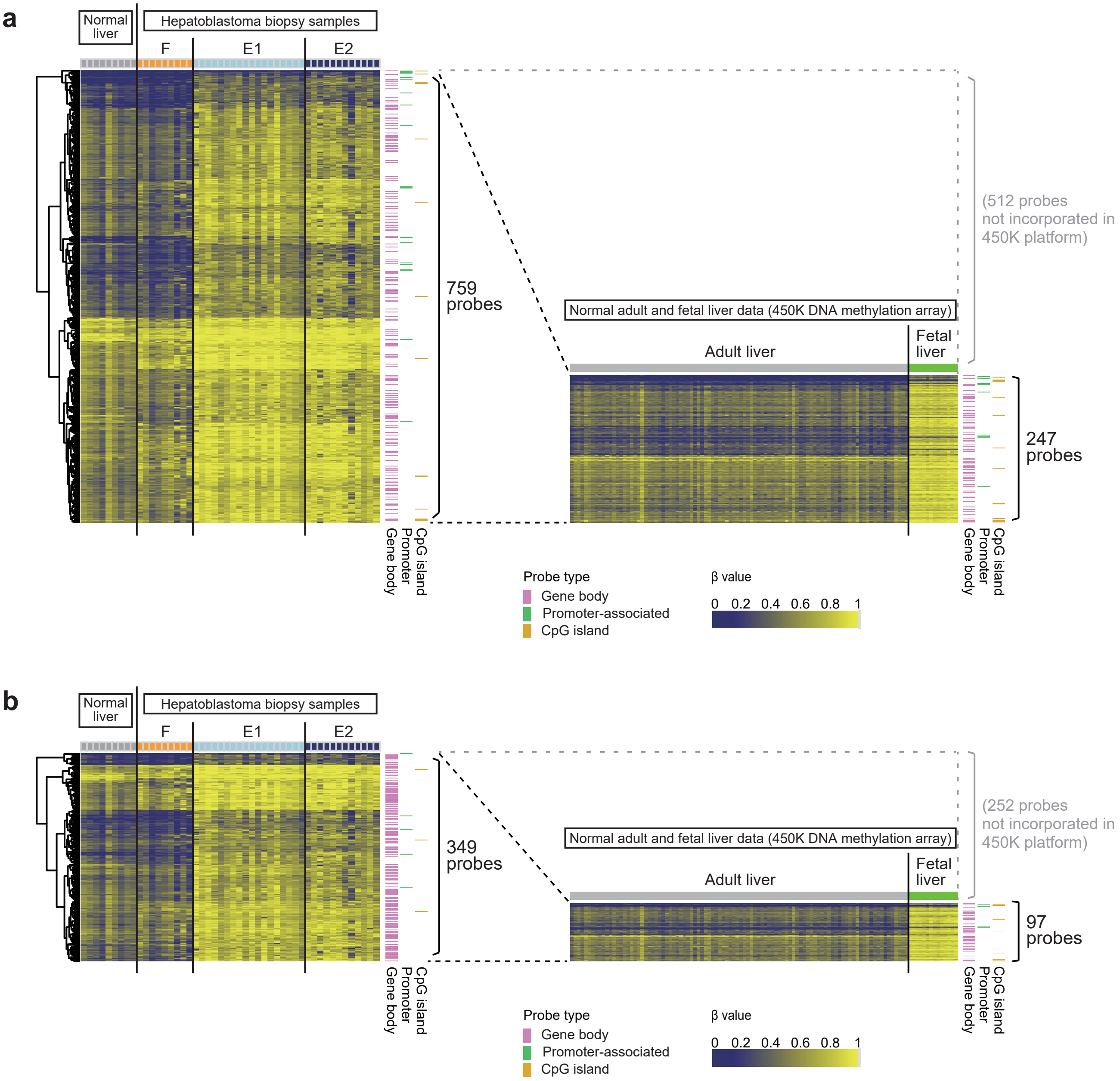

Supplementary Figure 8.

Differential methylation patterns of the HNF4A/CEBPA-binding regions between the hepatoblastoma clusters F versus E1/E2 are similar to those between the normal adult and fetal livers.

a, methylation heatmap on the left is constructed across 39 hepatoblastoma and nine NL samples, representing 759 differentially hypermethylated probes in clusters E1/E2 relative to cluster F that are overlapping with the HNF4A-binding regions (described as “cistrome_cistrome normal liver cells HNF4A” in the LOLA core database). Heatmap on the right is constructed across normal adult and fetal liver samples, representing 247 probes among the 759 probes incorporated in the 450K methylation array.

b, methylation heatmap on the left is constructed across 39 hepatoblastoma and nine NL samples, representing 349 differentially hypermethylated probes in clusters E1/E2 relative to cluster F and overlapping with the CEBPA-binding regions (described as “cistrome_cistrome normal liver cells CEBPA” in the LOLA core database). Heatmap on the right is constructed across normal adult and fetal liver samples, representing 97 probes among the 349 probes incorporated in the 450K methylation array.

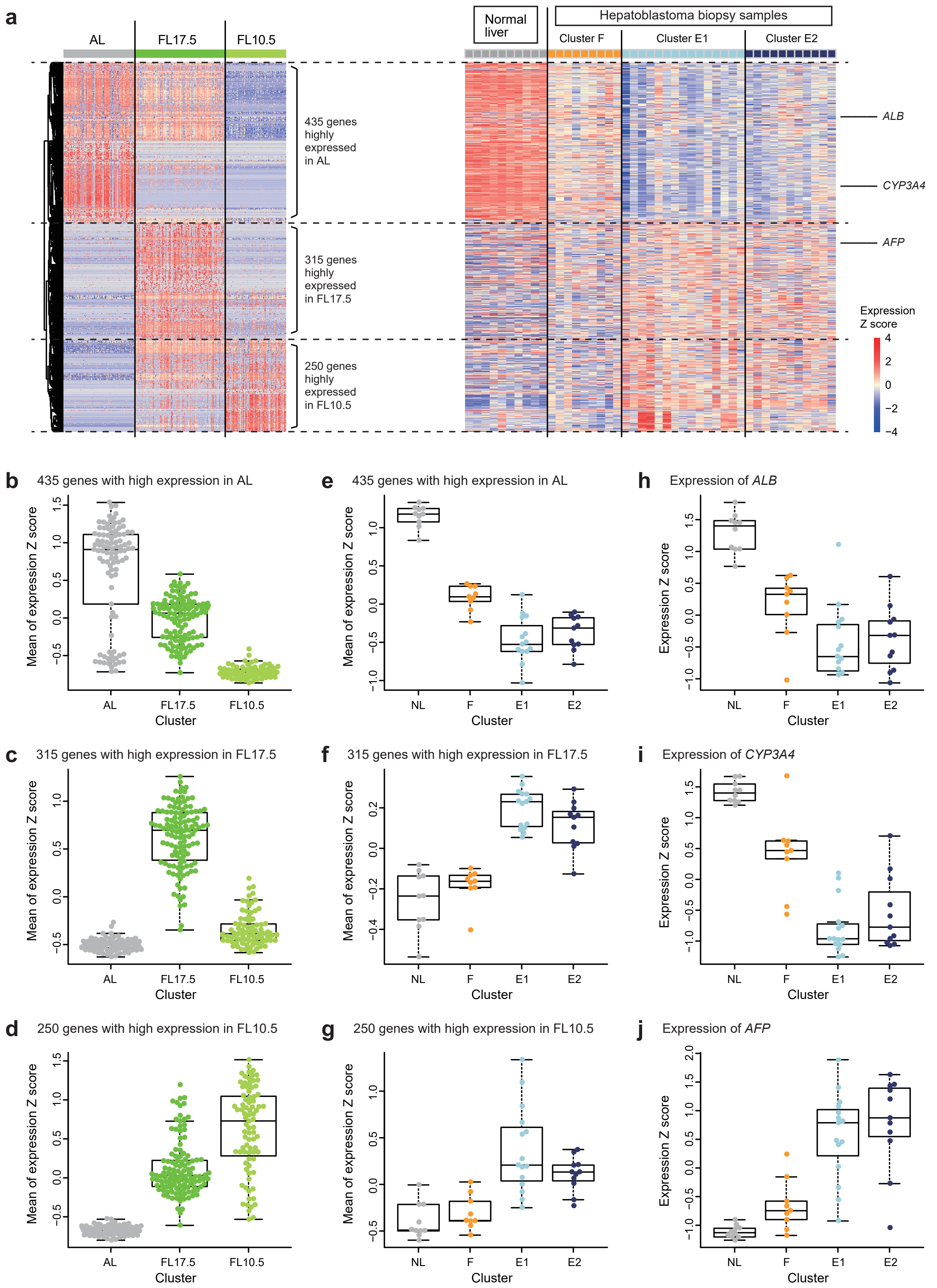
a
AL
FL17.5
FL10.5
435 genes highly expressed in AL
315 genes highly expressed in FL17.5
250 genes highly expressed in FL10.5
Normal liver
Hepatoblastoma biopsy samples
Cluster F
Cluster E1
Cluster E2
ALB
CYP3A4
AFP
Expression Z score
b
435 genes with high expression in AL
Mean of expression Z score
Cluster
c
315 genes with high expression in FL17.5
d
250 genes with high expression in FL10.5
e
f
g
NL
F
E1
E2
h
Expression of ALB
Expression Z score
i
Expression of CYP3A4
j
Expression of AFP

Supplementary Figure 9.

Differentiation diversity between the hepatoblastoma methylation clusters confirmed by gene expression profiles.

a, expression heatmap on the left represents top 1,000 differentially expressed genes among adult liver (AL), fetal liver at 17.5 weeks of gestation (FL17.5), and fetal liver at 10.5 weeks of gestation (FL10.5), whereas heatmap on the right is constructed for the same 1,000 genes across 35 hepatoblastoma and 10 NL samples. **b**–**g**, mean expression Z scores of 435 genes highly expressed in AL (**b**,**e**), 315 genes highly expressed in FL17.5 (**c**,**f**), and 250 genes highly expressed in FL10.5 (**d**,**g**) are plotted with comparison to normal adult and fetal livers (**b**–**d**), and the study cohort samples (**e**–**g**) according to the clusters. **h**–**j**, expression Z scores of three genes associated with liver development, ALB (**h**), CYP3A4 (**i**), and AFP (**j**), are plotted for the study cohort samples according to the clusters. For the box plots, the middle line is the median, the top and bottom of the box indicate the interquartile range, and the error bars are minimum and maximum values excluding outliers.

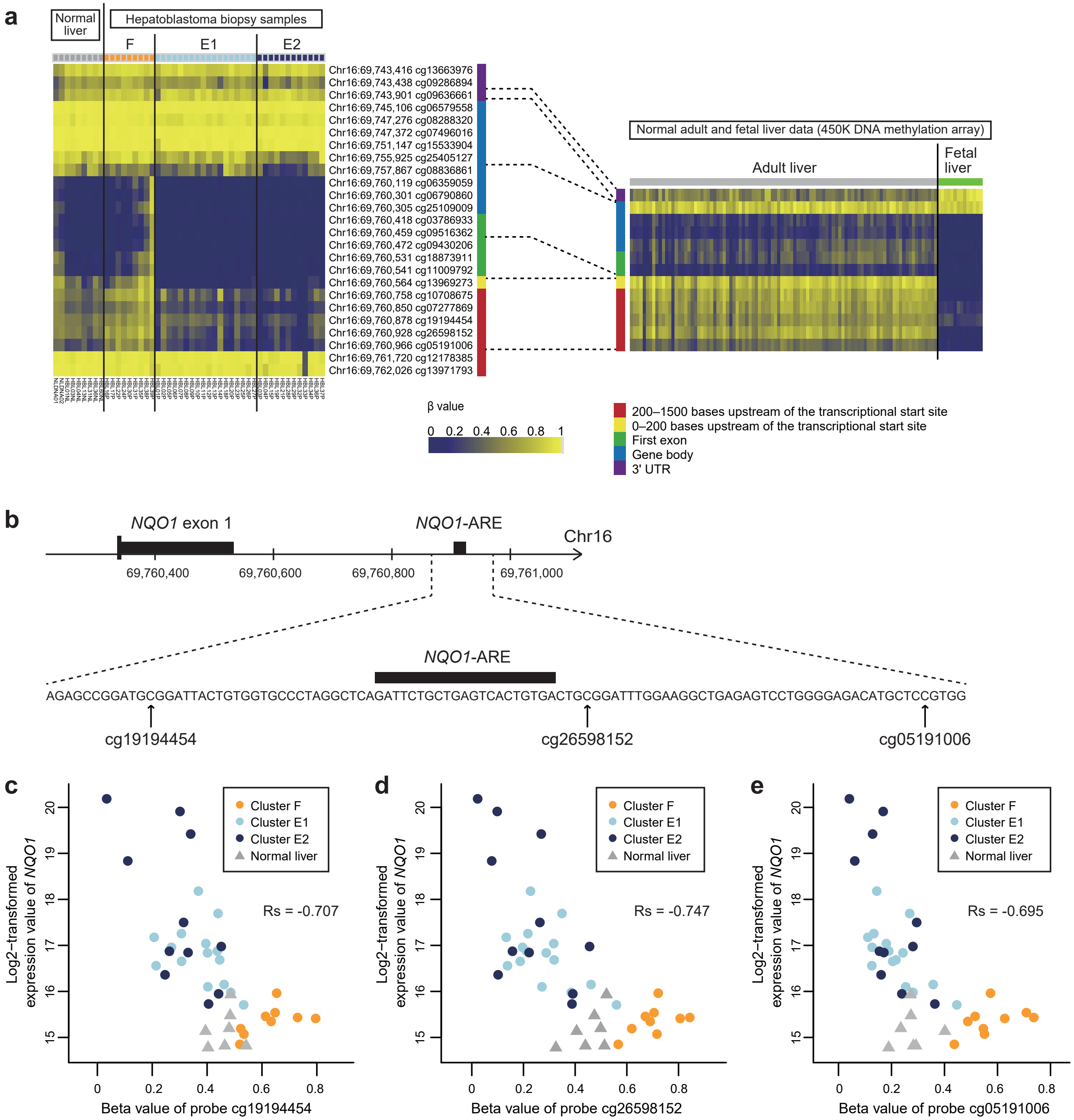

Supplementary Figure 10.

Differential methylation of the *NQO1* promoter region between hepatoblastoma clusters F and E1/E2.

a, methylation status of the CpG probes associated with *NQO1*. Heatmap on the left is constructed for the hepatoblastoma and normal liver samples in the study cohort, whereas heatmap on the right is constructed for the 450K methylation array data of normal adult and fetal livers. **b**, genomic positions of exon 1 and antioxidant response element (ARE) of *NQO1* and neighboring CpG probes (hg19). **c**–**e**, correlation between *NQO1* expression and methylation of probes cg19194454 (**c**), cg26598152 (**d**), and cg05191006 (**e**). Rs represents Spearman' s correlation coefficient. Methylation of cg26598152, the CpG probe nearest to the *NQO1*-ARE, exhibits the strongest correlation with *NQO1* expression.

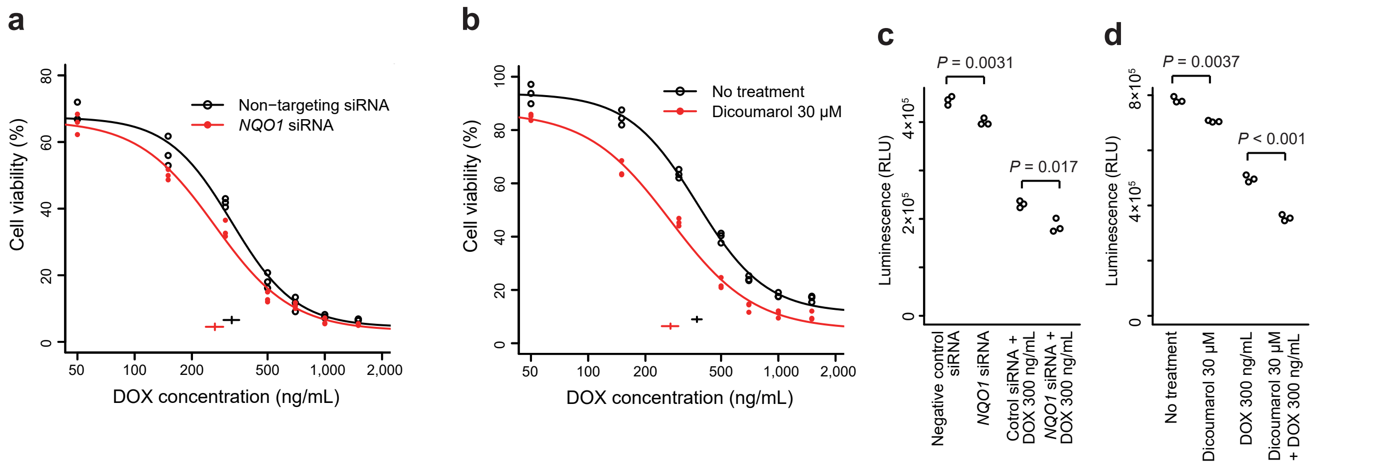

Supplementary Figure 11.

Chemosensitivity assay using the hepatoblastoma cell line HuH6.

a,**b**, dose-response curves of HuH6 cells treated with various concentrations of doxorubicin (DOX) after *NQO1* inhibition (red) or negative control treatment (black). *NQO1* was inhibited using siRNA (**a**) or dicoumarol (**b**). Horizontal bars and whiskers in the bottom indicate EC_{50} values with 95% confidence intervals. **c**,**d**, enhancement of DOX cytotoxicity by *NQO1* inhibition in HuH6 cells. *NQO1* was inhibited using siRNA (**c**) or dicoumarol (**d**). The luminescence intensities representing the cell viability are compared between the conditions with and without *NQO1* inhibition using the unpaired Student's t-test.

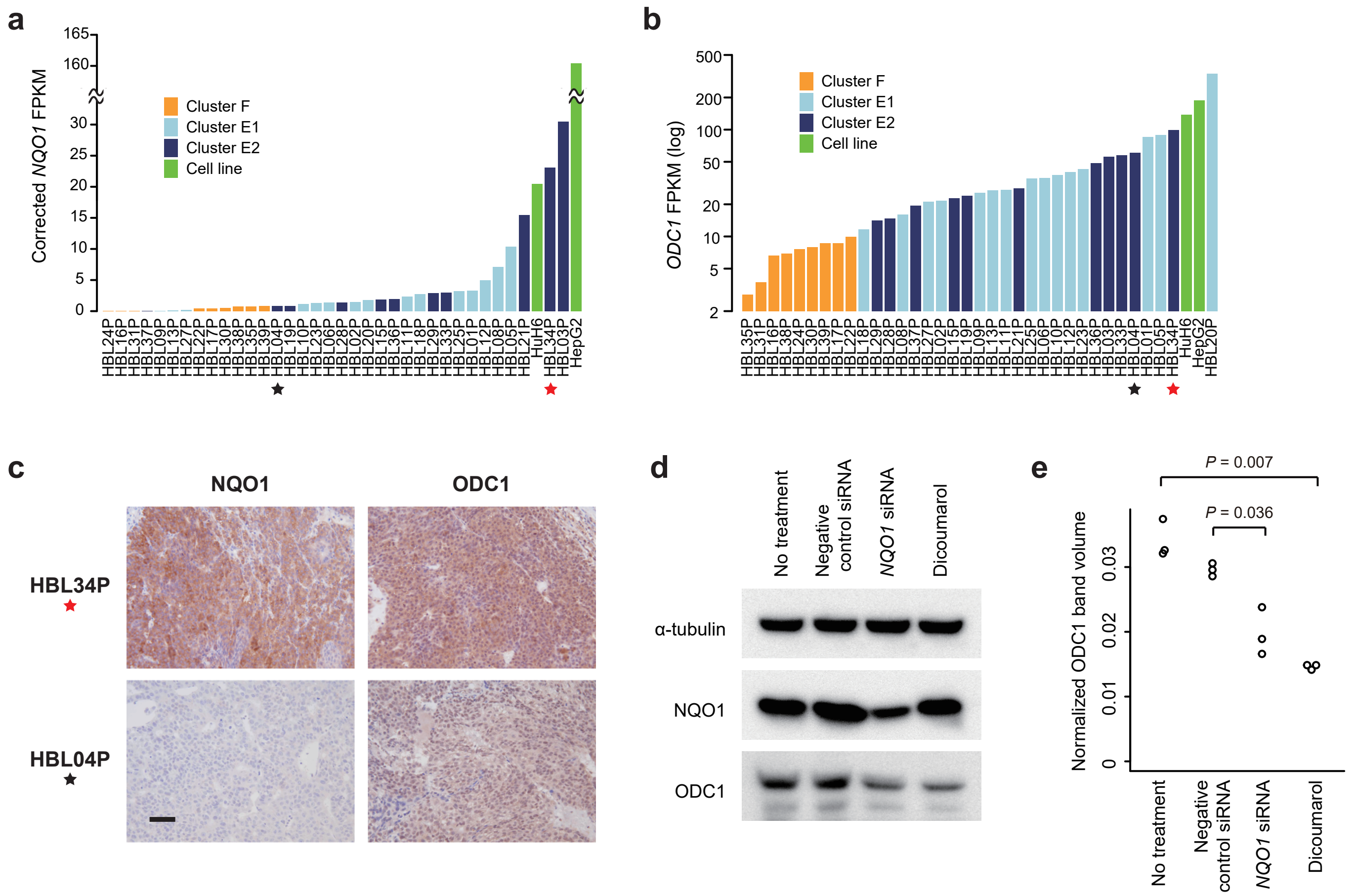

Supplementary Figure 12.

NQO1 stabilizes ODC1 in hepatoblastoma cells.

a,**b**, waterfall plots showing *NQO1* expression values (FPKM, fragments per kilobase of transcript per million mapped reads) corrected for the enzymatic activity-determining SNP (C609T; **a**) and *ODC1* FPKM (**b**) in hepatoblastoma samples and cell lines. HBL34P, which exhibits high expression of both *NQO1* and *ODC1* in mRNA levels, is marked with red stars, whereas HBL04P, which exhibits low mRNA level of *NQO1* and high mRNA level of *ODC1*, is marked with black stars. **c**, NQO1 and ODC1 immunostaining of the samples HBL34P and HBL04P. Scale bar represents 100 μm. ODC1 is strongly positive in HBL34P and slightly positive in HBL04P although they harbor comparably high mRNA levels of *ODC1*, which indicates that the difference in *NQO1* expression makes a difference in the stability of ODC1 in the protein level. **d**, Western blot analysis of alpha-tubulin, NQO1, and ODC1 in HepG2 cells with and without *NQO1* inhibition. **e**, ODC1 band intensity normalized by that of alpha-tubulin in HepG2 cells. The intensities are compared between the conditions with and without *NQO1* inhibition using the unpaired Student's t-test.

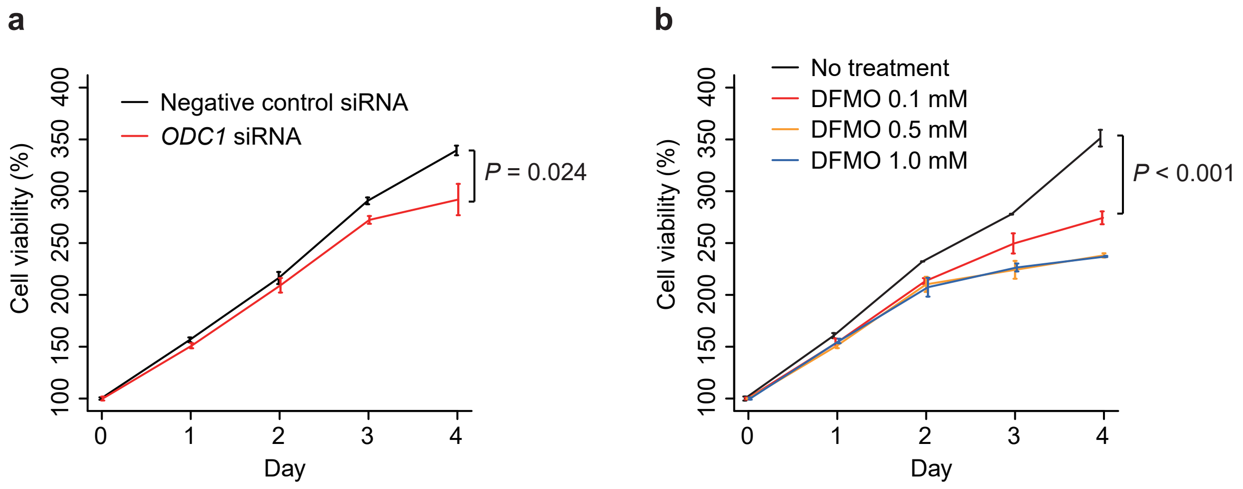

Supplementary Figure 13.

Cell proliferation assay to assess the effect of *ODC1* inhibition in HuH6.

ODC1 was inhibited using siRNA (**a**) or difluoromethylornithine (DFMO, **b**). Error bars indicate standard deviations of triplicate experiments. Cell viabilities on day 4 are compared between the conditions using the unpaired Student's t-test.

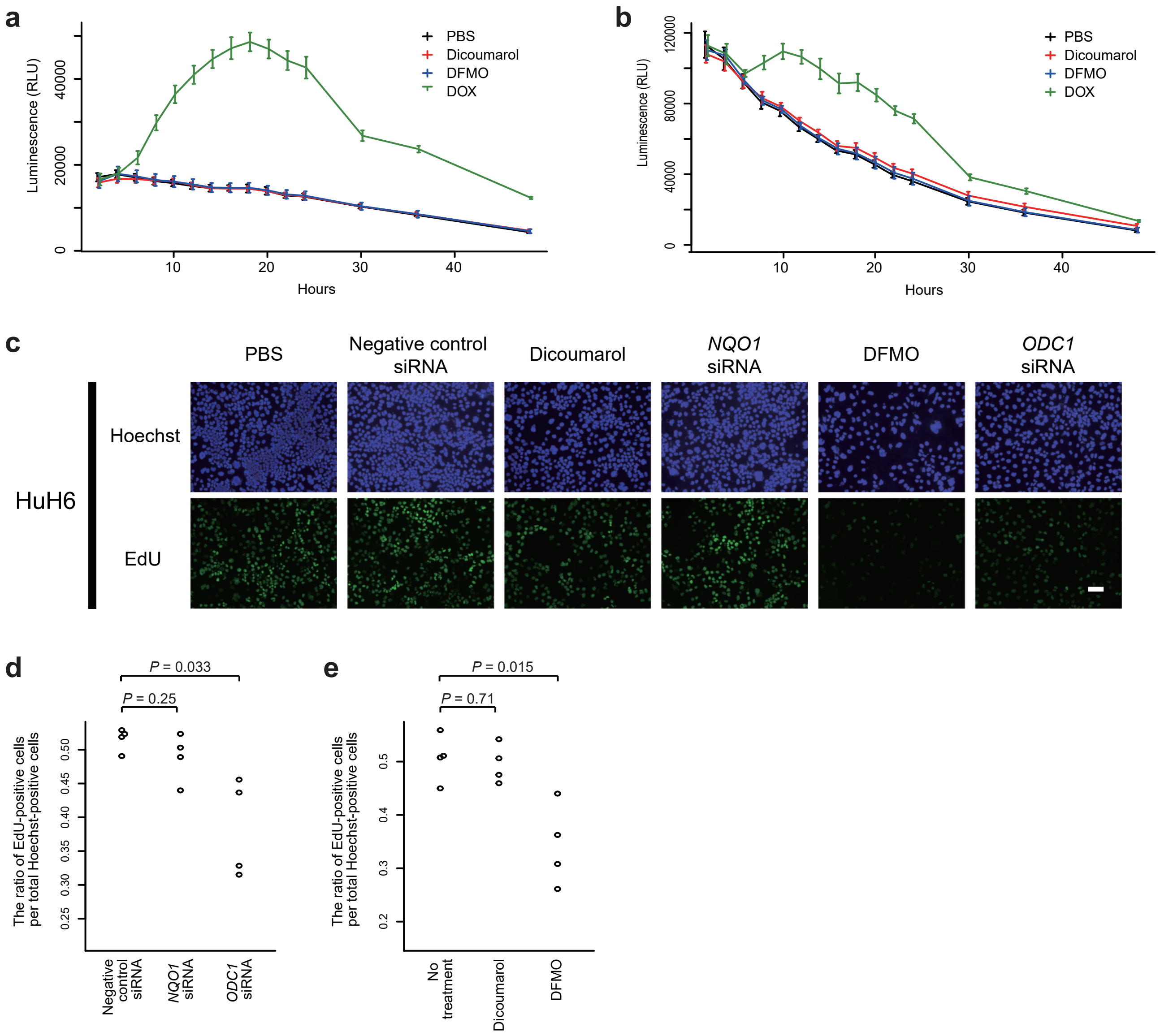

Supplementary Figure 14.

Apoptosis and cell cycle assays using hepatoblastoma cell lines.

a,**b**, apoptosis assay using hepatoblastoma cell lines HepG2 (**a**) and HuH6 (**b**). Apoptosis signals are compared among the hepatoblastoma cells treated with PBS (negative control), dicoumarol (*NQO1* inhibitor), difluoromethylornithine (DFMO; *ODC1* inhibitor), and a high concentration of doxorubicin (DOX; positive control). Error bars indicate standard deviations of hexaplicate experiments. **c**–**e**, ethynyl deoxyuridine (EdU) assay using HuH6 cells treated with PBS, dicoumarol, DFMO, and negative control/*NQO1*/*ODC1* siRNA. The ratio of EdU-positive cells per total Hoechst-positive cells are compared among the conditions using the unpaired Student' s t-test. Scale bar represents 100 μm.

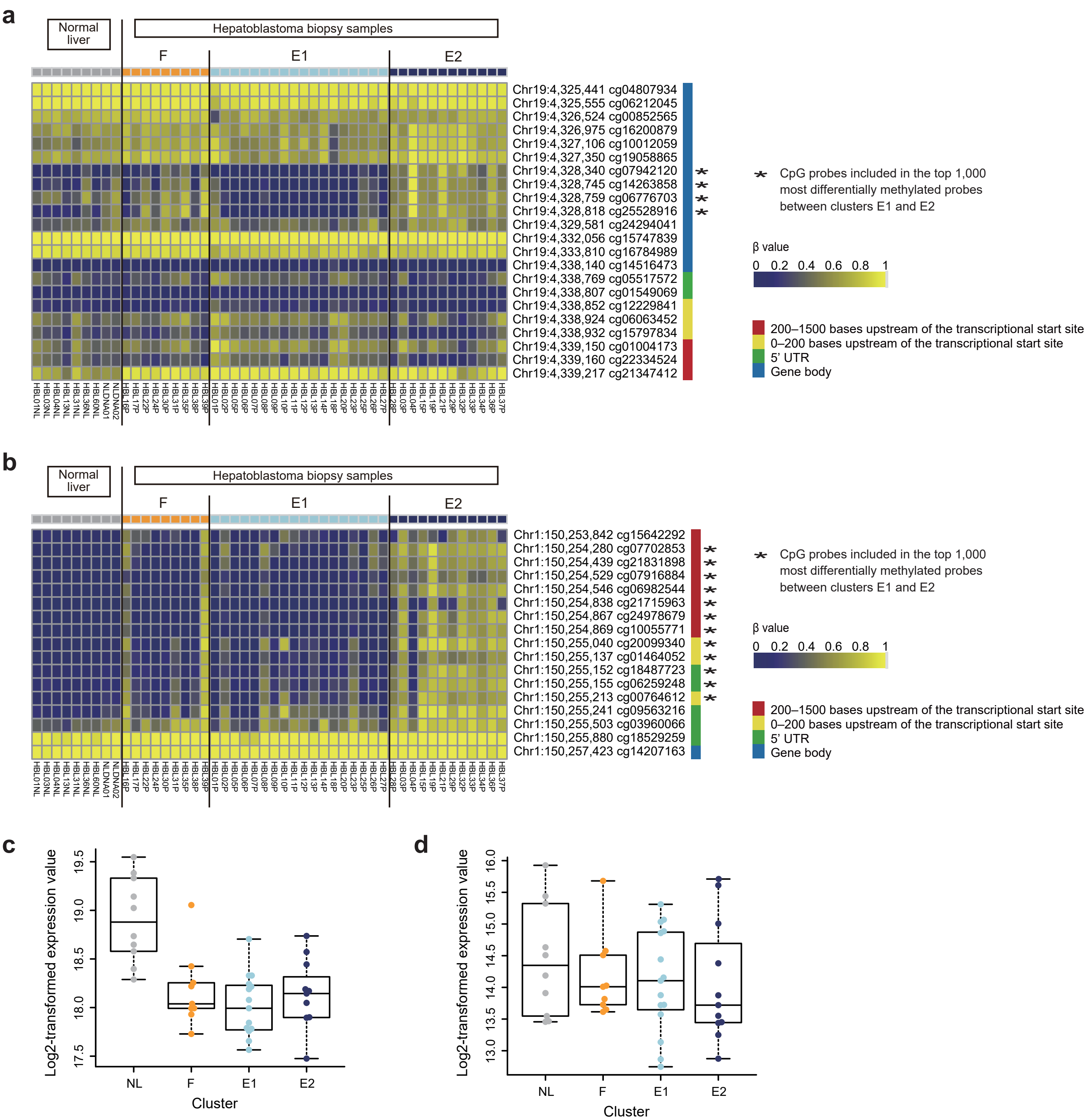

Supplementary Figure 15.

Differential methylation of *STAP2* and *C1orf51/CIART* between clusters E1 and E2.

a,**b**, Methylation status of the CpG probes associated with *STAP2* (**a**) and *C1orf51/CIART* (**b**), the most significantly differentially methylated genes between hepatoblastoma clusters E1 and E2. Heatmaps are constructed for the 39 hepatoblastoma biopsy samples and nine normal liver samples in the study cohort. CpGs included in the 1,000 most differentially methylated probes between clusters E1 and E2 (Supplementary Data S3) are marked with asterisks (*). **c**,**d** Expression of *STAP2* (**c**) and *C1orf51/CIART* (**d**) according to the clusters. For the box plots, the middle line is the median, the top and bottom of the box indicate the interquartile range, and the error bars are minimum and maximum values excluding outliers. NL, normal liver.

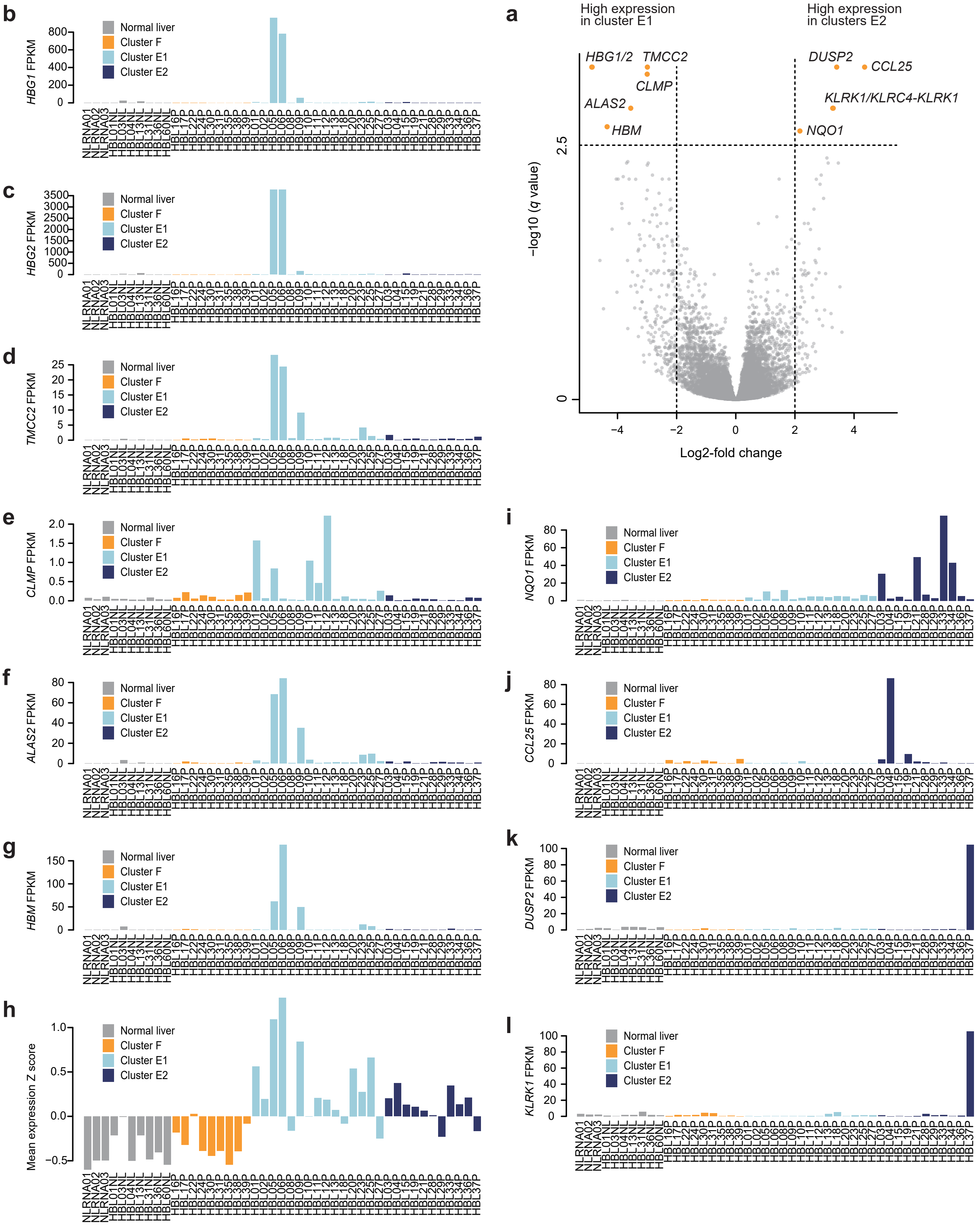

a
High expression in cluster E1
High expression in clusters E2
HBG1/2
TMCC2
CLMP
ALAS2
HBM
DUSP2
CCL25
KLRK1/KLRC4-KLRK1
NQO1
−log10 (q value)
Log2-fold change
b
HBG1 FPKM
c
HBG2 FPKM
d
TMCC2 FPKM
e
CLMP FPKM
f
ALAS2 FPKM
g
HBM FPKM
h
Mean expression Z score
i
NQO1 FPKM
j
CCL25 FPKM
k
DUSP2 FPKM
l
KLRK1 FPKM
Normal liver
Cluster F
Cluster E1
Cluster E2

Supplementary Figure 16.

Differential expression between clusters E1 and E2.

a, Volcano plot displaying genes that are differentially expressed between the hepatoblastoma clusters E1 and E2. Each gene is plotted with log2-fold expression change on x-axis and negative log10 false discovery rate (FDR) q value on y-axis. Genes with absolute log2-fold change >2 and negative log10 FDR q value >2.5 are shown in orange. **b**–**g**,**i**–**l**, expression values (FPKM, fragments per kilobase of transcript per million mapped reads) of the differentially expressed genes between clusters E1 and E2: *HBG1* (**b**); *HBG2* (**c**); *TMCC2* (**d**); *CLMP* (**e**); *ALAS2* (**f**); *HBM* (**g**); *NQO1* (**i**); *CCL25* (**j**); *DUSP2* (**k**); and *KLRK1* (**l**).
h, Mean expression Z scores of the 250 genes differentially highly expressed in normal fetal liver at 10.5 weeks of gestation compared with normal adult liver and fetal liver at 17.5 weeks of gestation (shown in Supplementary Data S9).

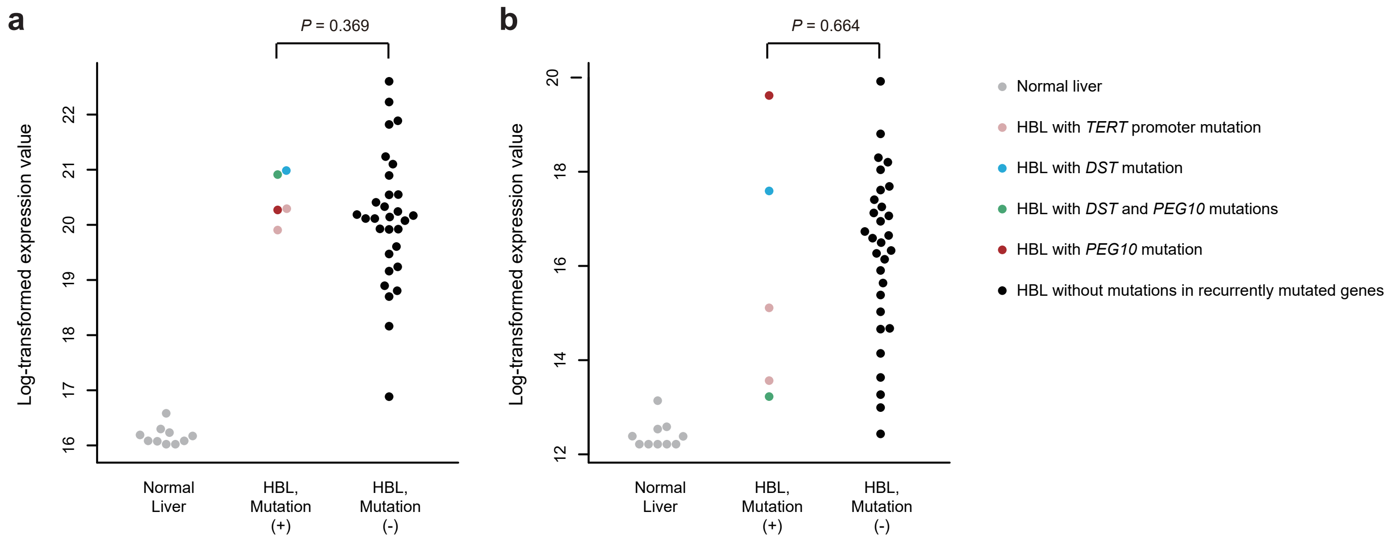

Supplementary Figure 17.

Comparison of the expression levels of the Wnt target genes.

Log-transformed expression values of the Wnt target genes *DKK1* (**a**) and *WIF1* (**b**) are compared among normal liver, hepatoblastoma (HBL) with mutations in the recurrently mutated genes (*DST*, *PEG10*, and the *TERT* promoter), and the other HBL samples using the Wilcoxon rank-sum test.

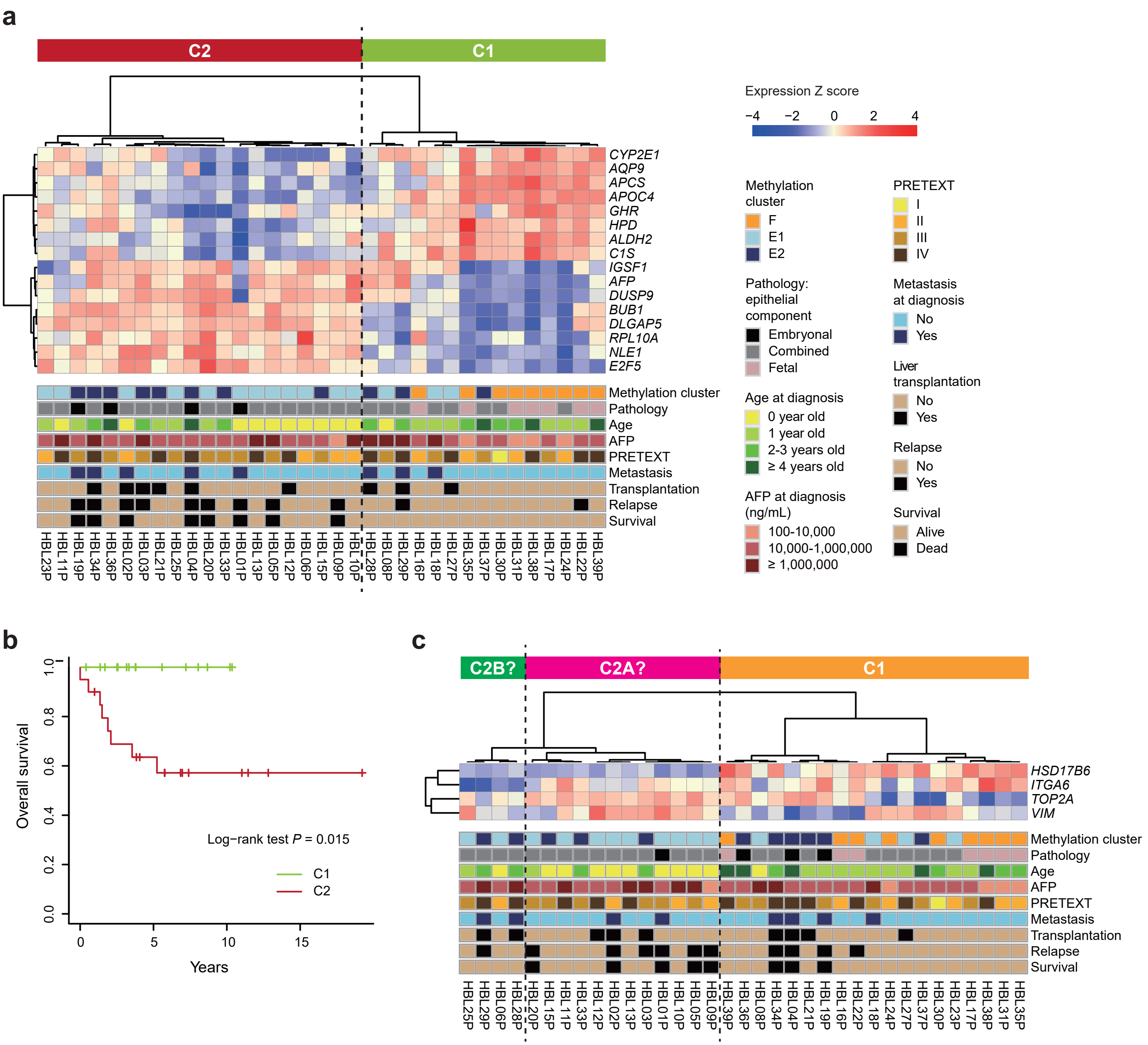

Supplementary Figure 18.

Comparison of the genetic classifications of hepatoblastoma in the present and previous studies.

a,**b**, the C1/C2 classification described by Cairo et al. is applied to our cohort and compared to the methylation clusters proposed in the present study. **a**, expression heatmap of 16 genes used in the C1/C2 classification across 35 hepatoblastoma biopsy samples in our cohort. The methylation clusters F and E1/E2 fairly overlap with the C1 and C2, respectively. **b**, Kaplan-Meier survival curves of the C1 and C2 subclasses. **c**, expression heatmap of four genes used in C1/C2A/C2B classification proposed by Hooks et al. across 35 biopsy samples in our cohort. According to the original study, the C1 subclass is characterized by high expression of *HSD17B6*, whereas the C2A and C2B are defined by coexpression of *ITGA6*/*TOP2A* and high expression of *VIM*, respectively. In the heatmap shown here, however, the C2A and C2B are not well specified. AFP, alpha-fetoprotein; PRETEXT, pretreatment extent of disease.

Supplementary Table 1. Samples subjected to massive parallel sequencing and microarray analysis.

Patient ID	Sample ID	Sample type	Targeted capture sequencing	SNP array	DNA methylation array	RNA sequencing
HBL01	HBL01P	Primary diagnostic biopsy	Yes	Yes	Yes	Yes
HBL02	HBL02P	Primary diagnostic biopsy	Yes	Yes	Yes	Yes
HBL03	HBL03P	Primary diagnostic biopsy	Yes	Yes	Yes	Yes
HBL04	HBL04P	Primary diagnostic biopsy	Yes	Yes	Yes	Yes
HBL05	HBL05P	Primary diagnostic biopsy	Yes	Yes	Yes	Yes
HBL06	HBL06P	Primary diagnostic biopsy	Yes	Yes	Yes	Yes
HBL07	HBL07P	Primary diagnostic biopsy	Yes	Yes	Yes	No
HBL08	HBL08P	Primary diagnostic biopsy	Yes	Yes	Yes	Yes
HBL09	HBL09P	Primary diagnostic biopsy	Yes	Yes	Yes	Yes
HBL10	HBL10P	Primary diagnostic biopsy	Yes	Yes	Yes	Yes
HBL11	HBL11P	Primary diagnostic biopsy	Yes	Yes	Yes	Yes
HBL12	HBL12P	Primary diagnostic biopsy	Yes	Yes	Yes	Yes
HBL13	HBL13P	Primary diagnostic biopsy	Yes	Yes	Yes	Yes
HBL14	HBL14P	Primary diagnostic biopsy	Yes	Yes	Yes	No
HBL15	HBL15P	Primary diagnostic biopsy	Yes	Yes	Yes	Yes
HBL16	HBL16P	Primary diagnostic biopsy	Yes	Yes	Yes	Yes
HBL17	HBL17P	Primary diagnostic biopsy	Yes	Yes	Yes	Yes
HBL18	HBL18P	Primary diagnostic biopsy	Yes	Yes	Yes	Yes
HBL19	HBL19P	Primary diagnostic biopsy	Yes	Yes	Yes	Yes
HBL20	HBL20P	Primary diagnostic biopsy	Yes	Yes	Yes	Yes
HBL21	HBL21P	Primary diagnostic biopsy	Yes	Yes	Yes	Yes
HBL22	HBL22P	Primary diagnostic biopsy	Yes	Yes	Yes	Yes
HBL23	HBL23P	Primary diagnostic biopsy	Yes	Yes	Yes	Yes
HBL24	HBL24P	Primary diagnostic biopsy	Yes	Yes	Yes	Yes
HBL25	HBL25P	Primary diagnostic biopsy	Yes	Yes	Yes	Yes
HBL26	HBL26P	Primary diagnostic biopsy	Yes	Yes	Yes	No
HBL27	HBL27P	Primary diagnostic biopsy	Yes	Yes	Yes	Yes
HBL28	HBL28P	Primary diagnostic biopsy	Yes	Yes	Yes	Yes
HBL29	HBL29P	Primary diagnostic biopsy	Yes	Yes	Yes	Yes
HBL30	HBL30P	Primary diagnostic biopsy	Yes	Yes	Yes	Yes
HBL31	HBL31P	Primary diagnostic biopsy	Yes	Yes	Yes	Yes
HBL32	HBL32P	Primary diagnostic biopsy	Yes	Yes	Yes	No
HBL33	HBL33P	Primary diagnostic biopsy	Yes	Yes	Yes	Yes
HBL34	HBL34P	Primary diagnostic biopsy	Yes	Yes	Yes	Yes
HBL35	HBL35P	Primary diagnostic biopsy	Yes	Yes	Yes	Yes
HBL36	HBL36P	Primary diagnostic biopsy	Yes	Yes	Yes	Yes
HBL37	HBL37P	Primary diagnostic biopsy	Yes	Yes	Yes	Yes
HBL38	HBL38P	Primary diagnostic biopsy	Yes	Yes	Yes	Yes
HBL39	HBL39P	Primary diagnostic biopsy	Yes	Yes	Yes	Yes
HBL40	HBL40C	Post-chemotherapy resection	Yes	Yes	Yes	No
HBL41	HBL41C	Post-chemotherapy resection	Yes	Yes	Yes	Yes

HBL42	HBL42C	Post-chemotherapy resection	Yes	Yes	Yes	Yes
HBL43	HBL43C	Post-chemotherapy resection	Yes	Yes	Yes	Yes
HBL44	HBL44C	Post-chemotherapy resection	Yes	Yes	Yes	No
HBL45	HBL45C	Post-chemotherapy resection	Yes	Yes	Yes	Yes
HBL46	HBL46C	Post-chemotherapy resection	Yes	Yes	Yes	Yes
HBL47	HBL47C	Post-chemotherapy resection	Yes	Yes	Yes	Yes
HBL48	HBL48C	Post-chemotherapy resection	Yes	Yes	Yes	Yes
HBL49	HBL49C	Post-chemotherapy resection	Yes	Yes	Yes	Yes
HBL50	HBL50C	Post-chemotherapy resection	Yes	Yes	Yes	Yes
HBL51	HBL51C	Post-chemotherapy resection	Yes	Yes	Yes	Yes
HBL52	HBL52C	Post-chemotherapy resection	Yes	Yes	Yes	Yes
HBL53	HBL53C	Post-chemotherapy resection	Yes	Yes	Yes	Yes
HBL54	HBL54C	Post-chemotherapy resection	Yes	Yes	Yes	No
HBL55	HBL55C	Post-chemotherapy resection	Yes	Yes	Yes	Yes
HBL56	HBL56C	Post-chemotherapy resection	Yes	Yes	Yes	Yes
HBL57	HBL57C	Post-chemotherapy resection	Yes	Yes	Yes	No
HBL58	HBL58C	Post-chemotherapy resection	Yes	Yes	Yes	Yes
HBL59	HBL59C	Post-chemotherapy resection	Yes	Yes	Yes	No
HBL01	HBL01NL	Normal liver	No	No	Yes	Yes
HBL03	HBL03NL	Normal liver	No	No	Yes	Yes
HBL04	HBL04NL	Normal liver	No	No	Yes	Yes
HBL13	HBL13NL	Normal liver	No	No	Yes	Yes
HBL31	HBL31NL	Normal liver	No	No	Yes	Yes
HBL36	HBL36NL	Normal liver	No	No	Yes	Yes
HBL60	HBL60NL	Normal liver	No	No	Yes	Yes
Adult01	NLDNA01	Total DNA product of an adult's normal liver	No	No	Yes	No
Adult02	NLDNA02	Total DNA product of an adult's normal liver	No	No	Yes	No
Adult03	NLRNA01	Total RNA product of an adult's normal liver	No	No	No	Yes
Adult04	NLRNA02	Total RNA product of an adult's normal liver	No	No	No	Yes
Adult05	NLRNA03	Total RNA product of an adult's normal liver	No	No	No	Yes
HepG2	HepG2	Hepatoblastoma cell line	Yes	Yes	Yes	Yes
HuH6	HuH6	Hepatoblastoma cell line	Yes	Yes	Yes	Yes

Supplementary Table 2. Clinical characteristics of 59 hepatoblastoma cases.

Sample ID	Sex	Age at diagnosis (years)	AFP at diagnosis (ng/mL)	PRETEXT	Metastasis at diagnosis	Histology	Epithelial component	Liver transplantation	Refractory or relapse	EFS (years)	Survival	OS (years)
HBL01P	M	0.98	27,000	III	Yes	Embryonal type	Embryonal	No	Yes	3.24	Dead	5.23
HBL02P	F	0.98	580,000	II	Yes	Combined fetal and embryonal type	Combined fetal and embryonal	Yes	Yes	1.41	Dead	3.54
HBL03P	F	2.23	1,129,717	III	No	Combined fetal and embryonal type	Combined fetal and embryonal	Yes	Yes	1	Alive	3.83
HBL04P	M	9.07	920,000	IV	Yes	Embryonal type	Embryonal	Yes	Yes	0.41	Dead	1.89
HBL05P	M	0	1,000,000	III	No	Combined fetal and embryonal type	Combined fetal and embryonal	No	Yes	0	Dead	1.35
HBL06P	F	0.03	328,700	II	No	Combined fetal and embryonal type	Combined fetal and embryonal	No	No	11.02	Alive	11.02
HBL07P	M	0.11	525,480	II	No	Fetal type	Fetal	No	No	4.6	Alive	4.6
HBL08P	F	0.34	2,329,300	III	No	Combined fetal and embryonal type	Combined fetal and embryonal	No	No	7.2	Alive	7.2
HBL09P	F	0.39	1,200	II	No	Mixed epithelial and mesenchymal type	Combined fetal and embryonal	No	Yes	0	Dead	0
HBL10P	F	0.53	2,000,000	II	No	Mixed epithelial and mesenchymal type	Combined fetal and embryonal	No	No	7.39	Alive	7.39
HBL11P	F	0.56	1,040,000	IV	No	Combined fetal and embryonal type with macrotrabecullar type	Combined fetal and embryonal	No	No	5.76	Alive	5.76
HBL12P	F	0.66	195,000	IV	No	Mixed epithelial and mesenchymal type, mesenchymal subtype	Combined fetal and embryonal	Yes	No	6.96	Alive	6.96
HBL13P	F	0.7	3,417,350	IV	No	Combined fetal and embryonal type	Combined fetal and embryonal	No	No	0.98	Alive	0.98
HBL14P	F	0.77	229,730	I	No	Fetal type	Fetal	No	No	1.63	Alive	1.63
HBL15P	F	0.95	313,650	III	No	Combined fetal and embryonal type	Combined fetal and embryonal	No	No	17.23	Alive	19.22
HBL16P	M	1.02	49,441	II	No	Fetal type	Fetal	No	No	5.58	Alive	5.58
HBL17P	M	1.1	149,540	III	No	Fetal type, well-differentiated subtype	Fetal	No	No	10.21	Alive	10.21
HBL18P	M	1.15	5,322,006	III	Yes	Combined fetal and embryonal type	Combined fetal and embryonal	No	No	2.51	Alive	2.51
HBL19P	M	1.23	240,240	III	Yes	Embryonal type	Embryonal	No	Yes	0	Dead	1.49
HBL20P	M	1.38	370,000	III	No	Combined fetal and embryonal type	Combined fetal and embryonal	No	Yes	0	Dead	0.56
HBL21P	M	1.46	260,360	IV	No	Combined fetal and embryonal type	Combined fetal and embryonal	Yes	No	5.75	Alive	5.75
HBL22P	F	1.49	419,173	IV	No	Fetal type, Mitotically active subtype	Fetal	No	Yes	1.32	Alive	2.56
HBL23P	F	1.5	520,000	II	No	Mixed epithelial and mesenchymal type	Combined fetal and embryonal	No	No	4.06	Alive	4.06
HBL24P	F	1.53	2,238	II	No	Combined fetal and embryonal type	Combined fetal and embryonal	No	No	3.32	Alive	3.32
HBL25P	M	1.61	105,648	III	No	Combined fetal and embryonal type	Combined fetal and embryonal	No	No	11.45	Alive	11.45
HBL26P	F	1.69	259,486	III	No	Mixed epithelial and mesenchymal type, simple subtype	Combined fetal and embryonal	No	No	3.07	Alive	3.07
HBL27P	M	1.71	155,274	IV	No	Combined fetal and embryonal type	Combined fetal and embryonal	Yes	No	0.4	Alive	0.4
HBL28P	F	2.19	2,203,300	IV	Yes	Mixed epithelial and mesenchymal type, simple subtype	Combined fetal and embryonal	Yes	No	1.36	Alive	1.36
HBL29P	M	2.34	3,765,600	IV	Yes	Combined fetal and embryonal type	Combined fetal and embryonal	Yes	Yes	1.67	Alive	1.69
HBL30P	M	2.35	58,045	I	No	Combined fetal and embryonal type	Combined fetal and embryonal	No	No	3.76	Alive	3.76
HBL31P	M	2.36	551	II	No	Fetal type, well-differentiated subtype	Fetal	No	No	3.78	Alive	3.78
HBL32P	F	2.63	49,843	IV	No	Combined fetal and embryonal type	Combined fetal and embryonal	Yes	No	4.07	Alive	4.07
HBL33P	F	2.85	189,071	III	No	Combined fetal and embryonal type	Combined fetal and embryonal	No	No	6.84	Alive	6.84
HBL34P	M	3.22	1,000,000	IV	Yes	Combined fetal and embryonal type	Combined fetal and embryonal	Yes	Yes	0.83	Dead	2.09
HBL35P	M	3.97	1,792	II	No	Fetal type, Mitotically active subtype	Fetal	No	No	3.16	Alive	3.16
HBL36P	M	7.09	977,800	III	No	Embryonal type	Embryonal	No	No	12.82	Alive	12.82
HBL37P	M	7.29	178,868	III	No	Combined fetal and embryonal type	Combined fetal and embryonal	No	No	8.04	Alive	8.04
HBL38P	F	8.21	441	IV	No	Fetal type	Fetal	No	No	8.67	Alive	8.67
HBL39P	M	11.03	24,714	IV	No	Fetal type	Fetal	No	No	10.35	Alive	10.35
HBL40C	F	0	274,200	II	No	Fetal Type	Fetal	No	No	0.38	Alive	0.38
HBL41C	F	0.18	470,000	III	No	Specific epithelial type	Fetal	No	No	15.78	Alive	15.78
HBL42C	F	0.25	1,462,050	IV	No	Combined fetal and embryonal type	Combined fetal and embryonal	Yes	Yes	0.65	Dead	1.12
HBL43C	M	0.27	229,008	III	No	Mixed epithelial and mesenchymal type	Fetal	No	No	17.71	Alive	17.71

HBL44C	M	0.3	52,690	III	Yes	Mixed epithelial and mesenchymal type, simple subtype	Combined fetal and embryonal	No	Yes	0.41	Alive	2.28
HBL45C	F	0.37	150,000	IV	No	Fetal type	Fetal	Yes	Yes	0.72	Alive	3.15
HBL46C	F	0.46	300,000	II	No	Combined fetal and embryonal type	Combined fetal and embryonal	No	No	0.36	Alive	0.36
HBL47C	M	0.53	1,801,200	IV	No	Mixed epithelial and mesenchymal type	Combined fetal and embryonal	Yes	Yes	11	Alive	12.45
HBL48C	M	0.83	540,000	III	No	Mixed epithelial and mesenchymal type, teratoid subtype	Combined fetal and embryonal	No	No	7.1	Alive	7.1
HBL49C	M	0.88	30,829	III	No	Fetal type, well-differentiated subtype, if primary	Fetal	No	No	0.6	Alive	0.87
HBL50C	F	1.02	1,714,150	III	No	Combined fetal and embryonal type	Combined fetal and embryonal	No	No	3.89	Alive	3.89
HBL51C	F	1.28	304,581	III	No	Combined fetal and embryonal type	Combined fetal and embryonal	No	No	8.47	Alive	8.47
HBL52C	M	1.39	121,900	IV	No	Combined fetal and embryonal type	Combined fetal and embryonal	Yes	No	7.17	Alive	7.17
HBL53C	F	1.48	37,770	II	No	Combined fetal and embryonal type	Combined fetal and embryonal	No	No	11.85	Alive	11.85
HBL54C	F	1.75	318,695	III	No	Combined fetal and embryonal type	Combined fetal and embryonal	Yes	No	3.83	Alive	3.83
HBL55C	F	1.98	945,000	II	Yes	Mixed epithelial and mesenchymal type, teratoid subtype, if primary	Combined fetal and embryonal	No	Yes	1.41	Alive	8.45
HBL56C	M	3.41	512,000	IV	Yes	Histology unclear due to modification	Unclear	No	No	3.09	Alive	3.09
HBL57C	M	3.49	1,307,800	IV	Yes	Mixed epithelial and mesenchymal type, teratoid subtype	Combined fetal and embryonal	Yes	No	2.15	Alive	2.15
HBL58C	F	7.99	610,000	III	No	Embryonal type	Embryonal	No	No	8.23	Alive	8.23
HBL59C	M	10.25	234,600	III	Yes	Combined fetal and embryonal type	Combined	No	No	0.72	Alive	0.72

F, female; M, male; AFP, alpha-fetoprotein; PRETEXT, pretreatment extent of tumor; EFS, event-free survival; OS, overall survival.

Supplementary Table 3. Somatic alterations and APC germline truncating mutations detected in Genomon mutation call.

Sample	Gene name	Mutation type	Germline or somatic	Chr	Start	End	Reference	Alteration	Amino acid change	Read depth	VAF	EB
HBL03P	*APC*	Frameshift deletion	Germline	5	112174597	112174597	C	-	NM_001127511:p.Y1084fs	1363	0.564	60
HBL12P	*APC*	Frameshift deletion	Germline	5	112175212	112175216	AAAAG	-	NM_001127511:p.E1291fs	830	0.407	60
HBL45C	*APC*	Frameshift deletion	Germline	5	112175676	112175677	AG	-	NM_001127511:p.S1447fs	881	0.726	60
HBL49C	*APC*	Nonsense mutation	Germline	5	112174286	112174286	C	T	NM_001127511:p.Q981X	572	0.476	60
HBL03P	*APC*	Splice site mutation	Somatic	5	112151191	112151191	G	A	-	727	0.239	60
HBL12P	*APC*	Frameshift insertion	Somatic	5	112175749	112175749	-	A	NM_001127511:p.T1469fs	1008	0.344	60
HBL48C	*BAGE3*	Missense mutation	Somatic	21	11049581	11049581	T	C	NM_182481:p.E107G	1680	0.068	60
HBL58C	*BAP1*	Missense mutation	Somatic	3	52442570	52442570	G	A	NM_004656:p.R59W	576	0.252	60
HBL37P	*BCOR*	Missense mutation	Somatic	X	39921444	39921444	T	C	NM_001123384:p.N1407S	119	0.58	60
HBL37P	*BCORL1*	Frameshift deletion	Somatic	X	129148635	129148635	C	-	NM_001184772:p.R630fs	596	0.554	60
HBL26P	*CRLF2*	Frameshift insertion	Somatic	X	1317543	1317543	-	CCTTCTAT	NM_001012288:p.L63X	1353	0.318	60
HBL01P	*CTNNB1*	Missense mutation	Somatic	3	41266125	41266125	C	T	NM_001098209:p.T41I	772	0.304	60
HBL02P	*CTNNB1*	Missense mutation	Somatic	3	41266101	41266101	C	A	NM_001098209:p.S33Y	660	0.274	60
HBL04P	*CTNNB1*	Non-frameshift deletion	Somatic	3	41266106	41266120	ATCCATTCTGGTGCC	-	NM_001098209:p.35_39del	968	0.221	60
HBL05P	*CTNNB1*	Missense mutation	Somatic	3	41266104	41266104	G	T	NM_001098209:p.G34V	829	0.331	60
HBL06P	*CTNNB1*	Non-frameshift deletion	Somatic	3	41266060	41266101	AGCGGCTGTTAGTCACTGGCAG CAACAGTCTTACCTGGACTCT	G	NM_001098209:p.20_33del	996	0.088	60
HBL09P	*CTNNB1*	Missense mutation	Somatic	3	41266104	41266104	G	T	NM_001098209:p.G34V	693	0.257	60
HBL10P	*CTNNB1*	Missense mutation	Somatic	3	41266124	41266124	A	G	NM_001098209:p.T41A	543	0.407	60
HBL11P	*CTNNB1*	Missense mutation	Somatic	3	41266097	41266097	G	A	NM_001098209:p.D32N	667	0.247	60
HBL13P	*CTNNB1*	Missense mutation	Somatic	3	41266104	41266104	G	T	NM_001098209:p.G34V	642	0.171	60
HBL16P	*CTNNB1*	Non-frameshift deletion	Somatic	3	41266069	41266098	TAGTCACTGGCAGCAACAGTCTT ACCTGGA	-	NM_001098209:p.23_32del	881	0.227	60
HBL18P	*CTNNB1*	Missense mutation	Somatic	3	41266104	41266104	G	T	NM_001098209:p.G34V	890	0.122	60
HBL19P	*CTNNB1*	Missense mutation	Somatic	3	41266104	41266104	G	T	NM_001098209:p.G34V	752	0.42	60
HBL20P	*CTNNB1*	Missense mutation	Somatic	3	41266136	41266136	T	C	NM_001098209:p.S45P	764	0.151	60
HBL21P	*CTNNB1*	Missense mutation	Somatic	3	41266097	41266097	G	T	NM_001098209:p.D32Y	795	0.409	60
HBL22P	*CTNNB1*	Non-frameshift deletion	Somatic	3	41266071	41266109	GTCACTGGCAGCAACAGTCTTA CCTGGACTCTGGAATCC	-	NM_001098209:p.23_36del insN	902	0.146	60
HBL25P	*CTNNB1*	Missense mutation	Somatic	3	41266104	41266104	G	T	NM_001098209:p.G34V	674	0.166	60
HBL27P	*CTNNB1*	Non-frameshift deletion	Somatic	3	41266072	41266101	TCACTGGCAGCAACAGTCTTACC TGGACTC	-	NM_001098209:p.24_33del	1191	0.053	60
HBL31P	*CTNNB1*	Non-frameshift deletion	Somatic	3	41266069	41266116	TAGTCACTGGCAGCAACAGTCTT ACCTGGACTCTGGAATCCATTCT GG	-	NM_001098209:p.23_38del	946	0.052	60
HBL34P	*CTNNB1*	Missense mutation	Somatic	3	41266103	41266103	G	A	NM_001098209:p.G34R	706	0.147	60
HBL35P	*CTNNB1*	Missense mutation	Somatic	3	41266104	41266104	G	A	NM_001098209:p.G34E	762	0.365	60
HBL37P	*CTNNB1*	Missense mutation	Somatic	3	41266097	41266097	G	A	NM_001098209:p.D32N	611	0.303	60
HBL41C	*CTNNB1*	Missense mutation	Somatic	3	41266097	41266097	G	T	NM_001098209:p.D32Y	533	0.229	60
HBL42C	*CTNNB1*	Missense mutation	Somatic	3	41266097	41266097	G	A	NM_001098209:p.D32N	612	0.422	60
HBL44C	*CTNNB1*	Missense mutation	Somatic	3	41266124	41266124	A	G	NM_001098209:p.T41A	889	0.416	60
HBL47C	*CTNNB1*	Missense mutation	Somatic	3	41266103	41266103	G	A	NM_001098209:p.G34R	794	0.316	60
HBL48C	*CTNNB1*	Missense mutation	Somatic	3	41266125	41266125	C	T	NM_001098209:p.T41I	609	0.447	60
HBL53C	*CTNNB1*	Missense mutation	Somatic	3	41266097	41266097	G	T	NM_001098209:p.D32Y	675	0.2	60
HBL01P	*DST*	Missense mutation	Somatic	6	56515786	56515786	C	T	NM_183380:p.V247M	957	0.226	60
HBL04P	*DST*	Nonsense mutation	Somatic	6	56341124	56341124	C	T	NM_015548:p.W4606X	622	0.178	60
HBL52C	*EPHB1*	Missense mutation	Somatic	3	134977951	134977951	G	T	NM_004441:p.A982S	678	0.112	60
HBL59C	*KDM6A*	Frameshift deletion	Somatic	X	44941994	44941995	AT	-	NM_001291418:p.I1003fs	397	0.554	60
HBL29P	*KMT2C*	Missense mutation	Somatic	7	151945124	151945124	T	C	NM_170606:p.N799D	2644	0.083	60

HBL04P	*PEG10*	Missense mutation	Somatic	7	94293733	94293733	C	A	NM_001040152:p.R289S	344	0.203	60
HBL37P	*PEG10*	Frameshift insertion	Somatic	7	94293050	94293050	-	C	NM_001040152:p.P62fs	814	0.273	60
HBL37P	*PTPRD*	Missense mutation	Somatic	9	8492964	8492964	C	T	NM_002839:p.G789R	684	0.323	60
HBL26P	*PTPRO*	Missense mutation	Somatic	12	15636936	15636936	A	C	NM_002848:p.Q35P	685	0.247	60
HBL51C	*PTPRO*	Frameshift insertion	Somatic	12	15699537	15699537	-	A	NM_002848:p.T735fs	823	0.051	60
HBL37P	*TRIM60*	Missense mutation	Somatic	4	165961832	165961832	G	A	NM_152620:p.R203K	546	0.308	60
HBL35P	*TERT*	Promoter mutation	Somatic	5	1295228	1295228	G	A	-	9	0.556	4.983
HBL39P	*TERT*	Promoter mutation	Somatic	5	1295228	1295228	G	A	-	11	0.455	4.684
HBL58C	*ZMYM3*	Missense mutation	Somatic	X	70461158	70461158	G	A	NM_001171162:p.A1268V	602	0.229	60

Chr, Chromosome; VAF, Variant allele frequency; EB, EB Call *P* value (-log10).

Supplementary Table 4. Structural variations detected in Genomon SV call.

Sample	Chromosome 1	Position 1	Direction 1	Chromosome 2	Position 2	Direction 2	Insert	Variant type	Gene 1	Gene 2	Read-pair number of reference	Read-pair number of variant	Variant allele frequency	Maximum overhang 1	Maximum overhang 2
HBL06P	3	41266059	+	3	41266103	-	G	Deletion	*CTNNB1*	*CTNNB1*	459	209	0.3129	483	262
HBL07P	3	41264122	-	3	41266082	+	---	Tandem duplication	*CTNNB1*	*CTNNB1*	563	167	0.2288	261	320
HBL07P	3	41264836	+	3	41266339	-	GTT	Deletion	*CTNNB1*	*CTNNB1*	444	89	0.167	262	323
HBL08P	3	41265906	+	3	41266235	-	---	Deletion	*CTNNB1*	*CTNNB1*	713	95	0.1176	381	319
HBL14P	3	41265996	+	3	41266249	-	---	Deletion	*CTNNB1*	*CTNNB1*	724	148	0.1697	340	566
HBL15P	3	41265732	+	3	41266226	-	---	Deletion	*CTNNB1*	*CTNNB1*	783	258	0.2478	412	382
HBL16P	3	41266068	+	3	41266099	-	---	Deletion	*CTNNB1*	*CTNNB1*	362	212	0.3693	394	229
HBL17P	3	41266067	+	3	41266125	-	GTT	Deletion	*CTNNB1*	*CTNNB1*	601	99	0.1414	232	287
HBL22P	3	41266070	+	3	41266110	-	---	Deletion	*CTNNB1*	*CTNNB1*	379	201	0.3466	392	307
HBL23P	3	41266095	+	3	41266255	-	---	Deletion	*CTNNB1*	*CTNNB1*	720	94	0.1155	369	273
HBL24P	3	41265565	+	3	41266627	-	---	Deletion	*CTNNB1*	*CTNNB1*	1057	241	0.1857	348	332
HBL26P	3	41265859	+	3	41266420	-	---	Deletion	*CTNNB1*	*CTNNB1*	1405	192	0.1202	379	278
HBL27P	3	41266071	+	3	41266102	-	---	Deletion	*CTNNB1*	*CTNNB1*	749	64	0.0787	256	173
HBL28P	3	41265996	+	3	41266263	-	---	Deletion	*CTNNB1*	*CTNNB1*	673	178	0.2092	301	392
HBL29P	3	41266055	+	3	41266264	-	---	Deletion	*CTNNB1*	*CTNNB1*	627	182	0.225	319	405
HBL30P	3	41265919	+	3	41266309	-	---	Deletion	*CTNNB1*	*CTNNB1*	864	296	0.2552	321	350
HBL31P	3	41266069	+	3	41266118	-	---	Deletion	*CTNNB1*	*CTNNB1*	454	157	0.257	382	375
HBL32P	3	41266072	+	3	41266270	-	---	Deletion	*CTNNB1*	*CTNNB1*	518	229	0.3066	296	403
HBL33P	3	41265716	+	3	41266125	-	---	Deletion	*CTNNB1*	*CTNNB1*	564	186	0.248	347	420
HBL36P	3	41265987	+	3	41266328	-	---	Deletion	*CTNNB1*	*CTNNB1*	847	248	0.2265	355	419
HBL38P	3	41266022	+	3	41266622	-	---	Deletion	*CTNNB1*	*CTNNB1*	1502	88	0.0553	331	371
HBL39P	3	41265567	+	3	41266629	-	---	Deletion	*CTNNB1*	*CTNNB1*	1096	333	0.233	290	339
HBL40C	3	41265937	+	3	41266328	-	---	Deletion	*CTNNB1*	*CTNNB1*	724	145	0.1669	298	354
HBL43C	3	41265953	+	3	41266354	-	---	Deletion	*CTNNB1*	*CTNNB1*	918	148	0.1388	390	310
HBL46C	3	41265638	+	3	41266246	-	---	Deletion	*CTNNB1*	*CTNNB1*	1170	67	0.0542	319	172
HBL51C	3	41266050	+	3	41266231	-	---	Deletion	*CTNNB1*	*CTNNB1*	529	121	0.1862	363	313
HBL52C	3	41265971	+	3	41266332	-	---	Deletion	*CTNNB1*	*CTNNB1*	547	173	0.2403	310	298
HBL54C	3	41265890	+	3	41266310	-	---	Deletion	*CTNNB1*	*CTNNB1*	1192	27	0.0221	259	214
HBL55C	3	41265646	+	3	41266230	-	---	Deletion	*CTNNB1*	*CTNNB1*	767	127	0.1421	291	465
HBL56C	3	41266072	+	3	41266113	-	GCCGCTACCACAG	Deletion	*CTNNB1*	*CTNNB1*	729	109	0.1301	346	356
HBL57C	3	41266093	+	3	41266273	-	---	Deletion	*CTNNB1*	*CTNNB1*	833	87	0.0946	270	230
HBL58C	3	41266068	+	3	41266123	-	---	Deletion	*CTNNB1*	*CTNNB1*	659	82	0.1107	428	309
HBL59C	3	41266039	+	3	41266636	-	---	Deletion	*CTNNB1*	*CTNNB1*	1177	213	0.1532	288	277

Supplementary Table 5. Number of somatic alterations in each gene targeted in capture sequencing.

Gene	Number of somatic alterations	Frequency (%)	Types of alterations
CTNNB1	55	93	Missense mutation, inframe deletion
APC	2	3	Frameshift insertion, Splice site mutation
DST	2	3	Missense mutation, Nonsense mutation
PEG10	2	3	Missense mutation, Frameshift insertion
PTPRO	2	3	Missense mutation, Frameshift insertion
TERT (promoter)	2	3	Promoter activating mutation
BAGE3	1	2	Missense mutation
BAP1	1	2	Missense mutation
BCOR	1	2	Missense mutation
BCORL1	1	2	Frameshift deletion
CRLF2	1	2	Frameshift insertion
EPHB1	1	2	Missense mutation
KDM6A	1	2	Frameshift deletion
KMT2C	1	2	Missense mutation
PTPRD	1	2	Missense mutation
TRIM60	1	2	Missense mutation
ZMYM3	1	2	Missense mutation
ABL1	0	0	-
ABL2	0	0	-
AFF3	0	0	-
AKT1	0	0	-
AKT2	0	0	-
AKT3	0	0	-
ALK	0	0	-
AMER1	0	0	-
AR	0	0	-
ARHGAP35	0	0	-
ARID1A	0	0	-
ARID1B	0	0	-
ARID2	0	0	-
ASXL1	0	0	-
ATM	0	0	-
ATR	0	0	-
ATRX	0	0	-
AURKA	0	0	-
AURKB	0	0	-
AXIN1	0	0	-
AXIN2	0	0	-
AXL	0	0	-
BAK1	0	0	-
BARD1	0	0	-
BCL10	0	0	-
BCL2	0	0	-
BCL2L1	0	0	-
BCL2L2	0	0	-
BCL6	0	0	-
BIRC3	0	0	-
BLM	0	0	-
BRAF	0	0	-
BRCA1	0	0	-
BRCA2	0	0	-
BRIP1	0	0	-
BTK	0	0	-
BUB1B	0	0	-
CAPRIN2	0	0	-
CARD11	0	0	-
CASP8	0	0	-
CBFB	0	0	-
CBL	0	0	-
CBLB	0	0	-
CCND1	0	0	-
CCND2	0	0	-
CCND3	0	0	-
CCNE1	0	0	-
CD274	0	0	-
CD79A	0	0	-
CD79B	0	0	-
CDC27	0	0	-
CDC73	0	0	-
CDH1	0	0	-
CDK12	0	0	-
CDK4	0	0	-
CDK6	0	0	-
CDK8	0	0	-

CDKN1A	0	0	-
CDKN1B	0	0	-
CDKN2A	0	0	-
CDKN2B	0	0	-
CDKN2C	0	0	-
CEBPA	0	0	-
CHEK1	0	0	-
CHEK2	0	0	-
CIC	0	0	-
COL2A1	0	0	-
COL5A1	0	0	-
COL5A2	0	0	-
CREBBP	0	0	-
CRKL	0	0	-
CSF1R	0	0	-
CSMD1	0	0	-
CSMD2	0	0	-
CSMD3	0	0	-
CTCF	0	0	-
CTNNA1	0	0	-
CUL3	0	0	-
CYLD	0	0	-
DAXX	0	0	-
DDB2	0	0	-
DDR2	0	0	-
DICER1	0	0	-
DKK1	0	0	-
DKK2	0	0	-
DKK3	0	0	-
DKK4	0	0	-
DNMT3A	0	0	-
DPPA3	0	0	-
DROSHA	0	0	-
ECT2L	0	0	-
EGFR	0	0	-
EP300	0	0	-
EPCAM	0	0	-
EPHA3	0	0	-
EPHA7	0	0	-
ERBB2	0	0	-
ERBB3	0	0	-
ERBB4	0	0	-
ERCC5	0	0	-
ERG	0	0	-
ESR1	0	0	-
ETV1	0	0	-
ETV4	0	0	-
EWSR1	0	0	-
EZH2	0	0	-
FAM46C	0	0	-
FANCA	0	0	-
FANCC	0	0	-
FANCD2	0	0	-
FANCE	0	0	-
FANCF	0	0	-
FANCG	0	0	-
FAS	0	0	-
FAT1	0	0	-
FBXO11	0	0	-
FBXW7	0	0	-
FEV	0	0	-
FGF19	0	0	-
FGF3	0	0	-
FGF4	0	0	-
FGFR1	0	0	-
FGFR2	0	0	-
FGFR3	0	0	-
FGFR4	0	0	-
FH	0	0	-
FLCN	0	0	-
FLI1	0	0	-
FLT1	0	0	-
FLT3	0	0	-
FLT4	0	0	-
FOXL2	0	0	-
FOXO1	0	0	-
FOXP1	0	0	-
FUBP1	0	0	-
FUS	0	0	-

FZD10	0	0	-
FZD3	0	0	-
GAB1	0	0	-
GATA1	0	0	-
GATA2	0	0	-
GATA3	0	0	-
GLI1	0	0	-
GNA11	0	0	-
GNAQ	0	0	-
GNAS	0	0	-
GPC3	0	0	-
GRB2	0	0	-
GRIN2A	0	0	-
H3F3A	0	0	-
H3F3B	0	0	-
HIST1H3B	0	0	-
HNF1A	0	0	-
HRAS	0	0	-
HSPH1	0	0	-
IDH1	0	0	-
IDH2	0	0	-
IGF1R	0	0	-
IGF2	0	0	-
IGF2R	0	0	-
IKBKE	0	0	-
IKZF1	0	0	-
IL6ST	0	0	-
IL7R	0	0	-
INO80D	0	0	-
IRF4	0	0	-
IRS2	0	0	-
JAK1	0	0	-
JAK2	0	0	-
JAK3	0	0	-
JMJD1C	0	0	-
JUN	0	0	-
KDM5C	0	0	-
KDR	0	0	-
KEAP1	0	0	-
KIT	0	0	-
KLF4	0	0	-
KLF6	0	0	-
KMT2A	0	0	-
KMT2B	0	0	-
KMT2D	0	0	-
KRAS	0	0	-
LGR5	0	0	-
LIN28A	0	0	-
LIN28B	0	0	-
LMO1	0	0	-
LRP1	0	0	-
LRP1B	0	0	-
LRP5	0	0	-
MAP2K1	0	0	-
MAP2K2	0	0	-
MAP2K4	0	0	-
MAP3K1	0	0	-
MAP4K3	0	0	-
MAPK1	0	0	-
MAPK3	0	0	-
MCL1	0	0	-
MDM2	0	0	-
MDM4	0	0	-
MED12	0	0	-
MEN1	0	0	-
MET	0	0	-
MITF	0	0	-
MLH1	0	0	-
MRE11A	0	0	-
MSH2	0	0	-
MSH6	0	0	-
MTMR4	0	0	-
MTOR	0	0	-
MUC16	0	0	-
MUC4	0	0	-
MUTYH	0	0	-
MYC	0	0	-
MYCL1	0	0	-
MYCN	0	0	-

MYD88	0	0	-
MYH2	0	0	-
MYOD1	0	0	-
NANOG	0	0	-
NBN	0	0	-
NCOA1	0	0	-
NCOA2	0	0	-
NES	0	0	-
NF1	0	0	-
NF2	0	0	-
NFE2L2	0	0	-
NFKBIA	0	0	-
NKX2-1	0	0	-
NOTCH1	0	0	-
NOTCH2	0	0	-
NOTCH3	0	0	-
NOTCH4	0	0	-
NPM1	0	0	-
NRAS	0	0	-
NSD1	0	0	-
NTRK1	0	0	-
NTRK3	0	0	-
OBSCN	0	0	-
PALB2	0	0	-
PARK2	0	0	-
PARP1	0	0	-
PAX3	0	0	-
PAX5	0	0	-
PAX7	0	0	-
PBRM1	0	0	-
PDCD1LG2	0	0	-
PDE11A	0	0	-
PDGFRA	0	0	-
PDGFRB	0	0	-
PHF6	0	0	-
PHOX2B	0	0	-
PIK3C2B	0	0	-
PIK3CA	0	0	-
PIK3CB	0	0	-
PIK3CD	0	0	-
PIK3CG	0	0	-
PIK3R1	0	0	-
PIK3R2	0	0	-
PINK1	0	0	-
PLAG1	0	0	-
PLVAP	0	0	-
PMS2	0	0	-
POLE	0	0	-
POU5F1	0	0	-
PPP2R1A	0	0	-
PRB2	0	0	-
PRDM1	0	0	-
PRDM9	0	0	-
PRKAR1A	0	0	-
PRKCZ	0	0	-
PRKDC	0	0	-
PRKRIR	0	0	-
PTCH1	0	0	-
PTEN	0	0	-
PTPN11	0	0	-
PTPRB	0	0	-
PTPRN	0	0	-
PTPRT	0	0	-
PZP	0	0	-
RAC1	0	0	-
RAD17	0	0	-
RAD50	0	0	-
RAD51	0	0	-
RAF1	0	0	-
RARA	0	0	-
RB1	0	0	-
RET	0	0	-
RHOA	0	0	-
ROBO1	0	0	-
ROS1	0	0	-
RSPO2	0	0	-
RUNX1	0	0	-
RUNX1T1	0	0	-
SDHA	0	0	-

SDHB	0	0	-
SDHC	0	0	-
SDHD	0	0	-
SETD2	0	0	-
SF3B1	0	0	-
SHC1	0	0	-
SHOC2	0	0	-
SLC7A8	0	0	-
SMAD2	0	0	-
SMAD3	0	0	-
SMAD4	0	0	-
SMARCA2	0	0	-
SMARCA4	0	0	-
SMARCB1	0	0	-
SMO	0	0	-
SOCS1	0	0	-
SOS1	0	0	-
SOX2	0	0	-
SOX9	0	0	-
SPEN	0	0	-
SPOP	0	0	-
SPRED1	0	0	-
SPRY4	0	0	-
SRC	0	0	-
STAG2	0	0	-
STAT3	0	0	-
STK11	0	0	-
SUFU	0	0	-
SYK	0	0	-
SYNE1	0	0	-
TCF3	0	0	-
TCF7L2	0	0	-
TET1	0	0	-
TET2	0	0	-
TET3	0	0	-
TFAP2C	0	0	-
TGFBR2	0	0	-
TIAM1	0	0	-
TNFAIP3	0	0	-
TNFRSF14	0	0	-
TOP1	0	0	-
TP53	0	0	-
TRAF7	0	0	-
TSC1	0	0	-
TSC2	0	0	-
TSHR	0	0	-
U2AF1	0	0	-
USP28	0	0	-
VHL	0	0	-
WT1	0	0	-
XPC	0	0	-
XPO1	0	0	-
XRCC2	0	0	-
ZNF2	0	0	-
ZNF518B	0	0	-
ZRSR2	0	0	-

Supplementary Table 6. KEGG pathways significantly upregulated in hepatoblastoma cluster F compared to normal liver.

KEGG pathway	Geometric mean of the individual *P* values	Mean of the individual statistics	*P* value	FDR *q* value	Set size
RIBOSOME	1.52E-05	3.450684738	8.54E-22	1.59E-19	87
ECM RECEPTOR INTERACTION	0.012225052	1.691157055	3.58E-07	3.33E-05	84
ARRHYTHMOGENIC RIGHT VENTRICULAR CARDIOMYOPATHY ARVC	0.028081981	1.646530722	5.74E-07	3.56E-05	74
BASAL CELL CARCINOMA	0.035839761	1.486342314	6.65E-06	0.000309182	55
CELL CYCLE	0.014449612	1.427645656	1.28E-05	0.000445587	125
WNT SIGNALING PATHWAY	0.056427575	1.402406773	1.44E-05	0.000445587	151

FDR, false discovery rate.

Supplementary Table 7. KEGG pathways significantly downregulated in hepatoblastoma cluster F compared to normal liver.

KEGG pathway	Geometric mean of the individual *P* values	Mean of the individual statistics	*P* value	FDR *q* value	Set size
RETINOL METABOLISM	3.50E-08	-5.956666715	1.12E-58	2.08E-56	64
DRUG METABOLISM CYTOCHROME P450	5.98E-08	-5.712500194	1.51E-55	1.41E-53	72
METABOLISM OF XENOBIOTICS BY CYTOCHROME P450	3.50E-07	-5.280199209	8.86E-49	5.49E-47	70
DRUG METABOLISM OTHER ENZYMES	4.81E-07	-5.420655055	1.55E-48	7.21E-47	51
STEROID HORMONE BIOSYNTHESIS	6.18E-06	-4.675814721	7.24E-39	2.69E-37	55
COMPLEMENT AND COAGULATION CASCADES	6.32E-06	-4.504154522	3.77E-38	1.17E-36	69
STARCH AND SUCROSE METABOLISM	8.60E-06	-4.600799859	1.17E-37	3.10E-36	52
CYTOKINE CYTOKINE RECEPTOR INTERACTION	2.74E-06	-4.094499541	6.44E-34	1.50E-32	264
ASCORBATE AND ALDARATE METABOLISM	2.88E-05	-4.625198383	1.65E-32	3.40E-31	25
PENTOSE AND GLUCURONATE INTERCONVERSIONS	5.49E-05	-4.327259181	2.95E-30	5.48E-29	28
PORPHYRIN AND CHLOROPHYLL METABOLISM	4.69E-05	-4.133367596	3.41E-30	5.77E-29	41
GLYCINE SERINE AND THREONINE METABOLISM	9.41E-05	-3.951747861	3.26E-28	5.05E-27	31
TRYPTOPHAN METABOLISM	0.000138052	-3.839260713	1.02E-27	1.46E-26	40
VALINE LEUCINE AND ISOLEUCINE DEGRADATION	0.000168983	-3.628566898	3.27E-25	4.35E-24	44
GLYCOLYSIS GLUCONEOGENESIS	0.00069124	-3.243062668	1.32E-21	1.63E-20	62
JAK STAT SIGNALING PATHWAY	0.000822718	-3.119468758	9.58E-21	1.11E-19	154
PROPANOATE METABOLISM	0.002076386	-2.90875689	4.11E-17	4.50E-16	32
ARACHIDONIC ACID METABOLISM	0.003618565	-2.697203281	1.62E-15	1.68E-14	58
PARKINSONS DISEASE	0.002709206	-2.570487175	1.78E-14	1.74E-13	112
PRION DISEASES	0.005124588	-2.623608369	2.18E-14	2.03E-13	35
LINOLEIC ACID METABOLISM	0.005928484	-2.644127996	2.58E-14	2.28E-13	29
NOD LIKE RECEPTOR SIGNALING PATHWAY	0.004891012	-2.53784824	4.04E-14	3.42E-13	62
HISTIDINE METABOLISM	0.005862899	-2.608964196	4.57E-14	3.69E-13	28
CHEMOKINE SIGNALING PATHWAY	0.001602439	-2.471267485	1.19E-13	9.26E-13	189
FATTY ACID METABOLISM	0.006456201	-2.469516951	3.15E-13	2.34E-12	42
TYROSINE METABOLISM	0.00822277	-2.422117807	7.43E-13	5.32E-12	41
PYRUVATE METABOLISM	0.008942383	-2.365665328	2.19E-12	1.51E-11	40
PEROXISOME	0.005858699	-2.346454391	2.50E-12	1.66E-11	78
BETA ALANINE METABOLISM	0.011873657	-2.345647736	9.93E-12	6.37E-11	22
TOLL LIKE RECEPTOR SIGNALING PATHWAY	0.008089637	-2.248515812	1.31E-11	8.15E-11	101
APOPTOSIS	0.009835886	-2.227547534	2.09E-11	1.26E-10	87
HUNTINGTONS DISEASE	0.007914522	-2.198426651	3.06E-11	1.78E-10	172
CITRATE CYCLE TCA CYCLE	0.011194007	-2.22138434	9.01E-11	5.08E-10	30
BUTANOATE METABOLISM	0.015385688	-2.167537096	1.13E-10	6.17E-10	34
CYSTEINE AND METHIONINE METABOLISM	0.016198178	-2.159952506	1.50E-10	7.95E-10	34
LEISHMANIA INFECTION	0.004696042	-2.103988507	4.44E-10	2.30E-09	70
GLUTATHIONE METABOLISM	0.014067634	-2.065594098	7.32E-10	3.68E-09	50
B CELL RECEPTOR SIGNALING PATHWAY	0.01437473	-2.037331614	8.28E-10	4.05E-09	75
PRIMARY BILE ACID BIOSYNTHESIS	0.020892053	-2.141223731	9.33E-10	4.45E-09	16
OXIDATIVE PHOSPHORYLATION	0.007207578	-2.029851955	1.16E-09	5.38E-09	116
GRAFT VERSUS HOST DISEASE	0.005887693	-2.090404847	1.39E-09	6.30E-09	39
FRUCTOSE AND MANNOSE METABOLISM	0.022193095	-2.010227068	1.85E-09	8.18E-09	34
SELENOAMINO ACID METABOLISM	0.027917879	-1.920946531	1.04E-08	4.49E-08	25
PHENYLALANINE METABOLISM	0.026580755	-1.951730396	1.07E-08	4.53E-08	18
ALZHEIMERS DISEASE	0.030096722	-1.767260501	6.65E-08	2.75E-07	157
LYSINE DEGRADATION	0.036964937	-1.686056883	3.38E-07	1.37E-06	44
NATURAL KILLER CELL MEDIATED CYTOTOXICITY	0.022435539	-1.665486187	3.73E-07	1.47E-06	136
CYTOSOLIC DNA SENSING PATHWAY	0.039670411	-1.660860094	4.35E-07	1.69E-06	55
INTESTINAL IMMUNE NETWORK FOR IGA PRODUCTION	0.014604497	-1.700450209	4.78E-07	1.81E-06	46
FOLATE BIOSYNTHESIS	0.053032	-1.683647448	8.00E-07	2.98E-06	11
ARGININE AND PROLINE METABOLISM	0.052399826	-1.585124517	1.30E-06	4.75E-06	53
ALLOGRAFT REJECTION	0.010998444	-1.645187484	2.77E-06	9.89E-06	35
NICOTINATE AND NICOTINAMIDE METABOLISM	0.063502254	-1.547740881	2.90E-06	1.02E-05	24
HEMATOPOIETIC CELL LINEAGE	0.018162472	-1.539107508	3.03E-06	1.04E-05	85
GALACTOSE METABOLISM	0.063717347	-1.513686908	4.20E-06	1.42E-05	26
PYRIMIDINE METABOLISM	0.029722349	-1.499360819	4.52E-06	1.50E-05	98
PENTOSE PHOSPHATE PATHWAY	0.062004367	-1.502876684	4.95E-06	1.61E-05	27
ABC TRANSPORTERS	0.062028545	-1.483570946	5.69E-06	1.83E-05	44
LIMONENE AND PINENE DEGRADATION	0.061866339	-1.555734645	7.61E-06	2.40E-05	10
GLYCEROLIPID METABOLISM	0.068324017	-1.454674889	7.78E-06	2.41E-05	49
GLYOXYLATE AND DICARBOXYLATE METABOLISM	0.073158643	-1.480849221	9.38E-06	2.83E-05	16
MATURITY ONSET DIABETES OF THE YOUNG	0.07156282	-1.453845719	9.45E-06	2.83E-05	25
AMINO SUGAR AND NUCLEOTIDE SUGAR METABOLISM	0.060080047	-1.431603291	1.15E-05	3.41E-05	44
ALANINE ASPARTATE AND GLUTAMATE METABOLISM	0.072452584	-1.421278622	1.41E-05	4.10E-05	32
INSULIN SIGNALING PATHWAY	0.065657048	-1.396721134	1.50E-05	4.30E-05	137
CELL ADHESION MOLECULES CAMS	0.018627739	-1.327339402	4.50E-05	0.000126716	130

EPITHELIAL CELL SIGNALING IN HELICOBACTER PYLORI INFECTION	0.091243058	-1.276210364	7.11E-05	0.000197417	68
PPAR SIGNALING PATHWAY	0.079866845	-1.273196557	7.75E-05	0.000212103	69
AUTOIMMUNE THYROID DISEASE	0.041614643	-1.257196743	0.000127	0.000341566	49
ERBB SIGNALING PATHWAY	0.107298569	-1.175138575	0.000225	0.000597392	87
AMYOTROPHIC LATERAL SCLEROSIS ALS	0.119329512	-1.142009792	0.000334	0.000875483	53
PROTEASOME	0.072631327	-1.169020316	0.000367	0.000949219	44

FDR, false discovery rate.

Supplementary Table 8. EGG pathways significantly upregulated in hepatoblastoma cluster E1 compared to normal liver.

KEGG pathway	Geometric mean of the individual *P* values	Mean of the individual statistics	*P* value	FDR *q* value	Set size
RIBOSOME	1.89E-05	3.781188033	6.85E-44	1.27E-41	87
CELL CYCLE	0.000198776	3.383508387	3.04E-38	2.82E-36	125
ECM RECEPTOR INTERACTION	0.000152553	3.371348757	9.58E-37	5.94E-35	84
FOCAL ADHESION	0.001650452	2.367319325	6.35E-20	2.95E-18	199
BASAL CELL CARCINOMA	0.009399695	2.233203537	1.26E-17	4.70E-16	55
ARRHYTHMOGENIC RIGHT VENTRICULAR CARDIOMYOPATHY ARVC	0.008269371	2.147703143	1.60E-16	4.95E-15	74
PATHWAYS IN CANCER	0.009744893	1.996946003	6.69E-15	1.78E-13	325
DNA REPLICATION	0.0138975	2.039513587	1.35E-14	3.13E-13	36
WNT SIGNALING PATHWAY	0.020402981	1.881919121	2.18E-13	4.50E-12	151
AXON GUIDANCE	0.02206065	1.768805669	5.45E-12	1.01E-10	129
DILATED CARDIOMYOPATHY	0.017727513	1.695703587	5.07E-11	8.58E-10	90
HOMOLOGOUS RECOMBINATION	0.041696855	1.650974256	2.39E-10	3.71E-09	28
REGULATION OF ACTIN CYTOSKELETON	0.02673674	1.608619555	2.85E-10	4.08E-09	212
SMALL CELL LUNG CANCER	0.040823363	1.570207474	8.39E-10	1.12E-08	84
HYPERTROPHIC CARDIOMYOPATHY HCM	0.026712196	1.572390004	9.73E-10	1.21E-08	83
GAP JUNCTION	0.059109588	1.42503451	2.12E-08	2.47E-07	89
GLYCOSPHINGOLIPID BIOSYNTHESIS GLOBO SERIES	0.084902807	1.39653976	8.32E-08	9.10E-07	14
HEDGEHOG SIGNALING PATHWAY	0.060816922	1.305893482	3.22E-07	3.33E-06	56
PROGESTERONE MEDIATED OOCYTE MATURATION	0.092228415	1.256405726	6.48E-07	6.35E-06	85
GLYCOSAMINOGLYCAN BIOSYNTHESIS HEPARAN SULFATE	0.115736707	1.169026011	4.16E-06	3.87E-05	26
O GLYCAN BIOSYNTHESIS	0.122211456	1.061567223	2.62E-05	0.000231637	29
MISMATCH REPAIR	0.116034948	1.05683994	3.30E-05	0.00027917	23
MELANOMA	0.123863994	1.019763314	4.46E-05	0.000351972	71
NON HOMOLOGOUS END JOINING	0.136218687	1.05227535	4.54E-05	0.000351972	13
MELANOGENESIS	0.129797693	0.992754311	6.49E-05	0.000482746	101

FDR, false discovery rate.

Supplementary Table 9. KEGG pathways significantly downregulated in hepatoblastoma cluster E1 compared to normal liver.

KEGG pathway	Geometric mean of the individual *P* values	Mean of the individual statistics	*P* value	FDR *q* value	Set size
RETINOL METABOLISM	5.26E-13	-8.37827914	6.32E-167	1.18E-164	64
DRUG METABOLISM CYTOCHROME P450	1.49E-12	-8.009683186	2.39E-160	2.22E-158	72
METABOLISM OF XENOBIOTICS BY CYTOCHROME P450	1.07E-11	-7.5942795	1.19E-147	7.37E-146	70
DRUG METABOLISM OTHER ENZYMES	3.39E-10	-7.155974351	6.41E-126	2.98E-124	51
STEROID HORMONE BIOSYNTHESIS	1.29E-09	-6.715541181	7.24E-117	2.69E-115	55
COMPLEMENT AND COAGULATION CASCADES	4.71E-09	-6.153219636	3.80E-108	1.18E-106	69
ASCORBATE AND ALDARATE METABOLISM	5.55E-09	-7.24628397	8.66E-108	2.30E-106	25
PENTOSE AND GLUCURONATE INTERCONVERSIONS	1.33E-07	-6.161584492	3.35E-88	7.78E-87	28
STARCH AND SUCROSE METABOLISM	1.51E-07	-5.621776395	1.34E-87	2.78E-86	52
PORPHYRIN AND CHLOROPHYLL METABOLISM	1.48E-06	-5.143486667	1.13E-72	2.10E-71	41
FATTY ACID METABOLISM	2.31E-06	-4.933854964	1.43E-69	2.41E-68	42
VALINE LEUCINE AND ISOLEUCINE DEGRADATION	2.76E-06	-4.805583125	1.03E-67	1.60E-66	44
PEROXISOME	3.40E-06	-4.621301792	1.49E-66	2.13E-65	78
TRYPTOPHAN METABOLISM	1.70E-05	-4.47290958	6.12E-58	8.13E-57	40
GLYCINE SERINE AND THREONINE METABOLISM	5.18E-05	-4.162625037	6.17E-50	7.65E-49	31
PROPANOATE METABOLISM	0.000118005	-3.874291315	4.82E-45	5.60E-44	32
CYTOKINE CYTOKINE RECEPTOR INTERACTION	5.63E-05	-3.515905753	1.23E-41	1.34E-40	264
PRIMARY BILE ACID BIOSYNTHESIS	0.000244347	-3.974743731	2.04E-41	2.10E-40	16
GLYCOLYSIS GLUCONEOGENESIS	0.00039371	-3.428744928	2.83E-38	2.77E-37	62
TYROSINE METABOLISM	0.000727555	-3.309254961	7.79E-35	7.24E-34	41
JAK STAT SIGNALING PATHWAY	0.000651241	-3.181489822	1.67E-34	1.48E-33	154
HISTIDINE METABOLISM	0.00119794	-3.200976009	7.15E-32	6.05E-31	28
LINOLEIC ACID METABOLISM	0.001316626	-3.220183203	1.85E-31	1.50E-30	29
BUTANOATE METABOLISM	0.001339697	-3.089896429	1.43E-30	1.11E-29	34
PARKINSONS DISEASE	0.000536184	-3.008374397	1.99E-30	1.48E-29	112
PPAR SIGNALING PATHWAY	0.001289627	-3.007033779	3.10E-30	2.22E-29	69
OXIDATIVE PHOSPHORYLATION	0.000417652	-2.978756479	1.15E-29	7.96E-29	116
CITRATE CYCLE TCA CYCLE	0.002058145	-2.961618931	2.94E-28	1.95E-27	30
GRAFT VERSUS HOST DISEASE	0.000548534	-2.978433536	2.26E-27	1.45E-26	39
ARACHIDONIC ACID METABOLISM	0.002408298	-2.83606448	7.17E-27	4.44E-26	58
PYRUVATE METABOLISM	0.002825426	-2.8138838	2.15E-26	1.29E-25	40
PRION DISEASES	0.003074871	-2.810516375	7.90E-26	4.59E-25	35
BETA ALANINE METABOLISM	0.00326917	-2.880753132	1.33E-25	7.49E-25	22
ALLOGRAFT REJECTION	0.001644295	-2.539614251	2.46E-20	1.33E-19	35
HUNTINGTONS DISEASE	0.004511277	-2.386150215	2.56E-20	1.33E-19	172
APOPTOSIS	0.0074727	-2.396461403	2.57E-20	1.33E-19	87
NATURAL KILLER CELL MEDIATED CYTOTOXICITY	0.004142579	-2.384372419	3.37E-20	1.69E-19	136
LIMONENE AND PINENE DEGRADATION	0.010359567	-2.535416966	3.08E-19	1.51E-18	10
ARGININE AND PROLINE METABOLISM	0.009591255	-2.334773808	3.65E-19	1.74E-18	53
LYSINE DEGRADATION	0.011903085	-2.2774497	2.89E-18	1.34E-17	44
CYSTEINE AND METHIONINE METABOLISM	0.013754685	-2.230840871	2.25E-17	1.02E-16	34
ABC TRANSPORTERS	0.01434433	-2.204349521	3.04E-17	1.35E-16	44
PHENYLALANINE METABOLISM	0.015877802	-2.233469761	8.51E-17	3.68E-16	18
ALANINE ASPARTATE AND GLUTAMATE METABOLISM	0.014588186	-2.190227101	9.08E-17	3.84E-16	32
CHEMOKINE SIGNALING PATHWAY	0.005288412	-2.081811219	6.77E-16	2.80E-15	189
ALZHEIMERS DISEASE	0.013781962	-2.073910265	7.21E-16	2.92E-15	157
TOLL LIKE RECEPTOR SIGNALING PATHWAY	0.010521229	-2.064125225	1.42E-15	5.64E-15	101
AUTOIMMUNE THYROID DISEASE	0.007190336	-2.037309826	1.33E-14	5.14E-14	49
B CELL RECEPTOR SIGNALING PATHWAY	0.01502943	-1.968552119	2.96E-14	1.13E-13	75
NOD LIKE RECEPTOR SIGNALING PATHWAY	0.017904022	-1.960932833	3.83E-14	1.42E-13	62
INSULIN SIGNALING PATHWAY	0.028057977	-1.897001938	1.31E-13	4.76E-13	137
INTESTINAL IMMUNE NETWORK FOR IGA PRODUCTION	0.008511317	-1.907513647	4.93E-13	1.76E-12	46
GLUTATHIONE METABOLISM	0.027430973	-1.856083296	8.09E-13	2.84E-12	50
FOLATE BIOSYNTHESIS	0.033673622	-1.91874462	1.54E-12	5.29E-12	11
ANTIGEN PROCESSING AND PRESENTATION	0.014653623	-1.816661194	1.98E-12	6.71E-12	85
AMINO SUGAR AND NUCLEOTIDE SUGAR METABOLISM	0.034323071	-1.789880196	4.39E-12	1.46E-11	44
GLYOXYLATE AND DICARBOXYLATE METABOLISM	0.038505134	-1.835037936	5.83E-12	1.90E-11	16
PROTEASOME	0.022199721	-1.820264862	6.28E-12	2.01E-11	44
ADIPOCYTOKINE SIGNALING PATHWAY	0.03795058	-1.758275881	7.40E-12	2.33E-11	67
CYTOSOLIC DNA SENSING PATHWAY	0.029484408	-1.758332589	9.91E-12	3.07E-11	55
LEISHMANIA INFECTION	0.012806432	-1.756481122	1.41E-11	4.31E-11	70
CIRCADIAN RHYTHM MAMMAL	0.04151255	-1.78161708	2.57E-11	7.70E-11	13
MATURITY ONSET DIABETES OF THE YOUNG	0.043010746	-1.664538126	1.69E-10	4.99E-10	25
NICOTINATE AND NICOTINAMIDE METABOLISM	0.05308747	-1.637710644	2.92E-10	8.48E-10	24
BIOSYNTHESIS OF UNSATURATED FATTY ACIDS	0.050589118	-1.618512312	5.32E-10	1.52E-09	22
GLYCEROLIPID METABOLISM	0.055189557	-1.540524403	1.80E-09	5.09E-09	49
FRUCTOSE AND MANNOSE METABOLISM	0.074741468	-1.430209436	2.35E-08	6.53E-08	34

PANTOTHENATE AND COA BIOSYNTHESIS	0.078949008	-1.425370805	4.82E-08	1.32E-07	16
SELENOAMINO ACID METABOLISM	0.081785874	-1.384042162	6.97E-08	1.88E-07	25
GALACTOSE METABOLISM	0.082511887	-1.376185755	8.04E-08	2.14E-07	26
ASTHMA	0.02039899	-1.438325666	8.24E-08	2.16E-07	28
TYPE I DIABETES MELLITUS	0.034970544	-1.343446091	2.06E-07	5.32E-07	41
VIRAL MYOCARDITIS	0.032708216	-1.299000672	4.04E-07	1.03E-06	68
PENTOSE PHOSPHATE PATHWAY	0.093036115	-1.290252251	4.46E-07	1.12E-06	27
PROTEIN EXPORT	0.084751709	-1.247270703	2.31E-06	5.72E-06	23
RIG I LIKE RECEPTOR SIGNALING PATHWAY	0.109028079	-1.13813204	5.94E-06	1.45E-05	70
ONE CARBON POOL BY FOLATE	0.122871331	-1.137608329	8.41E-06	2.03E-05	17
PYRIMIDINE METABOLISM	0.099349174	-1.075149127	1.76E-05	4.20E-05	98
TERPENOID BACKBONE BIOSYNTHESIS	0.128983336	-0.960451088	0.000175	0.000411204	15
EPITHELIAL CELL SIGNALING IN HELICOBACTER PYLORI INFECTION	0.178569002	-0.875994722	0.000365	0.000849659	68
CELL ADHESION MOLECULES CAMS	0.038279389	-0.881727037	0.000375	0.000861743	130
PROXIMAL TUBULE BICARBONATE RECLAMATION	0.177617886	-0.882761407	0.000384	0.00087207	23

FDR, false discovery rate.

Supplementary Table 10. KEGG pathways significantly upregulated in hepatoblastoma cluster E2 compared to normal liver.

KEGG pathway	Geometric mean of the individual *P* values	Mean of the individual statistics	*P* value	FDR *q* value	Set size
RIBOSOME	1.67E-06	4.651474179	1.74E-47	3.24E-45	87
CELL CYCLE	0.000257603	3.253404831	1.38E-26	1.28E-24	125
BASAL CELL CARCINOMA	0.034535129	1.719254639	9.66E-09	5.99E-07	55
ECM RECEPTOR INTERACTION	0.014275875	1.683553978	2.35E-08	1.09E-06	84
DNA REPLICATION	0.024226215	1.658460919	5.49E-08	2.04E-06	36
WNT SIGNALING PATHWAY	0.043389985	1.59209139	7.43E-08	2.30E-06	151
HOMOLOGOUS RECOMBINATION	0.046127155	1.613287881	8.93E-08	2.37E-06	28
ARRHYTHMOGENIC RIGHT VENTRICULAR CARDIOMYOPATHY ARVC	0.059496504	1.393562868	2.35E-06	5.45E-05	74
PATHWAYS IN CANCER	0.040957314	1.328575756	5.67E-06	0.000117083	325

FDR, false discovery rate.

Supplementary Table 11. KEGG pathways significantly downregulated in hepatoblastoma cluster E2 compared to normal liver.

KEGG pathway	Geometric mean of the individual *P* values	Mean of the individual statistics	*P* value	FDR *q* value	Set size
RETINOL METABOLISM	1.05E-12	-8.20408057	9.76E-120	1.82E-117	64
DRUG METABOLISM CYTOCHROME P450	3.12E-12	-7.848969433	9.98E-115	9.28E-113	72
METABOLISM OF XENOBIOTICS BY CYTOCHROME P450	3.49E-11	-7.33237745	2.73E-103	1.69E-101	70
DRUG METABOLISM OTHER ENZYMES	2.66E-09	-6.684049189	1.54E-83	7.15E-82	51
COMPLEMENT AND COAGULATION CASCADES	2.22E-09	-6.264497658	4.37E-83	1.62E-81	69
STEROID HORMONE BIOSYNTHESIS	8.44E-09	-6.281585765	7.97E-78	2.47E-76	55
ASCORBATE AND ALDARATE METABOLISM	1.34E-08	-6.983214498	1.67E-75	4.43E-74	25
STARCH AND SUCROSE METABOLISM	2.45E-07	-5.499127132	1.43E-62	3.32E-61	52
PENTOSE AND GLUCURONATE INTERCONVERSIONS	2.34E-07	-5.963936228	1.66E-62	3.43E-61	28
VALINE LEUCINE AND ISOLEUCINE DEGRADATION	6.50E-07	-5.194660477	1.65E-57	3.07E-56	44
PORPHYRIN AND CHLOROPHYLL METABOLISM	8.73E-07	-5.288773637	1.20E-56	2.03E-55	41
FATTY ACID METABOLISM	3.33E-06	-4.827434346	4.81E-50	7.46E-49	42
PEROXISOME	7.77E-06	-4.445300562	3.08E-46	4.40E-45	78
GLYCINE SERINE AND THREONINE METABOLISM	9.15E-06	-4.656466458	3.63E-45	4.82E-44	31
TRYPTOPHAN METABOLISM	1.29E-05	-4.540885466	2.58E-44	3.20E-43	40
CYTOKINE CYTOKINE RECEPTOR INTERACTION	1.52E-06	-4.083608969	6.67E-41	7.76E-40	264
PROPANOATE METABOLISM	5.54E-05	-4.125550561	1.41E-37	1.54E-36	32
GLYCOLYSIS GLUCONEOGENESIS	0.00022768	-3.600016101	2.98E-31	3.08E-30	62
TYROSINE METABOLISM	0.000872951	-3.256061499	1.83E-25	1.77E-24	41
PRIMARY BILE ACID BIOSYNTHESIS	0.000758262	-3.507572824	1.90E-25	1.77E-24	16
JAK STAT SIGNALING PATHWAY	0.000404967	-3.091896288	2.08E-24	1.85E-23	154
LINOLEIC ACID METABOLISM	0.001621896	-3.135265978	1.46E-22	1.23E-21	29
BUTANOATE METABOLISM	0.001774809	-3.010990606	5.42E-22	4.38E-21	34
HISTIDINE METABOLISM	0.002077947	-2.994467823	1.97E-21	1.53E-20	28
PARKINSONS DISEASE	0.00119037	-2.859343846	5.77E-21	4.29E-20	112
PRION DISEASES	0.00213254	-2.909920224	1.37E-20	9.83E-20	35
CITRATE CYCLE TCA CYCLE	0.002600745	-2.874724322	3.00E-20	2.07E-19	30
PYRUVATE METABOLISM	0.00285705	-2.823086808	4.86E-20	3.23E-19	40
GRAFT VERSUS HOST DISEASE	0.000231953	-2.872526011	8.53E-20	5.47E-19	39
PPAR SIGNALING PATHWAY	0.003165164	-2.716352183	5.12E-19	3.17E-18	69
ARACHIDONIC ACID METABOLISM	0.003633664	-2.705209816	1.21E-18	7.23E-18	58
BETA ALANINE METABOLISM	0.004524271	-2.7558169	4.51E-18	2.62E-17	22
CHEMOKINE SIGNALING PATHWAY	0.000734096	-2.556159332	2.44E-17	1.37E-16	189
APOPTOSIS	0.00473195	-2.521297983	8.50E-17	4.65E-16	87
TOLL LIKE RECEPTOR SIGNALING PATHWAY	0.003292287	-2.426266281	1.08E-15	5.76E-15	101
ALLOGRAFT REJECTION	0.000734252	-2.463401519	1.76E-15	9.09E-15	35
OXIDATIVE PHOSPHORYLATION	0.003630667	-2.395873607	2.56E-15	1.29E-14	116
CYSTEINE AND METHIONINE METABOLISM	0.010098959	-2.375586816	9.55E-15	4.67E-14	34
ARGININE AND PROLINE METABOLISM	0.009863372	-2.339893647	1.25E-14	5.94E-14	53
PHENYLALANINE METABOLISM	0.012997539	-2.328412682	1.03E-13	4.77E-13	18
LIMONENE AND PINENE DEGRADATION	0.013645842	-2.397108522	2.03E-13	9.20E-13	10
LYSINE DEGRADATION	0.014988077	-2.165405627	9.68E-13	4.29E-12	44
ABC TRANSPORTERS	0.014388688	-2.149691339	1.57E-12	6.66E-12	44
LEISHMANIA INFECTION	0.002695218	-2.158786243	1.57E-12	6.66E-12	70
B CELL RECEPTOR SIGNALING PATHWAY	0.008873942	-2.103617307	3.34E-12	1.38E-11	75
HUNTINGTONS DISEASE	0.012516161	-2.069233494	4.58E-12	1.85E-11	172
ASTHMA	0.004966102	-2.100712199	1.99E-11	7.88E-11	28
NOD LIKE RECEPTOR SIGNALING PATHWAY	0.009671298	-2.024575477	2.13E-11	8.25E-11	62
INTESTINAL IMMUNE NETWORK FOR IGA PRODUCTION	0.005130027	-2.035575916	2.32E-11	8.82E-11	46
ALANINE ASPARTATE AND GLUTAMATE METABOLISM	0.023765579	-2.000733794	4.81E-11	1.79E-10	32
AUTOIMMUNE THYROID DISEASE	0.005611884	-1.967842947	9.06E-11	3.31E-10	49
NATURAL KILLER CELL MEDIATED CYTOTOXICITY	0.006747581	-1.92386051	1.15E-10	4.10E-10	136
INSULIN SIGNALING PATHWAY	0.025924904	-1.907229531	1.55E-10	5.42E-10	137
ALZHEIMERS DISEASE	0.021567352	-1.866412133	3.77E-10	1.30E-09	157
CELL ADHESION MOLECULES CAMS	0.007755397	-1.870108639	4.30E-10	1.46E-09	130
ADIPOCYTOKINE SIGNALING PATHWAY	0.031002955	-1.834947387	8.35E-10	2.77E-09	67
GLUTATHIONE METABOLISM	0.033933305	-1.829637207	1.06E-09	3.45E-09	50
ANTIGEN PROCESSING AND PRESENTATION	0.009722715	-1.799132987	1.64E-09	5.26E-09	85
CYTOSOLIC DNA SENSING PATHWAY	0.027189562	-1.750380553	5.19E-09	1.64E-08	55
GLYOXYLATE AND DICARBOXYLATE METABOLISM	0.042295942	-1.789878448	6.37E-09	1.98E-08	16
AMINO SUGAR AND NUCLEOTIDE SUGAR METABOLISM	0.038777093	-1.735959764	6.85E-09	2.09E-08	44
FOLATE BIOSYNTHESIS	0.04764942	-1.745781324	1.84E-08	5.52E-08	11
BIOSYNTHESIS OF UNSATURATED FATTY ACIDS	0.043337754	-1.689554265	2.78E-08	8.20E-08	22
NICOTINATE AND NICOTINAMIDE METABOLISM	0.050171784	-1.666979488	3.38E-08	9.81E-08	24
VIRAL MYOCARDITIS	0.015242452	-1.639853034	3.92E-08	1.12E-07	68
GLYCEROLIPID METABOLISM	0.049246098	-1.610985791	6.45E-08	1.82E-07	49
SELENOAMINO ACID METABOLISM	0.064515263	-1.501033223	5.36E-07	1.49E-06	25

PROTEASOME	0.058146649	-1.456490782	1.15E-06	3.15E-06	44
CIRCADIAN RHYTHM MAMMAL	0.066398027	-1.476956614	1.41E-06	3.81E-06	13
MATURITY ONSET DIABETES OF THE YOUNG	0.074367398	-1.429441481	1.65E-06	4.40E-06	25
FRUCTOSE AND MANNOSE METABOLISM	0.076970858	-1.416893059	1.78E-06	4.67E-06	34
PANTOTHENATE AND COA BIOSYNTHESIS	0.080931356	-1.429310289	2.29E-06	5.92E-06	16
PENTOSE PHOSPHATE PATHWAY	0.079978975	-1.393475083	2.74E-06	6.98E-06	27
GALACTOSE METABOLISM	0.08240239	-1.377569967	3.54E-06	8.89E-06	26
NEUROTROPHIN SIGNALING PATHWAY	0.106472422	-1.166335438	5.77E-05	0.000143161	126
PROXIMAL TUBULE BICARBONATE RECLAMATION	0.116727013	-1.180422001	6.06E-05	0.0001484	23
TYPE I DIABETES MELLITUS	0.033069782	-1.112430452	9.38E-05	0.000226515	41
ERBB SIGNALING PATHWAY	0.121679458	-1.089600534	0.000161	0.000383362	87
RIG I LIKE RECEPTOR SIGNALING PATHWAY	0.113657659	-1.082622266	0.000179	0.000418804	70
HEMATOPOIETIC CELL LINEAGE	0.048560724	-1.080004729	0.00018	0.000418804	85
ONE CARBON POOL BY FOLATE	0.138460097	-1.082298399	0.000217	0.000499305	17
EPITHELIAL CELL SIGNALING IN HELICOBACTER PYLORI INFECTION	0.122324091	-1.029404369	0.000344	0.000779676	68

FDR, false discovery rate.

Supplementary Table 12. KEGG pathway significantly upregulated in hepatoblastoma cluster E2 compared to cluster E1.

KEGG pathway	Geometric mean of the individual *P* values	Mean of the individual statistics	*P* value	FDR *q* value	Set size
COMPLEMENT AND COAGULATION CASCADES	0.019923945	1.491627736	6.06E-07	0.000112771	69

FDR, false discovery rate.

Supplementary Table 13. KEGG pathways significantly downregulated in hepatoblastoma cluster E2 compared to cluster E1.

KEGG pathway	Geometric mean of the individual *P* values	Mean of the individual statistics	*P* value	FDR *q* value	Set size
ECM RECEPTOR INTERACTION	0.000168484	-2.95341766	3.08E-21	5.73E-19	84
FOCAL ADHESION	0.000135623	-2.844130065	8.97E-21	8.35E-19	199
NEUROACTIVE LIGAND RECEPTOR INTERACTION	0.017207049	-1.663937738	2.04E-08	1.26E-06	271
TGF BETA SIGNALING PATHWAY	0.030533607	-1.575288972	1.21E-07	5.64E-06	86
CELL ADHESION MOLECULES CAMS	0.005417538	-1.478381442	7.00E-07	2.60E-05	130
AXON GUIDANCE	0.02936869	-1.328852498	6.76E-06	0.000209427	129
CALCIUM SIGNALING PATHWAY	0.040776669	-1.290646889	1.05E-05	0.00027953	177
DILATED CARDIOMYOPATHY	0.03599648	-1.219012985	3.34E-05	0.000776923	90

FDR, false discovery rate.

Supplementary Table 14. KEGG pathways significantly upregulated in hepatoblastoma cluster E1 compared to cluster F.

KEGG pathway	Geometric mean of the individual *P* values	Mean of the individual statistics	*P* value	FDR *q* value	Set size
CELL CYCLE	0.000216148	3.033546779	7.19E-31	1.34E-28	125
AXON GUIDANCE	0.000970638	2.854926084	6.64E-28	6.17E-26	129
ECM RECEPTOR INTERACTION	0.000422739	2.804728966	6.65E-26	4.12E-24	84
PATHWAYS IN CANCER	0.002510443	2.264065512	1.47E-18	6.86E-17	325
DNA REPLICATION	0.008082418	2.005823303	8.58E-14	3.19E-12	36
FOCAL ADHESION	0.001832311	1.916478676	1.23E-13	3.83E-12	199
REGULATION OF ACTIN CYTOSKELETON	0.01991516	1.544530112	1.39E-09	3.29E-08	212
DILATED CARDIOMYOPATHY	0.00985637	1.569145611	1.41E-09	3.29E-08	90
HOMOLOGOUS RECOMBINATION	0.041434858	1.504110747	8.62E-09	1.78E-07	28
SMALL CELL LUNG CANCER	0.042914877	1.392055339	4.88E-08	9.08E-07	84
MAPK SIGNALING PATHWAY	0.046034911	1.351034533	9.19E-08	1.55E-06	266
TGF BETA SIGNALING PATHWAY	0.043454534	1.351201571	1.15E-07	1.78E-06	86
HYPERTROPHIC CARDIOMYOPATHY HCM	0.021207992	1.33548443	1.96E-07	2.80E-06	83
OOCYTE MEIOSIS	0.070859432	1.272509082	4.85E-07	6.45E-06	113
BASAL CELL CARCINOMA	0.057426327	1.235811173	1.25E-06	1.55E-05	55
RIBOSOME	0.002390369	1.267804963	2.46E-06	2.86E-05	87
PROGESTERONE MEDIATED OOCYTE MATURATION	0.092675348	1.161833602	3.90E-06	4.27E-05	85
O GLYCAN BIOSYNTHESIS	0.0906829	1.175463383	4.28E-06	4.42E-05	29
PATHOGENIC ESCHERICHIA COLI INFECTION	0.100910505	1.155526698	4.64E-06	4.54E-05	55
HEMATOPOIETIC CELL LINEAGE	0.014786526	1.15718736	7.49E-06	6.97E-05	85
BLADDER CANCER	0.111742139	1.122218038	8.87E-06	7.86E-05	42
GAP JUNCTION	0.083539018	1.109850202	9.94E-06	8.41E-05	89
GLYCOSAMINOGLYCAN BIOSYNTHESIS CHONDROITIN SULFATE	0.109576559	1.122492464	1.07E-05	8.62E-05	22
MELANOGENESIS	0.093750328	1.074801553	1.77E-05	0.000137434	101
GLYCOSAMINOGLYCAN BIOSYNTHESIS HEPARAN SULFATE	0.129518355	1.071876192	2.22E-05	0.000164872	26
GLYCOSAMINOGLYCAN BIOSYNTHESIS KERATAN SULFATE	0.136111341	1.038025866	4.94E-05	0.000349235	15
HEDGEHOG SIGNALING PATHWAY	0.069589497	1.02516379	5.07E-05	0.000349235	56
NEUROACTIVE LIGAND RECEPTOR INTERACTION	0.049064449	0.987736252	7.06E-05	0.000468719	271
MISMATCH REPAIR	0.094727501	0.998459194	9.62E-05	0.00061691	23

FDR, false discovery rate.

Supplementary Table 15. KEGG pathways significantly downregulated in hepatoblastoma cluster E1 compared to cluster F.

KEGG pathway	Geometric mean of the individual *P* values	Mean of the individual statistics	*P* value	FDR *q* value	Set size
FATTY ACID METABOLISM	1.14E-05	-4.433243675	1.18E-57	2.20E-55	42
PEROXISOME	1.10E-05	-4.234292923	1.57E-56	1.46E-54	78
METABOLISM OF XENOBIOTICS BY CYTOCHROME P450	5.78E-06	-4.262573739	7.29E-54	4.52E-52	70
DRUG METABOLISM CYTOCHROME P450	2.92E-06	-4.260533552	4.57E-53	2.13E-51	72
RETINOL METABOLISM	2.39E-06	-4.2422904	2.13E-51	7.92E-50	64
COMPLEMENT AND COAGULATION CASCADES	0.000186826	-3.20843976	4.29E-33	1.33E-31	69
STEROID HORMONE BIOSYNTHESIS	0.000570715	-2.994624089	2.83E-28	7.51E-27	55
VALINE LEUCINE AND ISOLEUCINE DEGRADATION	0.001465515	-2.896157288	2.00E-27	4.64E-26	44
PPAR SIGNALING PATHWAY	0.001815788	-2.795468753	2.51E-26	5.18E-25	69
DRUG METABOLISM OTHER ENZYMES	0.000663308	-2.694913922	5.69E-23	1.06E-21	51
TRYPTOPHAN METABOLISM	0.004865648	-2.53175333	1.22E-21	2.05E-20	40
PROPANOATE METABOLISM	0.011788599	-2.195541259	1.11E-16	1.71E-15	32
TYROSINE METABOLISM	0.014097537	-2.167544945	1.34E-16	1.92E-15	41
ASCORBATE AND ALDARATE METABOLISM	0.00385388	-2.233221865	3.44E-15	4.57E-14	25
BUTANOATE METABOLISM	0.016919083	-2.05957478	4.70E-15	5.83E-14	34
PRIMARY BILE ACID BIOSYNTHESIS	0.021374042	-2.059103375	4.34E-14	5.05E-13	16
BETA ALANINE METABOLISM	0.020646132	-1.969757771	1.58E-13	1.73E-12	22
LIMONENE AND PINENE DEGRADATION	0.028569437	-2.026360182	2.65E-13	2.74E-12	10
OXIDATIVE PHOSPHORYLATION	0.003039843	-1.890120795	4.99E-13	4.88E-12	116
PENTOSE AND GLUCURONATE INTERCONVERSIONS	0.009072693	-1.915350497	3.51E-12	3.26E-11	28
CITRATE CYCLE TCA CYCLE	0.036970328	-1.738033042	3.48E-11	3.08E-10	30
LINOLEIC ACID METABOLISM	0.037390561	-1.719076101	6.09E-11	5.15E-10	29
LYSINE DEGRADATION	0.040162692	-1.672283928	9.52E-11	7.70E-10	44
SYSTEMIC LUPUS ERYTHEMATOSUS	0.014122669	-1.606804351	3.80E-10	2.95E-09	134
ADIPOCYTOKINE SIGNALING PATHWAY	0.052822079	-1.532538466	2.03E-09	1.51E-08	67
STARCH AND SUCROSE METABOLISM	0.022995986	-1.534248885	4.20E-09	3.00E-08	52
OLFACTORY TRANSDUCTION	0.036465751	-1.437380679	1.52E-08	1.05E-07	379
ARGININE AND PROLINE METABOLISM	0.057013421	-1.445203086	1.65E-08	1.10E-07	53
ALANINE ASPARTATE AND GLUTAMATE METABOLISM	0.059970167	-1.392266319	7.10E-08	4.55E-07	32
ALLOGRAFT REJECTION	0.006631078	-1.405437089	1.66E-07	1.03E-06	35
PYRUVATE METABOLISM	0.079842919	-1.318605857	2.34E-07	1.40E-06	40
ABC TRANSPORTERS	0.075051418	-1.300241772	3.53E-07	2.02E-06	44
PORPHYRIN AND CHLOROPHYLL METABOLISM	0.04062172	-1.321225321	3.59E-07	2.02E-06	41
PARKINSONS DISEASE	0.023770012	-1.26274158	7.22E-07	3.95E-06	112
GLYCINE SERINE AND THREONINE METABOLISM	0.06953102	-1.276774957	7.48E-07	3.98E-06	31
NATURAL KILLER CELL MEDIATED CYTOTOXICITY	0.022602063	-1.253047847	7.90E-07	4.08E-06	136
GLYCOLYSIS GLUCONEOGENESIS	0.09685533	-1.228806981	1.18E-06	5.94E-06	62
GRAFT VERSUS HOST DISEASE	0.008671614	-1.281504774	1.43E-06	6.99E-06	39
REGULATION OF AUTOPHAGY	0.10353893	-1.208381322	2.22E-06	1.06E-05	34
BIOSYNTHESIS OF UNSATURATED FATTY ACIDS	0.100086738	-1.186627506	3.50E-06	1.63E-05	22
AUTOIMMUNE THYROID DISEASE	0.017216745	-1.18766927	4.44E-06	2.01E-05	49
ANTIGEN PROCESSING AND PRESENTATION	0.030276364	-1.156392637	4.77E-06	2.11E-05	85
CIRCADIAN RHYTHM MAMMAL	0.110671246	-1.146068533	1.07E-05	4.64E-05	13
ALDOSTERONE REGULATED SODIUM REABSORPTION	0.112347349	-1.089573741	1.52E-05	6.44E-05	42
PROTEASOME	0.057374381	-1.112954717	1.80E-05	7.43E-05	44
HISTIDINE METABOLISM	0.125155535	-1.000631628	7.32E-05	0.000295897	28
ARACHIDONIC ACID METABOLISM	0.093459133	-0.972089742	9.86E-05	0.000390101	58
INSULIN SIGNALING PATHWAY	0.15429201	-0.961303726	0.000102	0.000395845	137
RIG I LIKE RECEPTOR SIGNALING PATHWAY	0.124878271	-0.957584557	0.000115	0.000437698	70
GLYOXYLATE AND DICARBOXYLATE METABOLISM	0.167792998	-0.923968887	0.00023	0.000854207	16

FDR, false discovery rate.

Supplementary Table 16. KEGG pathways significantly upregulated in hepatoblastoma cluster E2 compared to cluster F.

KEGG pathway	Geometric mean of the individual *P* values	Mean of the individual statistics	*P* value	FDR *q* value	Set size
CELL CYCLE	0.000151195	3.016545894	6.49E-23	1.21E-20	125
RIBOSOME	0.000465169	2.394938701	1.64E-13	1.53E-11	87
AXON GUIDANCE	0.009952594	1.973393952	4.80E-11	2.98E-09	129
DNA REPLICATION	0.016151838	1.521416751	7.48E-07	3.48E-05	36
HOMOLOGOUS RECOMBINATION	0.049433602	1.458256628	1.30E-06	4.83E-05	28
SPLICEOSOME	0.046323116	1.350284801	4.67E-06	0.000128342	126
PATHWAYS IN CANCER	0.021540111	1.341801546	4.83E-06	0.000128342	325

FDR, false discovery rate.

Supplementary Table 17. KEGG pathways significantly downregulated in hepatoblastoma cluster E2 compared to cluster F.

KEGG pathway	Geometric mean of the individual *P* values	Mean of the individual statistics	*P* value	FDR *q* value	Set size
FATTY ACID METABOLISM	2.15E-05	-4.270257302	1.49E-40	2.76E-38	42
PEROXISOME	4.40E-05	-3.944893349	3.00E-37	2.79E-35	78
DRUG METABOLISM CYTOCHROME P450	1.04E-05	-3.88693058	1.24E-33	7.68E-32	72
RETINOL METABOLISM	1.18E-05	-3.83503702	2.20E-32	1.02E-30	64
METABOLISM OF XENOBIOTICS BY CYTOCHROME P450	3.05E-05	-3.684319118	1.03E-30	3.83E-29	70
VALINE LEUCINE AND ISOLEUCINE DEGRADATION	0.00044922	-3.370595174	5.42E-27	1.68E-25	44
COMPLEMENT AND COAGULATION CASCADES	0.001325622	-2.65313446	8.80E-18	2.34E-16	69
PPAR SIGNALING PATHWAY	0.004767711	-2.457695189	7.27E-16	1.69E-14	69
PROPANOATE METABOLISM	0.006890051	-2.483648617	9.37E-16	1.94E-14	32
TRYPTOPHAN METABOLISM	0.006506168	-2.388448892	8.79E-15	1.63E-13	40
STEROID HORMONE BIOSYNTHESIS	0.002706542	-2.349968909	6.43E-14	1.09E-12	55
DRUG METABOLISM OTHER ENZYMES	0.001384768	-2.301094969	3.25E-13	5.03E-12	51
BUTANOATE METABOLISM	0.01335432	-2.197918108	7.32E-13	1.05E-11	34
TYROSINE METABOLISM	0.018046739	-2.047712162	1.57E-11	2.08E-10	41
ADIPOCYTOKINE SIGNALING PATHWAY	0.031204593	-1.756320218	4.20E-09	5.21E-08	67
ASCORBATE AND ALDARATE METABOLISM	0.005966455	-1.902388251	6.79E-09	7.89E-08	25
LIMONENE AND PINENE DEGRADATION	0.040224973	-1.818922873	1.08E-08	1.18E-07	10
CITRATE CYCLE TCA CYCLE	0.035717716	-1.710024277	2.07E-08	2.13E-07	30
ASTHMA	0.008219124	-1.704389942	2.86E-08	2.80E-07	28
SYSTEMIC LUPUS ERYTHEMATOSUS	0.007782502	-1.62736535	5.55E-08	4.96E-07	134
GLYCINE SERINE AND THREONINE METABOLISM	0.042570117	-1.637092669	5.60E-08	4.96E-07	31
ALLOGRAFT REJECTION	0.003634473	-1.492918962	1.32E-07	1.10E-06	35
GRAFT VERSUS HOST DISEASE	0.004342048	-1.551647637	1.36E-07	1.10E-06	39
PENTOSE AND GLUCURONATE INTERCONVERSIONS	0.009277219	-1.678610148	1.72E-07	1.33E-06	28
LINOLEIC ACID METABOLISM	0.0411059	-1.590347185	1.90E-07	1.41E-06	29
BETA ALANINE METABOLISM	0.058303928	-1.549076578	2.94E-07	2.10E-06	22
PORPHYRIN AND CHLOROPHYLL METABOLISM	0.012159933	-1.564554287	4.16E-07	2.87E-06	41
GLYCOLYSIS GLUCONEOGENESIS	0.058720831	-1.433551105	1.27E-06	8.46E-06	62
STARCH AND SUCROSE METABOLISM	0.019782744	-1.446371637	1.86E-06	1.19E-05	52
PYRUVATE METABOLISM	0.071223482	-1.382564623	3.05E-06	1.89E-05	40
ABC TRANSPORTERS	0.050562718	-1.382556251	3.56E-06	2.10E-05	44
LYSINE DEGRADATION	0.069317531	-1.373033184	3.61E-06	2.10E-05	44
ARGININE AND PROLINE METABOLISM	0.075585956	-1.342383487	5.26E-06	2.96E-05	53
BIOSYNTHESIS OF UNSATURATED FATTY ACIDS	0.07547535	-1.342444675	7.39E-06	4.04E-05	22
ANTIGEN PROCESSING AND PRESENTATION	0.022716694	-1.295681011	8.01E-06	4.26E-05	85
ALDOSTERONE REGULATED SODIUM REABSORPTION	0.083575127	-1.312497169	8.57E-06	4.43E-05	42
AUTOIMMUNE THYROID DISEASE	0.015983199	-1.289906139	8.94E-06	4.49E-05	49
PRIMARY BILE ACID BIOSYNTHESIS	0.066960747	-1.343243605	1.49E-05	7.28E-05	16
ALANINE ASPARTATE AND GLUTAMATE METABOLISM	0.104645185	-1.184751606	5.47E-05	0.000260779	32
OXIDATIVE PHOSPHORYLATION	0.028347353	-1.121116442	0.000118	0.000547558	116
PARKINSONS DISEASE	0.0415962	-1.104982373	0.000143	0.000646747	112
PROXIMAL TUBULE BICARBONATE RECLAMATION	0.124058004	-1.088924822	0.0002	0.000887842	23

FDR, false discovery rate.

Supplementary Table 18. The top 50 genomic regions significantly overlapped with methylation array probes differentially hypermethylated in hepatoblastoma clusters E1/E2 compared to cluster F.

Region name	Cell type	Tissue	Antibody	Treatment	Number of overlapping regions	Number of regions present in the query and absent in the reference	Number of regions absent in the query and present in the reference	Number of regions absent both in the query and the reference	False discovery rate *q* value	Size
cistrome_cistrome normal liver cells HNF4A	normal liver cells	liver	HNF4A	No treatment	759	89583	1241	774276	1.64E-227	72335
cistrome_cistrome normal liver cells CEBPa	normal liver cells	liver	CEBPa	No treatment	348	36898	1652	826961	4.27E-105	64362
Transcribed Segments	Hepg2	NA	NA	NA	718	144138	1282	719721	6.05E-92	222291
Transcribed Segments	K562	NA	NA	NA	637	126588	1363	737271	1.03E-80	336262
cistrome_epigenome LnCaP H3K4me1	LnCaP	prostate	H3K4me1	regular medium	454	82025	1546	781834	3.49E-65	69585
Transcribed Segments	H1hesc	NA	NA	NA	484	92403	1516	771456	5.49E-63	324955
Transcribed Segments	GM12878	NA	NA	NA	670	156912	1330	706947	1.19E-57	621374
Transcribed Segments	Huvec	NA	NA	NA	675	160496	1325	703363	8.34E-56	1498149
cistrome_epigenome VCaP H3K4me1	VCaP	prostate	H3K4me1	regular medium	381	73894	1619	789965	3.02E-46	63143
Transcribed Segments	HeLa-S3	NA	NA	NA	655	173081	1345	690778	3.47E-38	1472755
cistrome_cistrome LnCaP FoxA1	LnCaP	prostate	FoxA1	regular medium	133	15605	1867	848254	9.80E-34	51551
cistrome_cistrome LnCaP AR	LnCaP	prostate	AR	R1881(16hr)	90	9057	1910	854802	5.88E-27	41243
Liver;Prostate	NA	NA	NA	NA	16	108	1984	863751	6.17E-21	359
cistrome_epigenome LnCaP H3K36me3	LnCaP	prostate	H3K36me3	regular medium	218	45650	1782	818209	7.42E-21	46112
cistrome_epigenome VCaP H3K36me3	VCaP	prostate	H3K36me3	regular medium	128	26809	1872	837050	1.19E-11	50257
Weak Enhancer Segments	HeLa-S3	NA	NA	NA	90	16375	1910	847484	4.96E-11	62184
cistrome_cistrome abl AR	abl	prostate	AR	DHT(4hr)	41	4574	1959	859285	1.65E-10	5312
ChIP T-47D GATA3_(SC-268)	T-47D	NA	GATA3_(SC-268)	DMSO_0.02pct	96	18974	1904	844885	6.95E-10	37199
cistrome_cistrome T47D PGR	T47D	breast	PGR	E2(45min)	58	8993	1942	854866	2.14E-09	5404
cistrome_epigenome LnCaP H3K4me2	LnCaP	prostate	H3K4me2	vehicle	694	241488	1306	622371	4.21E-09	109826
cistrome_epigenome LnCaP H3K4me2	LnCaP	prostate	H3K4me2	DHT(16hr)	641	220108	1359	643751	4.21E-09	102032
cistrome_cistrome A594 cells NR3C1	A594 cells	lung	NR3C1	DEX(1hr)	90	18409	1910	845450	1.48E-08	11083
Epithelial;Brain;Skin;Fibroblast;Colon;Cervix;Liver;Endothelial;Muscle;Prostate	NA	NA	NA	NA	6	26	1994	863833	1.70E-08	78
ChIP ECC-1 GR	ECC-1	NA	GR	DEX_100nM	39	5040	1961	858819	2.74E-08	9255
Prostate	NA	NA	NA	NA	7	61	1993	863798	3.59E-08	629
ChIP T-47D ERalpha_a	T-47D	NA	ERalpha_a	Estradiol_10nM	32	3714	1968	860145	8.25E-08	10505
ChIP T-47D ERalpha_a	T-47D	NA	ERalpha_a	Genistein_100nM	32	3729	1968	860130	8.74E-08	10547
Enhancers Segments	HeLa-S3	NA	NA	NA	128	31143	1872	832716	9.26E-08	41844
cistrome_epigenome LnCaP H3K4me2	LnCaP	prostate	H3K4me2	DHT(4hr)	679	240105	1321	623754	1.07E-07	114149
ChIP MCF10A-Er-Src c-Fos	MCF10A-Er-Src	NA	c-Fos	4OHTAM_1uM_4hr	113	26455	1887	837404	1.11E-07	86323
Prostate	NA	NA	NA	NA	10	286	1990	863573	2.76E-07	1135
Liver;Prostate	NA	NA	NA	NA	14	716	1986	863143	2.96E-07	10182
Prostate Cancer and Muscle	NA	NA	NA	NA	15	852	1985	863007	3.15E-07	15238
Repressed Segments	H1hesc	NA	NA	NA	878	324781	1122	539078	4.27E-07	543759
ChIP A549 FOXA1_(SC-101058)	A549	NA	FOXA1_(SC-101058)	DEX_100nM	23	2206	1977	861653	4.96E-07	7695
Fibroblast;Muscle;Brain;Epithelial;Liver;Prostate;Skin;Bone	NA	NA	NA	NA	5	32	1995	863827	2.24E-06	84
cistrome_cistrome MCF-7 ESR1	MCF-7	breast	ESR1	E2(45min)	150	40654	1850	823205	2.53E-06	75236

ChIP MCF10A-Er-Src c-Fos	MCF10A-Er-Src	NA	c-Fos	EtOH_0.01pct	97	23467	1903	840392	5.99E-06	67918
ChIP ECC-1 ERalpha_a	ECC-1	NA	ERalpha_a	Estradiol_10nM	48	8815	1952	855044	9.30E-06	16091
cistrome_cistrome VCaP FoxA1	VCaP	prostate	FoxA1	regular medium	22	2558	1978	861301	2.36E-05	17139
ChIP HeLa-S3 p300_(SC-584)	HeLa-S3	NA	p300_(SC-584)	None	60	12664	1940	851195	2.72E-05	25854
cistrome_cistrome LnCaP AR	LnCaP	prostate	AR	DHT(4hr)	44	8092	1956	855767	2.95E-05	7059
Liver;Epithelial	NA	NA	NA	NA	19	1992	1981	861867	3.06E-05	31688
ChIP T-47D ERalpha_a	T-47D	NA	ERalpha_a	BPA_100nM	15	1265	1985	862594	3.52E-05	3902
cistrome_epigenome T47D H3K4me1	T47D	breast	H3K4me1	DMSO(45min-3hr)	178	52816	1822	811043	4.06E-05	44002
ChIP A549 GR	A549	NA	GR	DEX_5nM	30	4748	1970	859111	0.000104028	10279
ChIP MCF-7 GATA3_(SC-269)	MCF-7	NA	GATA3_(SC-269)	None	36	6329	1964	857530	0.000106679	12077
cistrome_cistrome metastatic prostate cancer tissue AR	metastatic prostate cancer tissue	prostate	AR	No treatment	12	891	1988	862968	0.000121887	7723
cistrome_epigenome VCaP Pan-H3	VCaP	prostate	Pan-H3	regular medium	37	6759	1963	857100	0.000174362	8924
Complex tissue specific	NA	NA	NA	NA	5	91	1995	863768	0.000202337	270

Supplementary Table 19. The top 50 genomic regions significantly overlapped with methylation array probes differentially hypomethylated in hepatoblastoma clusters E1/E2 compared to cluster F.

Region name	Cell type	Tissue	Antibody	Treatment	Number of overlapping regions	Number of regions present in the query and absent in the reference	Number of regions absent in the query and present in the reference	Number of regions absent both in the query and the reference	False discovery rate *q* value	Size
cistrome_epigenome NB4 cells H3K9K14ac	NB4 cells	hematopoietic stem cell	H3K9K14ac	ATRA(24hr)	1141	214013	859	649846	1.47E-202	46628
cistrome_epigenome Caco-2(proliferating) H3K4me2	Caco-2(proliferating)	intestine	H3K4me2	No treatment	1062	189248	938	674611	3.07E-199	105110
cistrome_epigenome MR4 cells H3K9K14ac	MR4 cells	hematopoietic stem cell	H3K9K14ac	ATRA(24hr)	1171	231719	829	632140	3.64E-191	65073
cistrome_epigenome MR4 cells H3K9me3	MR4 cells	hematopoietic stem cell	H3K9me3	No treatment	1161	229741	839	634118	1.15E-188	65649
cistrome_epigenome Caco-2(differentiated) H3K4me2	Caco-2(differentiated)	intestine	H3K4me2	No treatment	910	148680	1090	715179	2.72E-185	125160
cistrome_epigenome UPR9 cells H3K9K14ac	UPR9 cells	hematopoietic stem cell	H3K9K14ac	No treatment	1107	213432	893	650427	2.91E-184	49690
cistrome_epigenome UPR9 cells H3K9K14ac	UPR9 cells	hematopoietic stem cell	H3K9K14ac	Zinc(5hr)	1062	205944	938	657915	4.45E-171	52814
cistrome_epigenome MCF-7 H3K4me1	MCF-7	breast	H3K4me1	DMSO(3hr)	794	146173	1206	717686	1.38E-125	127623
TSS Segments	GM12878	NA	NA	NA	771	147683	1229	716176	7.18E-112	34222
Juvenile acute myeloid leukaemia cell line carrying t(8;21)	Leukaemia cell	NA	TCF3	NA	398	47898	1602	815961	1.42E-105	43270
cistrome_epigenome NB4 cells H3K9K14ac	NB4 cells	hematopoietic stem cell	H3K9K14ac	No treatment	723	141562	1277	722297	2.01E-98	14486
cistrome_epigenome T47D H3K4me1	T47D	breast	H3K4me1	DMSO(45min-3hr)	396	52598	1604	811261	2.23E-92	44002
cistrome_epigenome MCF-7 H3K9ac	MCF-7	breast	H3K9ac	E2(3hr)	918	212492	1082	651367	2.43E-92	42367
cistrome_epigenome MCF-7 H3K9ac	MCF-7	breast	H3K9ac	DMSO(3hr)	848	197194	1152	666665	2.20E-81	37717
TSS Segments	K562	NA	NA	NA	664	136023	1336	727836	3.87E-80	21127
cistrome_epigenome LnCaP H3K4me2	LnCaP	prostate	H3K4me2	DHT(16hr)	892	219857	1108	644002	3.14E-74	102032
cistrome_epigenome MCF-7 H3K4me3	MCF-7	breast	H3K4me3	E2(3hr)	909	229273	1091	634586	3.83E-71	46616
cistrome_epigenome VCaP H3K4me2	VCaP	prostate	H3K4me2	regular medium	924	237565	1076	626294	1.33E-68	50201
Adult chronic myeloid leukaemia (at blast crisis) cell line carrying t(9;22)	Leukaemia cell	NA	FOSB	NA	198	18810	1802	845049	1.55E-65	23883
cistrome_epigenome metastatic prostate cancer tissue H3K4me3	metastatic prostate cancer tissue	prostate	H3K4me3	No treatment	868	220700	1132	643159	7.59E-65	29141
cistrome_epigenome LnCaP H3K4me1	LnCaP	prostate	H3K4me1	regular medium	450	82029	1550	781830	5.04E-64	69585
cistrome_epigenome LnCaP H3K4me2	LnCaP	prostate	H3K4me2	DHT(4hr)	914	239870	1086	623989	4.30E-63	114149
cistrome_epigenome LnCaP H3K4me2	LnCaP	prostate	H3K4me2	vehicle	915	241267	1085	622592	3.34E-62	109826
cistrome_cistrome UPR9 cells RARA	UPR9 cells	hematopoietic stem cell	RARA	Zinc(5hr)	357	57758	1643	806101	3.26E-61	18248
cistrome_cistrome A594 cells PolII	A594 cells	lung	PolII	EtOH(1hr)	929	249122	1071	614737	4.95E-60	30955
cistrome_cistrome A594 cells PolII	A594 cells	lung	PolII	DEX(1hr)	842	218391	1158	645468	3.16E-58	26411
TSS Segments	H1hesc	NA	NA	NA	646	149105	1354	714754	7.81E-58	28620
Enhancers Segments	Huvec	NA	NA	NA	401	72446	1599	791413	3.36E-57	53526
cistrome_epigenome VCaP H3K4me1	VCaP	prostate	H3K4me1	regular medium	405	73870	1595	789989	8.40E-57	63143
cistrome_epigenome LnCaP H3K4me2	LnCaP	prostate	H3K4me2	regular medium	843	221448	1157	642411	6.43E-56	62551
cistrome_epigenome LnCaP H3K4me3	LnCaP	prostate	H3K4me3	DHT(4hr)	630	145799	1370	718060	1.16E-55	22170
cistrome_epigenome LnCaP H3K4me3	LnCaP	prostate	H3K4me3	regular medium	764	193992	1236	669867	1.58E-54	24259
cistrome_epigenome LnCaP H3K4me3	LnCaP	prostate	H3K4me3	Vehicle	677	165005	1323	698854	1.14E-52	22631

cistrome_cistrome UPR9 cells PML	UPR9 cells	hematopoietic stem cell	PML	Zinc(5hr)	221	28193	1779	835666	3.24E-52	16901
cistrome_epigenome VCaP H3K4me3	VCaP	prostate	H3K4me3	regular medium	818	216656	1182	647203	4.29E-52	31540
GM12878 cell from ENCODE project	B-cells	NA	RUNX3	NA	478	101260	1522	762599	7.11E-50	104018
cistrome_epigenome MCF-7 H3K4me3	MCF-7	breast	H3K4me3	DMSO(3hr)	818	220163	1182	643696	3.34E-49	41328
ChIP K562 COREST_(sc-30189)	K562	NA	COREST_(sc-30189)	None	247	35816	1753	828043	4.94E-49	35741
cistrome_epigenome T47D H3K4me1	T47D	breast	H3K4me1	E2(45min-3hr)	256	38481	1744	825378	3.29E-48	35778
K562 erythrocytic leukaemia cells (ATCC CCL-243)	Embryonic Stem Cell	NA	SAP30	NA	261	40085	1739	823774	1.41E-47	16520
ChIP K562 eGFP-JunD	K562	NA	eGFP-JunD	None	233	33632	1767	830227	1.99E-46	26674
cistrome_epigenome VCaP H3K4me3	VCaP	prostate	H3K4me3	EtOH(16hr)	756	200690	1244	663169	2.47E-46	22317
cistrome_epigenome VCaP H3K9me3	VCaP	prostate	H3K9me3	R1881(16hr)	756	200690	1244	663169	2.47E-46	22317
ChIP K562 eGFP-FOS	K562	NA	eGFP-FOS	None	100	6513	1900	857346	4.73E-46	10256
ChIP HUVEC GATA-2	HUVEC	NA	GATA-2	None	153	15858	1847	848001	6.16E-46	27454
cistrome_cistrome NB4 cells PolII	NB4 cells	hematopoietic stem cell	PolII	ATRA(24hr)	546	127339	1454	736520	1.01E-45	26728
cistrome_cistrome lymphoblastoid(GM10861) VDR	lymphoblastoid(GM 10861)	hematopoietic stem cell	VDR	calcitriol(36hr)	511	117315	1489	746544	4.52E-44	48385
Enhancers Segments	K562	NA	NA	NA	217	30946	1783	832913	6.56E-44	44380
cistrome_cistrome A594 cells PolIIser2	A594 cells	lung	PolIIser2	EtOH(1hr)	819	228501	1181	635358	4.31E-43	36552
cistrome_epigenome VCaP H3K4me3	VCaP	prostate	H3K4me3	R1881(16hr)	634	161339	1366	702520	2.17E-42	20760

Supplementary Table 20. List of 366 cancer-associated genes for which all coding exons were sequenced in targeted capture sequencing.

ABL1	*ABL2*	*AFF3*	*AKT1*	*AKT2*	*AKT3*	*ALK*
AMER1	*APC*	*AR*	*ARHGAP35*	*ARID1A*	*ARID1B*	*ARID2*
ASXL1	*ATM*	*ATR*	*ATRX*	*AURKA*	*AURKB*	*AXIN1*
AXIN2	*AXL*	*BAK1*	*BAP1*	*BARD1*	*BCL10*	*BCL2*
BCL2L1	*BCL2L2*	*BCL6*	*BCOR*	*BCORL1*	*BIRC3*	*BLM*
BRAF	*BRCA1*	*BRCA2*	*BRIP1*	*BTK*	*BUB1B*	*CAPRIN2*
CARD11	*CASP8*	*CBFB*	*CBL*	*CBLB*	*CCND1*	*CCND2*
CCND3	*CCNE1*	*CD274*	*CD79A*	*CD79B*	*CDC27*	*CDC73*
CDH1	*CDK12*	*CDK4*	*CDK6*	*CDK8*	*CDKN1A*	*CDKN1B*
CDKN2A	*CDKN2B*	*CDKN2C*	*CEBPA*	*CHEK1*	*CHEK2*	*CIC*
COL2A1	*COL5A1*	*COL5A2*	*CREBBP*	*CRKL*	*CRLF2*	*CSF1R*
CSMD1	*CSMD2*	*CSMD3*	*CTCF*	*CTNNA1*	*CTNNB1*	*CUL3*
CYLD	*DAXX*	*DDB2*	*DDR2*	*DICER1*	*DKK1*	*DKK2*
DKK3	*DKK4*	*DNMT3A*	*DPPA3*	*DROSHA*	*DST*	*ECT2L*
EGFR	*EP300*	*EPCAM*	*EPHA3*	*EPHA7*	*EPHB1*	*ERBB2*
ERBB3	*ERBB4*	*ERCC5*	*ERG*	*ESR1*	*ETV1*	*ETV4*
EWSR1	*EZH2*	*FAM46C*	*FANCA*	*FANCC*	*FANCD2*	*FANCE*
FANCF	*FANCG*	*FAS*	*FAT1*	*FBXO11*	*FBXW7*	*FEV*
FGF19	*FGF3*	*FGF4*	*FGFR1*	*FGFR2*	*FGFR3*	*FGFR4*
FH	*FLCN*	*FLI1*	*FLT1*	*FLT3*	*FLT4*	*FOXL2*
FOXO1	*FOXP1*	*FUBP1*	*FUS*	*FZD10*	*FZD3*	*GAB1*
GATA1	*GATA2*	*GATA3*	*GLI1*	*GNA11*	*GNAQ*	*GNAS*
GPC3	*GRB2*	*GRIN2A*	*H3F3A*	*H3F3B*	*HIST1H3B*	*HNF1A*
HRAS	*HSPH1*	*IDH1*	*IDH2*	*IGF1R*	*IGF2*	*IGF2R*
IKBKE	*IKZF1*	*IL6ST*	*IL7R*	*INO80D*	*IRF4*	*IRS2*
JAK1	*JAK2*	*JAK3*	*JMJD1C*	*JUN*	*KDM5C*	*KDM6A*
KDR	*KEAP1*	*KIT*	*KLF4*	*KLF6*	*KMT2A*	*KMT2B*
KMT2C	*KMT2D*	*KRAS*	*LGR5*	*LIN28A*	*LIN28B*	*LMO1*
LRP1	*LRP1B*	*LRP5*	*MAP2K1*	*MAP2K2*	*MAP2K4*	*MAP3K1*
MAP4K3	*MAPK1*	*MAPK3*	*MCL1*	*MDM2*	*MDM4*	*MED12*
MEN1	*MET*	*MITF*	*MLH1*	*MRE11A*	*MSH2*	*MSH6*
MTMR4	*MTOR*	*MUC16*	*MUC4*	*MUTYH*	*MYC*	*MYCL1*
MYCN	*MYD88*	*MYH2*	*MYOD1*	*NANOG*	*NBN*	*NCOA1*
NCOA2	*NES*	*NF1*	*NF2*	*NFE2L2*	*NFKBIA*	*NKX2-1*
NOTCH1	*NOTCH2*	*NOTCH3*	*NOTCH4*	*NPM1*	*NRAS*	*NSD1*
NTRK1	*NTRK3*	*OBSCN*	*PALB2*	*PARK2*	*PARP1*	*PAX3*
PAX5	*PAX7*	*PBRM1*	*PDCD1LG2*	*PDE11A*	*PDGFRA*	*PDGFRB*
PEG10	*PHF6*	*PHOX2B*	*PIK3C2B*	*PIK3CA*	*PIK3CB*	*PIK3CD*
PIK3CG	*PIK3R1*	*PIK3R2*	*PINK1*	*PLAG1*	*PLVAP*	*PMS2*
POLE	*POU5F1*	*PPP2R1A*	*PRB2*	*PRDM1*	*PRDM9*	*PRKAR1A*
PRKCZ	*PRKDC*	*PRKRIR*	*PTCH1*	*PTEN*	*PTPN11*	*PTPRB*
PTPRD	*PTPRN*	*PTPRO*	*PTPRT*	*PZP*	*RAC1*	*RAD17*
RAD50	*RAD51*	*RAF1*	*RARA*	*RB1*	*RET*	*RHOA*
ROBO1	*ROS1*	*RSPO2*	*RUNX1*	*RUNX1T1*	*SDHA*	*SDHB*
SDHC	*SDHD*	*SETD2*	*SF3B1*	*SHC1*	*SHOC2*	*SLC7A8*
SMAD2	*SMAD3*	*SMAD4*	*SMARCA2*	*SMARCA4*	*SMARCB1*	*SMO*
SOCS1	*SOS1*	*SOX2*	*SOX9*	*SPEN*	*SPOP*	*SPRED1*
SPRY4	*SRC*	*STAG2*	*STAT3*	*STK11*	*SUFU*	*SYK*
SYNE1	*TCF3*	*TCF7L2*	*TERT*	*TET1*	*TET2*	*TET3*
TFAP2C	*TGFBR2*	*TIAM1*	*TNFAIP3*	*TNFRSF14*	*TOP1*	*TP53*
TRAF7	*TRIM60*	*TSC1*	*TSC2*	*TSHR*	*U2AF1*	*USP28*
VHL	*WT1*	*XPC*	*XPO1*	*XRCC2*	*ZMYM3*	*ZNF2*
ZNF518B	*ZRSR2*					
